# EZH2 engages TGFβ signaling to promote breast cancer bone metastasis via integrin β1-FAK activation

**DOI:** 10.1038/s41467-022-30105-0

**Published:** 2022-05-10

**Authors:** Lin Zhang, Jingkun Qu, Yutao Qi, Yimin Duan, Yu-Wen Huang, Zhifen Zhou, Ping Li, Jun Yao, Beibei Huang, Shuxing Zhang, Dihua Yu

**Affiliations:** 1 Department of Molecular and Cellular Oncology, The University of Texas MD Anderson Cancer Center, Houston, TX USA; 2 MD Anderson Cancer Center UTHealth Graduate School of Biomedical Sciences, Houston, TX USA; 3 Intelligent Molecular Discovery Laboratory, Department of Experimental Therapeutics, The University of Texas MD Anderson Cancer Center, Houston, TX USA; 4 Present Address: Department of Oncology, The Second Affiliated Hospital of Xi’an Jiaotong University, Xi’an, Shaanxi China; 5 Present Address: Department of Pharmacology, School of Medicine, China Medical University, Taichung, Taiwan

**Keywords:** Breast cancer, Bone metastases, Growth factor signalling, Phosphorylation

## Abstract

Bone metastases occur in 50–70% of patients with late-stage breast cancers and effective therapies are needed. The expression of enhancer of zeste homolog 2 *(*EZH2) is correlated with breast cancer metastasis, but its function in bone metastasis hasn’t been well-explored. Here we report that EZH2 promotes osteolytic metastasis of breast cancer through regulating transforming growth factor beta (TGFβ) signaling. EZH2 induces cancer cell proliferation and osteoclast maturation, whereas EZH2 knockdown decreases bone metastasis incidence and outgrowth in vivo. Mechanistically, EZH2 transcriptionally increases *ITGB1*, which encodes for integrin β1. Integrin β1 activates focal adhesion kinase (FAK), which phosphorylates TGFβ receptor type I (TGFβRI) at tyrosine 182 to enhance its binding to TGFβ receptor type II (TGFβRII), thereby activating TGFβ signaling. Clinically applicable FAK inhibitors but not EZH2 methyltransferase inhibitors effectively inhibit breast cancer bone metastasis in vivo. Overall, we find that the EZH2-integrin β1-FAK axis cooperates with the TGFβ signaling pathway to promote bone metastasis of breast cancer.

## Abstract (web summary)

Breast cancer cells are known to metastasize to the bone but why the cells should migrate and metastasize to this particular organ is not clearly understood. Here, the authors show that EZH2 activates an integrin B1 and FAK signaling pathway in breast cancer cells, which activates TGFB signaling to drive metastasis in the bone.

## Introduction

Breast cancer is the most commonly diagnosed cancer in female individuals worldwide^1^. About 50–70% of breast cancer patients with late-stage disease develop bone metastases that cause skeletal-related events, including pain, pathological fractures, spinal cord compression, hypercalcemia, and other complications^2^. The treatments for bone metastasis are limited and merely palliative; standard antiresorptive agents, chemotherapy, and radiotherapy can delay or lessen skeletal-related events, but they cannot cure bone metastasis^3^. Exploring the molecular mechanism of bone metastasis comprehensively may provide additional therapeutic strategies for patients with bone metastasis. Breast cancer bone metastasis frequently induces osteolytic lesions, which lead to massive bone resorption and bone fractures^4^. Osteolytic bone resorption causes secretion of several growth factors, including transforming growth factor beta (TGFβ). Bone metastasis is incited by “the vicious cycle”, which designates the feed-forward cycle among cancer cells, osteoblasts, and osteoclasts in promoting both uncontrolled tumor growth and osteoclast activity^4–6^.

TGFβ plays dual roles in cancer initiation and progression: it works as a tumor suppressor in premalignant cells but induces breast cancer metastasis by enhancing epithelial–mesenchymal transition, angiogenesis, and immunosuppression^7,8^. Studies have well established that TGFβ is a predominant cytokine driving the feed-forward vicious cycle to promote metastatic cancer cell growth in bones^9^. In canonical TGFβ signaling, active TGFβ binds to its receptor, TGFβ receptor type II (TGFβRII), which binds and activates TGFβ receptor type I (TGFβRI) on the cell membrane. TGFβRI phosphorylates downstream signaling molecules Smad2/3, which form a complex with Smad4; the Smad2/3/4 complex is then translocated to the nucleus. The nuclear Smad2/3/4 complex works as transcription factors to turn on the transcription of target genes^10,11^. Noncanonical TGFβ signaling works as a Smad-independent pathway through activation of p38 mitogen-activated protein kinase (MAPK), extracellular signal-regulated kinase (ERK), c-Jun N-terminal kinase (JNK), or phosphoinositide 3-kinase (PI3K)/AKT signaling^12^.

EZH2 is a histone methyltransferase that serves as an enzymatic subunit of the polycomb repressive complex 2^13^. It regulates gene expression through trimethylation of histone H3 at lysine (K) 27 (H3K27me3) or as a transcription co-factor^13^. Overexpression of EZH2 is correlated with metastasis of solid tumors such as prostate and breast cancers^14,15^ and is considered a prognostic biomarker of metastasis risk in women with early-stage hereditary breast cancer^16^. It is reported that EZH2 was highly expressed in tissues of renal cell carcinoma obtained from patients who had bone metastases^17^, suggesting that EZH2 promotes cancer cell bone metastasis. However, the function of EZH2 in the vicious cycle of breast cancer bone metastasis is unknown.

Here we found that depletion of EZH2 blocked breast cancer bone metastasis in vivo. Under TGFβ stimulation, EZH2 increased the level of pS465/467-Smad2 and the expression of parathyroid hormone-like hormone (PTHLH, also named parathyroid hormone-related protein, PTHRP), two key effectors of the canonical TGFβ pathway. Mechanistically, EZH2 increases the transcription of integrin β1-encoding *ITGB1* that activates a downstream effector, focal adhesion kinase (FAK). Activated FAK phosphorylates TGFβRI and enhances the binding of TGFβRI to TGFβRII to activate the TGFβ signaling pathway. Our study revealed the cooperation between EZH2 and TGFβ signaling in promoting bone metastasis of breast cancer through a methyltransferase-independent mechanism, and demonstrated that targeting FAK may be an effective strategy for treatment of EZH2-induced breast cancer bone metastasis.

## Results

### EZH2 promotes breast cancer bone metastasis, which cannot be blocked by an EZH2 methyltransferase inhibitor

**Fig. 1:**
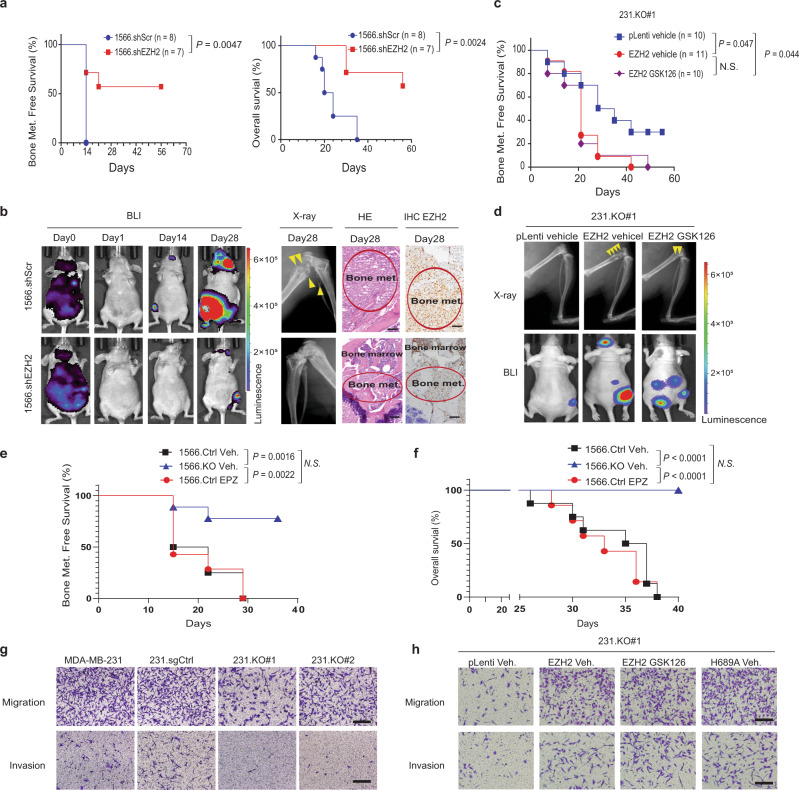
EZH2 promotes breast cancer bone metastasis, which cannot be blocked by an EZH2 methyltransferase inhibitor. **a** Kaplan–Meier curves showing bone metastasis-free survival and overall survival rates in mice intracardially injected with 1 × 10^5^ 1566.shCtrl (*n* = 8) or 1566.shEZH2 (*n* = 7) cells. Log-rank test. **b** Representative bioluminescence (BLI), X-ray, and hematoxylin and eosin (HE)-stained images of bone-metastatic lesions in the 2 subgroups described in (**a**) obtained at the indicated time points. The arrows indicate osteolytic bone lesions in X-ray images. EZH2 expression is shown by IHC staining. Scale bars, 50 μm. **c** Kaplan–-Meier curves showing bone metastasis-free survival rates in mice intracardially injected with 1 × 10^5^ 231.KO#1.pLenti cells and given treatment with a vehicle (231.KO#1.pLenti vehicle*, n* = 10) or 1 × 10^5^ 231.KO#1.EZH2 cells and given treatment with a vehicle (231.KO#1.EZH2 vehicle*, n* = 11) or GSK126 (231.KO#1.EZH2 GSK126; *n* = 10; 150 mg/kg GSK126 per mouse, i.p. injection three times a week). Log-rank test. **d** Representative X-ray and bioluminescence (BLI) images of bone-metastatic lesions in the three subgroups described in **c**. The arrows indicate osteolytic bone lesions in X-ray images. **e**, **f** Kaplan–Meier survival analysis showing bone metastasis-free survival (**e**) and overall (**f**) survival rates in nude mice intracardially injected with luciferase-labeled 1 × 10^5^ 1566.Ctrl and treated with vehicle (*n* = 8) or EPZ6438 (*n* = 7), or injected with 1566.KO cells and treated with vehicle (*n* = 9). Log-rank test. **g** Representative images of invading and migrating MDA-MB-231, 231.sgCtrl., 231.KO#1, and 231.KO#2 cells. Scale bars, 100 μm. **h** Representative images of invading and migrating 231.KO#1.pLenti. cells treated with vehicle, 231.KO#1.EZH2 cells treated with vehicle, 231.KO#1.EZH2 cells treated with 2 μM GSK126, and 231.KO#1.H689A cells treated with vehicle. Scale bars, 100 μm. All *P* values are indicated in the figures.

To explore the function of EZH2 in bone metastasis of breast cancer, we transfected either EZH2 shRNA or control shRNA into the MDA-MB-231 bone-seeking 231–1566 cell subline that expresses GFP and luciferase^7^ to generate the EZH2-knockdown cell lines 1566.shEZH2 and its control cell line 1566.shScr, respectively (Supplementary Fig. 1a). The sublines 1566.shEZH2 and 1566.shScr were injected, separately, into the left ventricles of nude mice. Mice injected with 1566.shEZH cells had significantly longer bone metastasis-free survival (*P* = 0.0047) and overall survival (*P* = 0.0024) than did mice injected with 1566.shScr cells (Fig. 1a). Bioluminescence imaging (BLI), X-ray imaging, and hematoxylin and eosin (H&E) staining of bone lesions all showed that mice injected with 1566.shEZH cells had fewer bone metastases than did mice injected with 1566.shScr cells on the same day post-injection (Fig. 1b). Using the CRISPR/CAS9 system, EZH2-knockout MDA-MB-231 cell subclones (231.KO) and their control clone (231.sgCtrl) were also generated (Supplementary Fig. 1b), and one of the 231.KO subclones (231.KO#1) were stably re-expressed with wild-type EZH2 (231.KO#1.EZH2) or a pLenti control vector (231.KO#1.pLenti) (Supplementary Fig. 1c). The derived sublines 231.KO#1.EZH2 and 231.KO#1.pLenti were intracardially injected into nude mice, respectively. Mice injected with 231.KO#1.EZH2 cells were treated with a vehicle or GSK126, a potent small-molecule EZH2 methyltransferase inhibitor, whereas the control mice injected with 231.KO#1.pLenti were only treated with a vehicle. The results showed that the vehicle-treated 231.KO#1.EZH2 group had significantly poorer bone metastasis-free survival rates than did the control 231.KO#1.pLenti group (Fig. 1c, d). Unexpectedly, the GSK126-treated 231.KO#1.EZH2 group had similar metastasis-free survival rate to that in the vehicle-treated 231.KO#1.EZH2 group (Fig. 1c, d). The data indicated that EZH2 overexpression increased the incidence of bone metastasis, which cannot be deterred by inhibiting EZH2 methyltransferase function with GSK126. Furthermore, we knocked out EZH2 in the 231–1566 cell subline, generated EZH2-knockout single clones #1 and #2 (Supplementary Fig. 1d) and mixed them together as the 1566.KO cell subline. We labeled 1566.KO cells and their control cells 1566.Ctrl with GFP and luciferase, and injected them intracardially into mice to generate bone metastases. Mice injected with 1566.KO cells had significantly longer bone metastasis-free survival (*P* = 0.0016) and overall survival (*P* < 0.0001) than did mice injected with 1566.Ctrl cells (Fig. 1e, f, Supplementary Fig. 1e), and the data echo that of EZH2 shRNA knockdown in 231–1566 cells (Fig. 1a, b). EPZ-6438 (Tazemetostat) is another potent, and selective EZH2 methyltransferase inhibitor^18^, which is under several clinical trials (ClinicalTrials.gov Identifier: NCT01897571, NCT03009344) for the treatment of advanced solid tumors or lymphomas. Those 1566.Ctrl cell-injected mice were also treated with EPZ-6438 (250 mg/kg, twice/day, oral), or vehicle beginning at day 6 post-injection. Similar to findings from GSK126-treated mice bearing 231.KO#1.EZH2 bone metastasis, EPZ-6438 treatment did not deter bone metastasis incidence and progression of 1566.Ctrl cell-injected mice as there was no significant difference in the bone metastasis-free survival, overall survival and BLI images between the vehicle- versus EPZ-6438-treated groups (Fig. 1e, f, Supplementary Fig. 1e). Additionally, we pooled five of MDA-MB-231 EZH2 knockout single clones together and labeled them with GFP and luciferase (231.KO mixed) (Supplementary Fig. 1f), which were intratibially injected into nude mice with 231 cells as controls. Clearly, EZH2 knockout significantly inhibited bone metastasis outgrowth (Supplementary Fig. 1g, h). The above loss- and gain-of EZH2 function in vivo bone metastasis experiments demonstrated that EZH2 promoted breast cancer bone metastasis and that EZH2’s effect on bone metastasis is likely methyltransferase-independent.

To explore the mechanism of EZH2 promotion of bone metastasis, we first compared the proliferation, migration, and invasion ability of high and low EZH2-expressing MDA-MB-231 cell sublines. High EZH2-expressing cells (MDA-MB-231 and 231.sgCtrl) and low EZH2-expressing cells (231.KO #1 and #2) had similar rates of proliferation in two-dimensional cell culture as measured using MMT assays (Supplementary Fig. 1i). However, high EZH2-expressing cells had greater migration and invasion ability in vitro than did low EZH2-expressing cells (Fig. 1g, Supplementary Fig. 1j). GSK126 treatment didn’t change cell proliferation, migration, or invasion of high EZH2-expressing MDA-MB-231 cells compared to vehicle treatment (Supplementary Fig. 1k–m). Additionally, we introduced an EZH2-methyltransferase-dead mutant EZH2-H689A^19^ into the 231.KO#1 cells to generate the stable 231.KO#1.H689A subline, along with the 231.KO#1.pLenti and 231.KO#1.EZH2 sublines (Supplementary Fig. 1c), to test EZH2 methyltransferase-independent function. Re-expression of the wild-type EZH2 (231.KO#1.EZH2) greatly increased cell migration and invasion compared to the control 231.KO#1.pLenti cells (Fig. 1h, Supplementary Fig. 1n). And re-expression of methyltransferase-dead mutant EZH2 H689A in 231.KO#1 (231.KO#1.H689A) also promoted migration or invasion just like that of re-expression of wild-type EZH2 (231.KO#1.EZH2) (Fig. 1h, Supplementary Fig. 1n). Similarly, GSK126 treatment didn’t have any inhibitory effect on migration or invasion of 231.KO#1.EZH2 cells (Fig. 1h, Supplementary Fig. 1n). These data suggested that EZH2 promoted MDA-MB-231 cell migration and invasion but did not change cell proliferation in two-dimensional cell culture. Furthermore, blocking EZH2’s histone methyltransferase function, either genetically by EZH2 H694A mutation or with EZH2 methyltransferase inhibitor GSK126, did not inhibit the migration or invasive ability induced by wild-type EZH2.

To expand the investigation of EZH2’s effect on bone metastasis, we also established CRISPR/CAS9-mediated EZH2-knockout subclones in 4T1 mouse mammary tumor cells (4T1.KO #1 and #2) (Supplementary Fig. 1o) and examined their proliferation, migration, and invasion compared with those of the control 4T1 cells. Knocking out EZH2 inhibited 4T1 cell migration and invasion but did not have an apparent effect on cell proliferation (Supplementary Fig. 1p, q). GSK126 treatment of 4T1 cells did not result in different proliferation, invasion, or migration from untreated 4T1 cells (Supplementary Fig. 1p–r). Together, our data from both MBA-MD-231 and 4T1 cells showed that EZH2 promoted cancer cell migration and invasion, but this function is unlikely dependent on EZH2’s methyltransferase activity.

### EZH2 regulates the vicious cycle of breast cancer bone metastasis

**Fig. 2:**
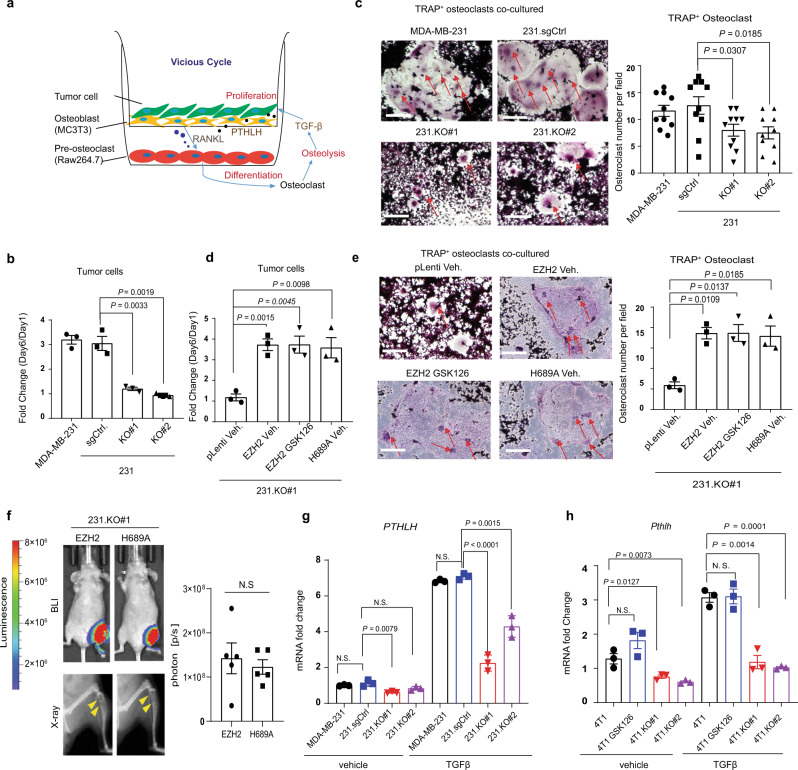
EZH2 regulates the vicious cycle of breast cancer bone metastasis. **a** Model of triple co-culture of breast cancer cells with preosteoclasts and osetoblasts. Murine RAW 264.7 preosteoclasts were seeded into the wells of six-well plates. GFP-labeled breast cancer cells and MC3T3 osteoblasts were seeded into Millicell Hanging Cell Culture Inserts (Millipore) in the six-well plates and treated with TGFβ (5 ng/mL). **b** Quantification of MDA-MB-231, 231.sgCtrl., 231.KO#1, and 231.KO#2 cells after co-culture with osteoclasts and MC3T3 osteoblasts treated with TGFβ (5 ng/mL) for 6 days. Data are presented as means ± S.E.M. *t*-test (two-sided). Three biologically independent experiments. **c** Representative staining images and quantification of mature TRAP^+^ osteoclasts after culture with MC3T3 osteoblasts and the indicated cancer cells, and treatment of them with TGFβ (5 ng/mL) for 6 days. The arrows indicate multinuclear mature TRAP^+^ osteoclasts. Scale bars, 200 μm. Data are presented as means ± S.E.M. *t*-test (two-sided). Ten random vision fields examined over three biologically independent experiments. **d** Quantification of 231.KO#1.pLenti. cells treated with vehicle, 231.KO#1.EZH2 cells treated with vehicle, 231.KO#1.EZH2 cells treated with 2 μM GSK126, 231.KO#1.H689A cells treated with vehicle, after co-culture with osteoclasts and MC3T3 osteoblasts treated with TGFβ (5 ng/mL) for 6 days. Data are presented as means ± S.E.M. *t*-test (two-sided). Three biologically independent experiments. **e** Representative staining images and quantification of mature TRAP^+^ osteoclasts after co-culture with MC3T3 osteoblasts and the indicated cancer cells and treatment of them with TGFβ (5 ng/mL) for 6 days. The arrows indicate multinuclear mature TRAP^+^ osteoclasts. Scale bars, 200 μm. Data are presented as means ± S.E.M. *t*-test, (two-sided). Three biologically independent experiments. **f** Representative bioluminescence image (BLI), X-ray images and quantification of BLI signals of bone-metastatic lesions in the two subgroups of mice (*n* = 5 in each group) intratibially injected with 231.KO#1.EZH2 or 231.KO#1.H689A cells 5 weeks post-injection. Data are presented as means ± S.E.M. *t*-test (two-sided). The arrows indicate osteolytic bone lesions in X-ray images. **g** qRT-PCR analysis of *PTHLH* mRNA expression in the indicated cells treated with a vehicle or TGFβ (5 ng/mL, 2 h). Data are presented as means ± S.E.M. *t*-test (two-sided). N.S. not significant. Three biologically independent experiments. **h** qRT-PCR analysis of *Pthlh* mRNA expression in the indicated cells treated with a vehicle or TGFβ (5 ng/mL, 2 h). Data are presented as means ± S.E.M. *t*-test (two-sided). Three biologically independent experiments. All *P* values are indicated in the figures. The TGFβ used in all experiments in this study is TGFβ1.

The colonization and growth of cancer cells in the bone marrow are critical for bone metastasis formation^6^. Since EZH2 knockout significantly inhibited bone metastasis outgrowth (Supplementary Fig. 1g, h), we explored the function of EZH2 in promoting metastatic breast cancer outgrowth in the bone. To mimic the vicious cycle of breast cancer bone metastasis microenvironment, we co-cultured breast cancer cells with RAW264.7 preosteoclasts and MC3T3 osteoblasts (triple co-culture) under TGFβ treatment (5 ng/mL) (Fig. 2a). The MDA-MB-231, 231.sgCtrl, 231.KO#1, and 231.KO#2 cells were pre-transfected with GFP expression vector for easy detection and quantification by flow cytometry (Supplementary Fig. 2a–c). Mature osteoclasts are detected by TRAP staining as round giant cells with three or more nuclei^20^ and they induce osteolysis to release TGFβ, which activates the vicious cycle of breast cancer bone metastasis^9^. Six days in triple co-culture, the EZH2-knockout 231.KO#1 and 231.KO#2 cells showed significantly inhibited cell growth than MDA-MB-231 and 231.sgCtrl cells (Fig. 2b and Supplementary Fig. 2c). Also, the RAW264.7 preosteoclasts that differentiated into mature osteoclasts were significantly less in co-culture with EZH2-knockout cells (231.KO#1 and 231.KO#2) than with MDA-MB-231 or 231.sgCtrl cells (Fig. 2c). When MDA-MB-231 cells were treated with 2 μM GSK126 or a vehicle (dimethyl sulfoxide, DMSO) in triple co-culture, GSK126 did not inhibit cancer cell proliferation nor osteoclast cell maturation (Supplementary Fig. 2d, e). To further test EZH2 methyltransferase function in vicious cycle of breast cancer bone metastasis, 231.KO#1.pLenti, 231.KO#1.EZH2, and 231.KO#1.H689A cells were compared in triple co-culture. Similar to the wild-type EZH2 re-expressing 231.KO#1.EZH2 cells, 231.KO#1.H689A cells that re-expressing EZH2 methyltransferase-dead mutant H689A had enhanced tumor cell proliferation and osteoclast maturation compared to 231.KO#1.pLenti cells (Fig. 2d, e). In addition, EZH2 re-expressing 231.KO#1.EZH2 cells with or without GSK126 treatment showed similarly increased tumor cell proliferation and osteoclast maturation compared to 231.KO#1.pLenti cells (Fig. 2d, e). Likewise, we performed triple co-culture experiments with 4T1 cells and EZH2-knockout 4T1 cell sublines (4T1.KO #1 and #2) as well as treating 4T1 cells with GSK126, and had consistent findings as those from the MDA-MB-231 cell sublines. Mainly, (i) high EZH2-expressing 4T1 cells possessed a growth advantage and induced osteoclasts maturation more than EZH2-knockout cells did; (ii) GSK126 didn’t inhibit 4T1 cell proliferation or RAW264.7 preosteoclasts maturation in the triple co-culture (Supplementary Fig. 2f, g).

Next, we examined whether EZH2 promoted bone metastasis outgrowth in vivo by increasing tumor cell proliferation or/and inhibiting apoptosis by IHC staining of Ki67 and cleaved caspase 3, which showed that the bone metastasis of EZH2 knockdown 1566.shEZH2 cells had significantly decreased proliferation and increased apoptosis compared to that of 1566.shScr cells (Supplementary Fig. 2h). Furthermore, we intratibially injected 231.KO#1.EZH2 and 231.KO#1.H689A cells into mice and monitored the bone metastasis outgrowth. We found that 231.KO#1.EZH2 and 231.KO#1.H689A cells induced bone metastasis lesions similarly (Fig. 2f). Together, data from both cell models and in vivo experiments indicated that EZH2 promoted the vicious cycle of breast cancer bone metastasis, which cannot be blocked by EZH2 methyltransferase inhibitor or the EZH2 H689A methyltransferase dead mutation.

Parathyroid hormone-like hormone (PTHLH, also named PTHRP) is an essential mediator of breast cancer bone metastasis, and metastatic cancer cells secrete PTHLH into the bone microenvironment to activate osteolysis^21^. Knockout of EZH2 in both MDA-MB-231 and 4T1 cells reduced their *PTHLH* mRNA expression under TGFβ treatment as measured by qRT-PCR (Fig. 2g, h), whereas GSK126 treatment of 4T1 cells didn’t reduce *Pthlh* mRNA expression (Fig. 2h). Besides PTHLH, IL-8 is a cytokine that also regulates osteolysis in breast cancer bone metastasis^22^. Knockout of EZH2 in MDA-MB-231 and 4T1 cells also inhibited their *IL-8* mRNA expression, but GSK126 treatment didn’t change it (Supplementary Fig. 2i, j). These data indicated that EZH2 facilitates *PTHLH* and *IL-8* expressions, which can mediate the vicious cycle of breast cancer bone metastasis.

### EZH2 increases pS465/467-Smad2 and pY397-FAK levels in response to TGFβ stimulation

**Fig. 3:**
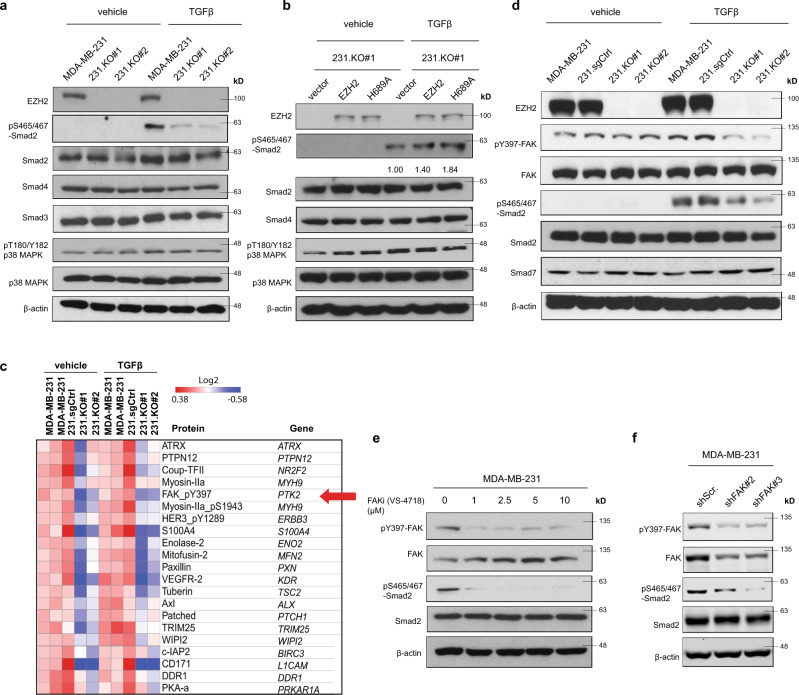
EZH2 increases pS465/467-Smad2 and pY397-FAK levels in response to TGFβ stimulation. **a** Western blotting of the expression of the indicated proteins in MDA-MB-231, 231.KO#1, and 231.KO#2 cells treated with a vehicle or TGFβ (5 ng/mL) for 2 h. **b** Western blotting of the expression of the indicated proteins in 231.KO#1.vector, 231.KO#1.EZH2, and 231.KO#1.H689A cells treated with a vehicle or TGFβ (5 ng/mL) for 2 h. **c** RPPA analysis of MDA-MB-231, 231.sgCtrl, 231.KO#1, and 231.KO#2 cells treated with a vehicle or TGFβ (5 ng/mL) for 2 h. The heatmap shows the top downregulated proteins in 231.KO#1 and 231.KO#2 cells compared with MDA-MB-231 and 231.sgCtrl cells. **d** Western blotting of the expression of the indicated proteins in MDA-MB-231, 231.sgCtrl, 231.KO#1, and 231.KO#2 cells treated with a vehicle or TGFβ (5 ng/mL, 2 h). **e** Western blotting of the expression of the indicated proteins in MDA-MB-231 cells treated with the FAKi VS-4718 at different concentrations (0–10 μM) and with TGFβ (5 ng/mL) for 2 h. **f** Western blotting of the expression of the indicated proteins in 231.shScr, 231.shFAK#2, and 231.shFAK#3 cells treated with TGFβ (5 ng/mL) for 2 h.

*PTHLH* is a well-known TGFβ downstream gene regulated by the p-Smad2/Gli2 transcription factor complex or p38 MAPK^7,23^. To further explore how EZH2 facilitates PTHLH and TGFβ signaling in breast cancer cells, we detected pS465/467-Smad2 and pT180/Y182-p38 MAPK levels in MDA-MB-231 sublines. In response to TGFβ stimulation, knockout of EZH2 in MDA-MB-231 cells inhibited pS465/467-Smad2 levels without significant changes of total Smad2, Smad3, or Smad4 protein expressions (Fig. 3a and Supplementary Fig. 3a). Knockout of EZH2 didn’t change the level of pT180/Y182-p38 MAPK, suggesting that EZH2 does not regulate TGFβ-p38 MAPK signaling (Fig. 3a). Knockdown of EZH2 by shRNAs (shEZH2#3 and shEZH#4) yielded similar results in MDA-MB-231 sublines (Supplementary Fig. 3b). To examine whether EZH2-methyltransferase function is involved in regulation of pS465/467-Smad2 levels, we measured pS465/467-SMAD2 levels in 231.KO#1 sublines that have re-expression of the control vector, wild-type EZH2, or H689A EZH2, in response to TGFβ (Fig. 3b). Like wild-type EZH2 re-expressing cells, H689A EZH2 re-expression also increased the level of pS465/467-Smad2 compared with the control vector (Fig. 3b). Furthermore, GSK126 treatment had no significant impact on increased pS465/467-Smad2 by TGFβ treatment (Supplementary Fig. 3c).

To gain insight into how EZH2 activates Smad2 signaling, we first measured the expressions of TGFβRI and TGFβRII, the TGFβ receptors upstream of pS465/467-Smad2, using flow cytometry or western blotting, and detected no significant changes in EZH2-knockout sublines (Supplementary Fig. 3d–f). Next, we performed reverse-phase protein array (RPPA) to profile protein expression changes in MDA-MB-231 and 231.sgCtrl cells versus those in EZH2-knockout MDA-MB-231 cells (231.KO #1 and #2) with or without TGFβ treatment. RPPA revealed that 228 proteins were downregulated and 194 proteins were upregulated in the two EZH2-knockout cell lines compared to that in MDA-MB-231 and 231.sgCtrl cells (Supplementary Data 1), and gene ontology molecular functional analysis showed that the dramatically downregulated and upregulated proteins were kinases, including tyrosine kinases (Fig. 3c, and Supplementary Fig. 3g, h). Notably, phosphorylation of tyrosine 397 on FAK (pY397-FAK) was significantly reduced in the EZH2-knockout cells (Fig. 3c). FAK is a non-receptor tyrosine kinase that regulates the survival, proliferation, migration, and invasion of cancer cells and can impact on cancer development and progression^24,25^. Unlike the FAK upstream kinase Src, whose function in bone metastasis is well-documented^26–28^, the function of FAK in bone metastasis is unclear. We thus validated RPPA data by western blotting, which showed that knocking out EZH2 in MDA-MB-231 and MCF7 cells inhibited both pY397-FAK and pS465/467-Smad2 levels under TGFβ treatment (Fig. 3d and Supplementary Fig. 3i). GSK126 treatment of MDA-MB-231 and 231–1566 cells with or without TGFβ stimulation didn’t change pY397-FAK or pS465/467-Smad2 levels compare to vehicle-treated cells (Supplementary Fig. 3j). Collectively, these data suggested that EZH2 functions to activate FAK and Smad2 signaling under TGFβ stimulation.

To examine whether increased pY397-FAK is related to enhanced pS465/467-Smad2, we treated MDA-MB-231 cells with FAK inhibitors (FAKi, VS-4718, or VS-6063) at different concentrations (1–10 μM), followed with TGFβ treatment (5 ng/mL, 2 h), then detected pS465/467-Smad2. FAKi treatment diminished pS465/467-Smad2 level and reduced *PTHLH* mRNA expression even under TGFβ stimulation (Fig. 3e and Supplementary Fig. 3k, l). Additionally, knocked down FAK using shRNAs (shFAK#2 or shFAK#3) in MDA-MB-231 cells also inhibited pS465/467-Smad2 levels (Fig. 3f). In the bone-seeking 231–1566 subline, knocking down FAK alone with siRNAs (siFAK#1 or siFAK#2) didn’t change pS465/467-Smad2 levels (Supplementary Fig. 3m); however, doubly knocking down FAK and its closely related kinase PYK2 (or FAK2) with siRNAs (siPYK2#44, siPYK2#49, or siPYK2#50) dramatically reduced pS465/467-Smad2 levels (Supplementary Fig. 3m). Doubly knocking down FAK and PYK2 also inhibited *PTHLH* mRNA expression (Supplementary Fig. 3n). These data indicated that activation of FAK family kinases by EZH2 increased the phosphorylation of S465/467-Smad2 and activated the TGFβ/Smad2/PTHLH pathway in breast cancer cells.

### pY397-FAK induces TGFβRI tyrosine phosphorylation that enhances its binding to TGFβRII in response to TGFβ

**Fig. 4:**
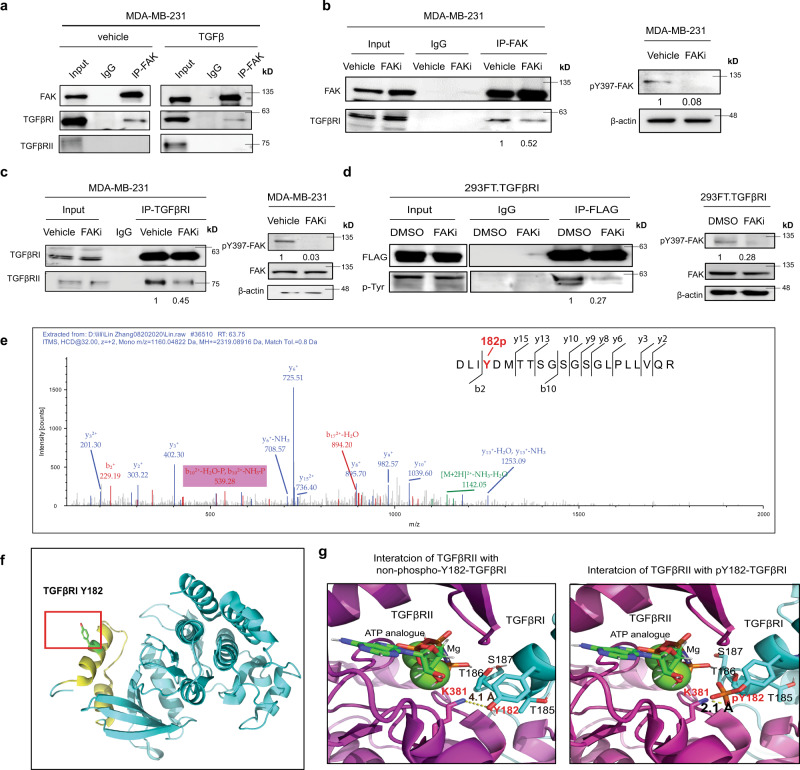
pY397-FAK induces TGFβRI tyrosine phosphorylation that enhances its binding to TGFβRII in response to TGFβ. **a** IP of FAK from the lysis of MDA-MB-231 cells treated with a vehicle or TGFβ (5 ng/mL) for 2 h followed by western blotting for FAK, TGFβRI, and TGFβRII. **b** IP of FAK from the lysis of MDA-MB-231 cells treated with a vehicle or the FAKi VS-4718 (1 μM) for 24 h followed by western blotting for FAK and TGFβRI. **c** IP of TGFβRI from the lysis of MDA-MB-231 cells treated with a vehicle or the FAKi VS-4718 (1 μM) for 24 h followed by western blotting for TGFβRI and TGFβRII. **d** IP of FLAG from the lysis of MDA-MB-231 cells treated with a vehicle or the FAKi VS-6063 (10 μM, 24 h), followed by western blotting for phospho-Tyrosine (p-Tyr). **e** Mass spectrometric analysis showing integrin β1 tyrosine (Y)–182 phosphorylation. **f** The orientation of Y182 was illustrated in the cartoon diagram of TBFβRI structure (PDB ID: 1ias) as generated using PyMOL. The GS domain of TBFβRI is colored in yellow. **g** Close-up view of the interaction interfaces of docked complex model between TGFβRII (PDB ID: 5e92) and TGFβRI (PDB ID: 1ias) without (top)/with (bottom) Y182 phosphorylation, obtained through protein–protein docking using ClusPro server.

We further explored the mechanism of how EZH2-mediated FAK activation induces pS465/467-Smad2. Smad7 can block the TGFβRI-induced pS465/467-Smad2^29^, but knocking down EZH2 did not change Smad7 expression (Fig. 3d). Thus, we assessed whether FAK regulates TGFβRI and TGFβRII expressions in MDA-MB-231 cells. Knocking down FAK didn’t change the protein expressions of TGFβRI or TGFβRII (Supplementary Fig. 4a, b). Next, we tested whether FAK can bind to Smad2 to phosphorylate Smad2 by immunoprecipitation (IP) of FAK followed with western blotting of Smad2, which didn’t show detectable binding (Supplementary Fig. 4c). Surprisingly, FAK IP brought down TGFβRI, not TGFβRII, with or without TGFβ exposure (Fig. 4a). Additionally, blocking FAK kinase activity by FAKi VS-4718 treatment reduced the binding of FAK to TGFβRI (Fig. 4b). Since TGFβ treatment induces TGFβRI binding to TGFβRII (Supplementary Fig. 4d) and, consequently, increased pS465/467-Smad2 levels, we postulated that pY397-FAK may phosphorylate TGFβRI that increases the binding affinity of TGFβRI for TGFβRII under TGFβ stimulation, leading to activation of Smad2 signaling. To test this, MDA-MB-231 cells were treated by the FAKi VS-4718 or had FAK knockdown by shRNAs (shFAK#21 or shFAK#3), and treated with TGFβ. After collecting cell lysates, we performed TGFβRI IP followed by western blotting of TGFβRII, which showed that FAK inhibition dramatically reduced the binding of TGFβRI to TGFβRII in response to TGFβ stimulation (Fig. 4c and Supplementary Fig. 4e, f). To explore whether FAK tyrosine kinase can phosphorylate TGFβRI, we performed IP to pull down TGFβRI from MDA-MB-231 cells (231.TGFβRI) or HEK 293FT cells transfected with exogenous FLAG-tagged wild-type TGFβRI (293FT.TGFβRI), then western blotting with anti-phospho-tyrosine antibodies. We detected tyrosine phosphorylation on TGFβRI (Supplementary Fig. 4g, h), which were reduced by FAKi VS-6063 treatment (Fig. 4d). The data indicated that activated FAK can induce tyrosine phosphorylation of TGFβRI. Next, we performed mass spectrometric analysis to locate the site of FAK-induced tyrosine phosphorylation on TGFβRI. We identified an unreported TGFβRI phosphorylation site at tyrosine 182 (pY182), which is located in the glycine and serine residues enriched-domain (GS domain)^30^ (Fig. 4e). Notably, it is known that after TGFβ binding, activated TGFβRII phosphorylates TGFβRI in the GS domain to activate TGFβRI kinase function and the transduction of TGFβ signals^31^.

Structural analysis revealed that Y182 of TGFβRI is highly exposed for potential phosphorylation (Fig. 4f), and it is close to two threonine and one serine sites (T185, T186, S187) in TGFβRI (Fig. 4g), which are bound and phosphorylated by TGFβRII^31,32^. Our protein docking suggests that Y182 of TGFβRI is oriented toward K381 of TGFβRII with a distance of 4.1 Å (Supplementary Fig. 4i left, and Fig. 4g left). Upon phosphorylation, the distance of the negatively charged phosphate group of TGFβRI to positively charged K381 of TGFβRII becomes much closer (2.1 Å), therefore, significantly enhancing binding of TGFβRI to TGFβRII through increased charge–charge interactions with positively charged K381, as well as Mg^2+^ coordinated with ATP (Supplementary Fig. 4i right and Fig. 4g right). Consequently, such increased TGFβRI binding to TGFβRII may further promote the phosphate transfer from the TGFβRII-bounded ATP to T185, T186, and S187 in the GS domain of TGFβRI (Fig. 4g). To determine whether phosphorylation of Y182 at TGFβRI changes the binding affinity of TGFβRI to TGFβRII, we stably expressed FLAG-tagged wild-type TGFβRI (RI-WT-FLAG), a non-phosphorylatable Y182F mutant TGFβRI (RI-YF-FLAG), or a phosphomimic Y182D mutant TGFβRI (RI-YD-FLAG) in HEK 293FT cells. The wild-type TGFβRI, mutant TGFβRI Y182F, or TGFβRI Y182D in these cells were pulled down with an anti-FLAG antibody after TGFβ treatment followed by western blotting of TGFβRII. We found that the non-phosphorylatable TGFβRI-Y182F mutant had reduced binding to TGFβRII, compared with wild-type TGFβRI and TGFβRI-Y182D mutant (Supplementary Fig. 4j, k); phosphomimic TGFβRI-Y182D mutant had slightly increased binding to TGFβRII, compared with wild-type TGFβRI (Supplementary Fig. 4l). Evidently, the phosphorylation of TGFβRI at Y182 enhanced TGFβRI binding to TGFβRII in response to TGFβ stimulation, which is consistent with our protein docking analysis.

### EZH2 increases the FAK upstream *ITGB1* expression

**Fig. 5:**
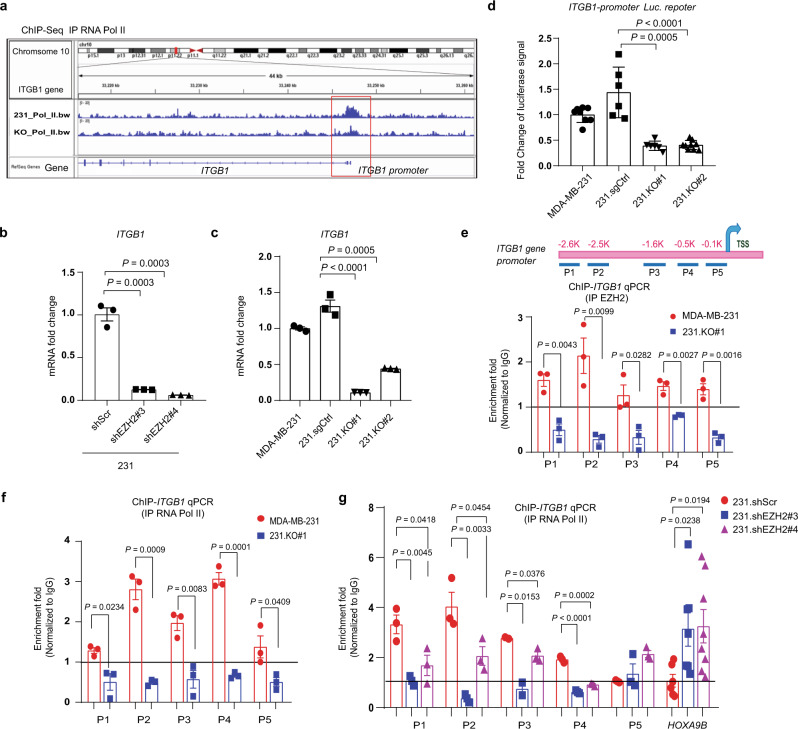
EZH2 increases the FAK upstream *ITGB1* expression. **a** Screenshot of the RNA Pol II ChIP sequencing (ChIP-seq) signal (GSE188640) at the *ITGB1* promoter locus in MDA-MB-231 (231_Pol _II.bw) and 231.KO#1 (KO_Pol_II.bw) cells. **b** qRT-PCR analysis of *ITGB1* mRNA expression in the indicated cells. Data are presented as means ± S.E.M. *t*-test (two-sided). Three biologically independent experiments. **c** qRT-PCR analysis of *ITGB1* mRNA expression in the indicated cells. Data are presented as means ± S.E.M. *t*-test (two-sided). Three biologically independent experiments. **d** *ITGB1* promoter activity in MDA-MB-231, 231.sgCtrl, 231.KO#1, and 231.KO#2 cells as measured using a dual-luciferase reporter assay. ITGB1 firefly luciferase signal was divided by the control renilla luciferase signal, and the ratios of luciferase/renilla in four cell lines were normalized to that of MDA-MB-231 cells. *n* = 6 biologically independent experiments in 231.sgCtrl and 231.KO#1 cells; *n* = 8 biologically independent experiments in MDA-MB-231 cells, *n* = 9 biologically independent experiments in 231.KO#2 cells. Data are means ± SEM, *t*-test (two-sided). **e** Top: the locations of the primers at the *ITGB1* gene promoter area for ChIP-qPCR. TSS, transcription start site. Bottom: EZH2 was immunoprecipitated from MDA-MB-231 and 231.KO#1 cells, and EZH2 binding to *ITGB1* in the cells was detected using qPCR with the indicated primers. All fold-enrichment values were normalized according to IgG values. Three biologically independent experiments. Data are presented as means ± S.E.M. *t*-test (two-sided). **f** RNA Pol II was immunoprecipitated from MDA-MB-231 and 231.KO#1 cells, and RNA Pol II binding to *ITGB1* in the cells was detected using qPCR with the indicated primers. All fold-enrichment values were normalized according to IgG values. Three biologically independent experiments. Data are presented as means ± S.E.M. *t*-test (two-sided). **g** RNA Pol II was immunoprecipitated from 231.shScr, 231.shEZH2#3, and 231.shEZH2#4 cells, and RNA Pol II binding to *ITGB1* or *HOXA9B* in the cells was detected using qPCR with the indicated primers. *HOXA9B* was used as a negative control. All fold-enrichment values were normalized according to IgG values. Three biologically independent experiments using *P1-P5* primers; six biologically independent experiments using *HOXA9B* primer. Data are presented as means ± S.E.M. *t*-test (two-sided). All *P* values are indicated in the figures.

FAK signaling is initiated by integrin-mediated cell adhesions. Integrins (such as β1 or β3) can facilitate FAK autophosphorylation at tyrosine 397, which increases the catalytic activity of FAK^24,33^. To understand the underlying mechanism of EZH2-induced pY397-FAK, we investigated whether and how EZH2 regulates expression of integrins β1 or β3. Since EZH2 methyltransferase activity is not required for increasing pY397-FAK (Supplementary Fig. 3j) and we recently reported that EZH2 can function as a transcription co-factor of RNA Pol II to upregulate mRNA transcription^34^, we examined whether EZH2 also regulate RNA Pol II transcription of genes encoding β1, β3, or other genes that may regulate pY397-FAK. We analyzed our chromatin IP sequencing (ChIP-seq) dataset (GSE188640) to compare RNA Pol II occupancy of gene promoters between EZH2-expressing MDA-MB-231 cells and 231.KO#1 cells in which EZH2 was knocked out^34^. The result showed that knocking out EZH2 led to reduced binding of RNA Pol II to promoter regions of at least 470 genes (Supplementary Data 2). Among these, binding of RNA Pol II to *ITGB1* (encoding integrin β1) promoter is substantially decreased, whereas binding to *ITGB3* (encoding integrin β3) showed little changes (Fig. 5a and Supplementary Fig. 5a). Consistently, qRT-PCR showed that knockdown and knockout of EZH2 downregulated *ITGB1* mRNA expression (Fig. 5b, c), resulting in decreased integrin β1 protein expression (Supplementary Fig. 5b, c), while knockdown of EZH2 had no significant effect on β3 mRNA expression (Supplementary Fig. 5d). Also, *ITGB1* promoter-driven luciferase reporter gene assays showed that *ITGB1* promoter activity was higher in EZH2-expressing MDA-MB-231 and 231.sgCtrl cells than that in EZH2-null cells (231.KO#1 and 231.KO#2) (Fig. 5d).

To detect the EZH2 and RNA Pol II bindings to the *ITGB1* promoter in MDA-MB-231 versus 231.KO#1 cells, we performed ChIP-qPCR using a series of PCR primers that bind to various regions of the *ITGB1* promoter from −2.6 kb upstream of (primer P1) to near (primer P5), the *ITGB1* transcription start site (Fig. 5e, top). In MDA-MB-231 cells, EZH2 was recruited to the *ITGB1* promoter from P1 to P5 loci, and expectedly, in 231.KO#1 cells, binding of EZH2 to these loci of *ITGB1* promoter was lost (Fig. 5e, bottom). Our ChIP-EZH2-qPCR assays showed that EZH2 binds to *ITGB1* promoter almost at the level of EZH2 binding to *HOXA9B*, a well-known methyltransferase substrate of EZH2^35^, with no binding to a non-substrate gene promoter (Supplementary Fig. 5e). Markedly, RNA Pol II bound well to P1 to P5 loci within the *ITGB1* promoter that overlapped with EZH2 binding loci in MDA-MB-231 cells, and RNA Pol II binding to the *ITGB1* promoter was also lost in EZH2-knockout 231.KO#1 cells (Fig. 5f), indicating EZH2 is required for RNA Pol II binding to the *ITGB1* promoter. Similarly, shRNA-mediated knocking down of EZH2 in 231.shEZH2#3 and 231.shEZH2#4 cells also reduced the RNA Pol II binding at P1 to P4 loci of the *ITGB1* promoter compared to control 231.shScr cells (Fig. 5g), which paralleled with the reduced EZH2 binding (Supplementary Fig. 5f). Expectedly, EZH2 knockdown in 231.shEZH2#3 and 231.shEZH2#4 cells reduced both EZH2 binding and H3K27me3 binding to, but increased RNA Pol II binding at, the *HOXA9B* promoter, compared to control 231.shScr cells; However, H3K27me3 binding to the *ITGB1* promoter were similar in control 231.shScr cells versus EZH2-knockdown cells (Supplementary Fig. 5g), further indicating EZH2 regulated *ITGB1* independent of its methyltransferase function. Taken together, both EZH2 and RNA Pol II bind to the same promoter regions of *ITGB1*, and EZH2 is likely functioning as a co-factor of RNA Pol II to upregulate *ITGB1* transcription independent of its methyltransferase function.

Since integrin β1 is responsible for FAK activation^24^ and activated FAK bound to and phosphorylated TGFβRI (Fig. 4a, d, e), we questioned whether integrin β1 can bind to TGFβRI in the same complex. IP integrin β1 followed by western blotting of TGFβRI and reverse IP TGFβRI followed by western blotting of integrin β1 explicitly showed that integrin β1 can bind to TGFβRI in MDA-MB-231 cells (Supplementary Fig. 5h, i), which further demonstrated the cross interactions between the TGFβ/TGFβRI pathway and the integrin β1/FAK pathway. Moreover, our molecular docking between TGFβRI/TGF-β/TGFβRII and integrin αVβ1 complex^36,37^ using the ClusPro web server showed that TGFβRI interacts with integrin αVβ1 among their ectodomains and this interaction requires TGFβ (Supplementary Fig. 5j). Interestingly, our IP of TGFβRI from untreated MDA-MB-231 cells followed with western blotting of integrin β1 showed that TGFβRI still can bind with integrin β1 without TGFβ (Supplementary Fig. 5k), conceivably via cytoplasmic domain. The binding between TGFβRI and integrin β1 without TGFβ stimulation was reduced by FAK inhibitor treatment (Supplementary Fig. 5l), implying that activated FAK can mediate the cytoplasmic binding of TGFβRI with integrin β1. These data suggested that the ectodomain of integrin β1 may bind with TGFβRI through TGFβ; whereas the cytoplasmic domain of integrin β1 may bind with TGFβRI through activated FAK (Supplementary Fig. 5m).

To further examine whether integrin β1 regulates Y182 phosphorylation of TGFβRI, we knocked down ITGB1 by siRNA in HEK 293FT cells expressing FLAG-tagged wild-type TGFβRI. After pulling down FLAG-TGFβRI, western blotting of tyrosine phosphorylation of TGFβRI detected a dramatic reduction by ITGB1 knockdown (Supplementary Fig. 5n), indicating that integrin β1, as an EZH2 downstream effector, regulates Y182-phosphorylation of TGFβRI. Furthermore, we investigated whether integrin β1 has a similar effect on the pS465/467-Smad2 level as EZH2 and pY397-FAK. We knocked down integrin β1 by two siRNAs or blocked integrin β1 signaling with antibodies in MDA-MB-231 cells, treated cells with vehicle or TGFβ, and detected significantly inhibited pS465/467-Smad2 levels by targeting integrin β1 (Supplementary Fig. 5o, p). Additionally, re-overexpressing ITGB1 in 231 EZH2 knockout subline or knockdown subline rescued the pS465/467-Smad2 level (Supplementary Fig. 5q, r). Both loss-of- and gain-of-ITGB1 function experiments indicated that integrin β1 can mediate EZH2’s regulatory function on TGFβ signaling. Moreover, IHC staining of integrin β1 and pY397-FAK in the bone metastases lesions of control 1566.shScr versus EZH2 knockdown 1566.shEZH2 cells (Fig. 1a, b) showed that EZH2 knockdown led to lower ITGB1 expression and pY397-FAK level in vivo (Supplementary Fig. 5s, t), which further demonstrated that integrin β1 and pY397-FAK are downstream effectors of EZH2.

### Treatment with a clinically applicable FAK inhibitor blocks EZH2-induced breast cancer bone metastasis

**Fig. 6:**
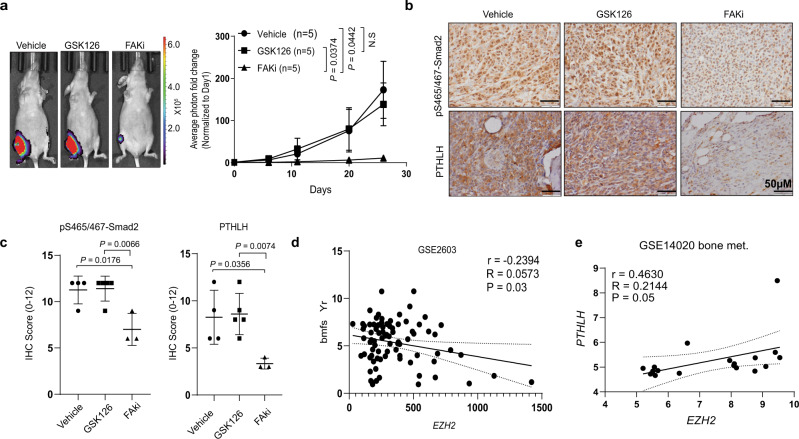
Treatment with a clinically applicable FAK inhibitor blocks EZH2-induced breast cancer bone metastasis. **a** Representative bioluminescence images of and quantification of the fold change in BLI intensity in the right leg region in three subgroups of mice (*n* = 5 in each group) intratibially injected with MDA-MB-231 cells and given treatment with a vehicle, GSK126 (100 mg/kg/day, i.p. injection), or the FAKi VS-6063 (50 mg/kg, twice a day, oral gavage) beginning on day 18 of injection. The BLI signal was normalized according to the signal on the first day after injection. Data are presented as means ± S.D. *t*-test (two-sided). **b**, **c** Representative pictures of IHC staining and quantification of pS465/467-Smad2 and PTHLP expression in the bone metastasis samples obtained from the three subgroups of mice in **a**. Data are presented as means ± S.E.M. *t*-test (two-sided). *n* = 4 bone metastasis slices in vehicle subgroup; *n* = *5* bone metastasis slices in GSK126 subgroup; *n* = *3* bone metastasis slices in FAKi subgroup. Scale bars, 50 μm. **d** The Pearson *r* correlation for *EZH2* RNA expression and bone metastasis-free survival years (bmfs Yr) in breast cancer patients (GSE2603 dataset). **e** The Pearson *r* correlation for *EZH2* and *PTHLH* RNA mRNA expression in bone metastasis samples obtained from breast cancer patients (GSE14020 dataset). All *P*-values are indicated in the figures.

Our above findings indicated that EZH2, via upregulating integrin β1 transcription, activated FAK, which activated the TGFβ/Smad2 pathway to increase bone metastasis. Our findings prompted us to test FAK inhibitor for treatment of bone metastases of high EZH2 expressing breast cancers. For bone metastasis outgrowth model, GFP- and luciferase-labeled MDA-MB-231 cells, which have relatively high EZH2 expression among tested breast cancer cell lines (Supplementary Fig. 6a), were intratibially injected into nude mice. We treated these mice with FAKi VS-6063 (50 mg/kg, twice a day, oral gavage), which is currently tested in clinical trials for treating patients with advanced lymphoma or solid tumors (ClinicalTrials.gov Identifier: NCT04439331, NCT03875820). To validate that EZH2-induced breast cancer bone metastasis outgrowth is independent of its methyltransferase function, a group of mice was treated with EZH2 methyltransferase inhibitor GSK126 (100 mg/kg, once a day, i.p. injection). The resulting bone metastasis outgrowth were detected using BLI, which confirmed that GSK126 treatment did not block tumor outgrowth in the bones (Fig. 6a). Excitingly, treatment with the FAKi VS-6063 significantly impeded the outgrowth of bone tumors compared to the control group (*P* = 0.0442) (Fig. 6a) and did not induce significant side effects (Supplementary Fig. 6b–e). IHC staining of bone metastasis showed that FAKi VS-6063, but not GSK126, significantly reduced pS465/467-Smad2 (*P* = 0.0066) level and PTHLH expression (*P* = 0.0074) in the bone metastases and both drugs effectively inhibited their targets (Fig. 6b, c and Supplementary Fig. 6f).

Finally, we examined the GSE2603 dataset and validated that EZH2 expression was negatively correlated with bone metastasis-free survival in breast cancer patients (*r* = −0.2394, *P* = 0.03) (Fig. 6d), suggesting that high EZH2 expression in primary breast tumors produces a high risk of developing bone metastasis in patients. We also examined the correlation between the expression of *EZH2* and the downstream effector *PTHLH* in bone metastasis tissues obtained from breast cancer patients in the GSE14020 dataset. We found that *EZH2* mRNA expression was positively correlated with *PTHLH* mRNA expression in patients’ bone metastases (*r* = 0.4630, *P* = 0.05) (Fig. 6e) but not in metastases to other organ sites (e.g., lung, liver, brain metastases; *r* = 0.2452, *P* = 0.097) (Supplementary Fig. 6g). This unique effect of EZH2 in promoting bone metastasis may result from dramatically higher TGFβ expression in bone metastasis than that in metastases of other organs and in primary mammary tumors (Supplementary Fig. 6h), i.e., the enriched TGFβ effectively activate the EZH2/integrin β1/FAK/p-Smad2 axis to upregulate *PTHLH* in bone metastasis. Most importantly, the clinical data confirmed that *EZH2* high expression can increase *PTHLH* expression that promotes bone metastasis in patients.

## Discussion

**Fig. 7:**
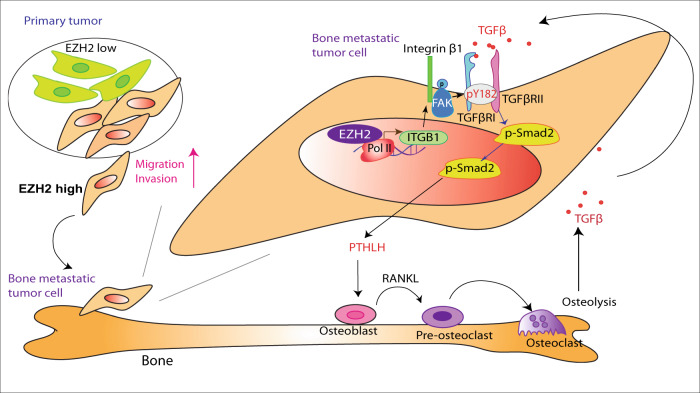
A model of EZH2’s interaction with TGFβ signaling in enhancing breast cancer bone metastasis. High EZH2-expressing cells have strong ability to invade and metastasize from the primary tumor to the bone. In bone-metastatic tumor cells, EZH2 functions as a transcription co-factor to increase ITGB1 transcription. Integrin β1 activates FAK and induces phosphorylation at Y397 of FAK, which phosphorylates TGFβRI at Y182. pY182-TGFβRI increases binding with TGFβRII, thereby activating pS465/467-Smad2, PTHLH expression, and the vicious cycle of breast cancer bone metastasis.

As described herein, we revealed a mechanism of how EZH2 promotes breast cancer bone metastasis. Specifically, EZH2 works as a transcription co-factor of RNA Pol II to increase *ITGB1* gene transcription; the increased integrin β1 induces phosphorylation of Y397 on FAK leading to FAK activation; activated pY397-FAK phosphorylates TGFβRI at Y182 that increases TGFβRI’s binding affinity for TGFβRII in response to TGFβ exposure, thereby triggering pS465/467-Smad2 that induces the downstream effector PTHLH; PTHLH accelerates osteolysis leading to more TGFβ release, and thus driving the feed-forward vicious cycle of breast cancer bone metastasis outgrowth (Fig. 7). Since FAK and TGFβ enhances epithelial–mesenchymal transition and cell migration^8,38^, EZH2-induced FAK/TGFβ signaling activation is also an underlying mechanism of the strong migration and invasion ability of EZH2 high expressing breast cancer cells. Activation of TGFβ signaling by FAK-induced phosphorylation has a critical and distinct effect in enhancing bone metastasis, partially due to the TGFβ-enriched bone microenvironment.

Although the function of TGFβ signaling in the bone metastasis of breast cancer is well-known, the cross-talk between the integrin/FAK and TGFβ pathways is not well-documented. Integrin β6 and β8 were reported to bind with TGFβ latency-associated peptide (LAP) and convert the latent TGFβ to the active form of TGFβ^39,40^. And it was reported that TGFβ activated FAK through integrin β3 or β1 and leading to p38 MAPK activation in renal cell carcinoma and hepato-carcinoma cells^41,42^. In the present study, we found that integrin β1-FAK is involved in the classical TGFβ/Smad-dependent pathway rather than the p38 MAPK pathway. Also, our data on the binding between integrin β1 and TGFβRI suggested that TGFβ may activate FAK through the TGFβRI- integrin β1 complex as targeting integrin β1 inhibited FAK activating under TGFβ treatment. Administering FAKi and genetically rendering FAK deficient in breast cancer cells abrogated the interaction between TGFβRI and TGFβRII and thereby blocked phosphorylation of Smad2 and expression of its downstream effector PTHLH. Our study demonstrated that the integrin/FAK and TGFβ/TGFβRI/TGFβRII pathways can cross talk and critically cooperate in driving the feed-forward vicious cycle of breast cancer bone metastasis.

It was reported that FAK and integrin β3 bind to TGFβRII in stellate hepatic cells^43^ and breast cancer cells^44^. Here, we uncovered that in EZH2 high expressing breast cancer cells, FAK and integrin β1 bound to TGFβRI rather than TGFβRII and that FAK phosphorylated TGFβRI. Little is known about tyrosine phosphorylation sites in TGFβRI and their functions, although several serine phosphorylation sites have been reported in TGFβRI^45^. Our mass spectrometry analysis identified a previously unreported tyrosine phosphorylation site at Y182 in the GS domain of TGFβRI. Our protein structure analysis and IP/western blotting experiments showed that the Y182 of TGFβRI is important for regulating the binding of TGFβRI to TGFβRII and subsequent TGFβ/Smad2 pathway activation. Interesting, Y182 is conserved in the GS domains of several activin type I receptors of the TGFβ superfamily, such as ALK4 and ALK7, but not in the GS domains of bone morphogenetic protein (BMP) type I receptors of the TGFβ superfamily, such as ALK2. However, it was reported that R206H mutation at the GS domain of ALK2, led to constitutive activation of this receptor and BMP signaling^46^, which can crosstalk to TGFβ signaling in bone formation^47^. This report and our findings indicated that modifications of the GS domain, either the activating mutation or the phosphorylation, is critical for TGFβ receptor activation and indicates the intricate regulations of downstream pathway.

EZH2 is a classic epigenetic protein that silences tumor suppressors through H3K27me3^13^. Recently, its noncanonical functions in the development of various cancers are gaining increasing attentions. For example, EZH2 can methylate non-histone substrates, such as Jarid2, STAT3, RORα, and PLZF, to regulate their transcription function or protein stability^48–51^. EZH2 also has functions independent of its histone methyltransferase activities. For example, EZH2 forms a complex with RelA and RelB to activate nuclear factor κB signaling in estrogen receptor-negative breast cancer cells^52^ and activates androgen receptor gene transcription through binding at the androgen receptor promoter in prostate cancer cells^53,54^. We recently reported that EZH2 can function as a methyltransferase-independent transcription factor to upregulate *c-JUN* expression that induced G-CSF to facilitate the brain infiltration of immunosuppressive neutrophils^34^. In the present study, we found that EZH2 upregulated *ITGB1* transcription in breast cancer cells by functioning as a transcriptional co-factor of RNA Pol II to facilitate its binding to the *ITGB1* promoter. Thus, *ITGB1* is a substrate regulated by EZH2 methyltransferase-independent activities.

EZH2 is highly expressed in various human malignancies and regulates tumor progression. Therefore, it is regarded as an attractive therapeutic target in cancer patients^55^. However, targeting EZH2 with methyltransferase inhibitors has not always proven to be beneficial in clinical trials^55–58^, partially because of the EZH2 methyltransferase-independent functions in promoting cancer development as mentioned above. In the present study, we found that small-molecule EZH2 inhibitors cannot block MDA-MB-231 cell-induced bone metastasis. However, targeting EZH2 downstream effector FAK with clinically applicable kinase inhibitors have striking effects on blocking breast cancer bone metastasis.

EZH2 inhibitors were reported to inhibit breast cancer lung metastasis in mouse models^59,60^, whereas EZH2 inhibitor EPZ-6438 failed to block but promoted bone metastasis in experimental models^61^. These contradictory effects of EZH2 on lung metastasis versus bone metastasis suggest that EZH2 inhibitors’ effects are depended on the tumor microenvironment. Bone metastasis is reported to have significantly lower EZH2 activity compared to the lung metastases^61^, suggesting that targeting EZH2 methyltransferase activity by EZH2 inhibitors in the unique bone microenvironment might not yield inhibitory efficacies on bone metastasis. In addition, the methyltransferase-independent EZH2-Integrin β1-FAK-TGFβ pathway identified in this study might be more predominant in the TGFβ-enriched bone metastasis microenvironment, but not in the lung metastasis microenvironment of lower TGFβ levels. Therefore, the efficacy of targeting EZH2 methyltransferase varies in different metastasis organs.

Since EZH2 plays distinct functions in different types of cancer and in metastases of different organs, targeting downstream effectors of EZH2, or EZH2’s enzyme function should be carefully evaluated. We found that FAK is a downstream effector of EZH2 in the vicious cycle of breast cancer bone metastasis. Thus, treatment with a FAKi combined with standard antiresorptive agents, chemotherapy, or radiotherapy may provide added benefit to breast cancer patients who suffer from bone metastasis.

## Methods

### Reagents and plasmids

Antibodies against EZH2 (#5246), H3 (#4499), pS465/467-Smad2 (#3108), Smad2 (#5339), Smad3 (#9513), Smad4 (#46535), pT180/Y182-p38 (#4511), p38 (#8690), pY397-FAK (#3283), FAK (#3285), FLAG (#14793) and cleaved caspase 3 (#9664) were purchased from Cell Signaling Technology. Antibodies against β-actin (A5441) was purchased from Sigma-Aldrich. Antibodies against TGFβRI (ab31013 for IP and western blotting), TGFβRII (ab184948), and Ki67 (ab15580) were purchased from Abcam. The antibody against TGFβRI (#ABF17-I for western blotting) and H3K27me3 (Millipore #07-449) were purchased from Millipore. Antibodies against integrin β1 (sc-8978, sc-9970), and IgG (sc-2025, sc-2027) were purchased from Santa Cruz Biotechnology. Antibodies against RNA Pol II (NB200-598) were purchased from Novus Biologicals. The antibody against phospho-tyrosine (#610000) was purchased from BD Biosciences. Antibodies against PTHLH (#MAB6734) and Smad7 (#MAB2029) were purchased from R&D Systems. The antibody against TGFβ1 (LS-B14345) was purchased from LSBio Company. The dilutions of all antibodies for western blotting are 1:1000, except the dilution of β-actin is 1:5000. Antibodies used for IP or ChIP: FLAG dilution is 1:100, IgG dilution is the same as that of the target antibodies, FAK dilution is 1:100, TGFβRI dilution is 1:100, EZH2 dilution is 1:100, RNA Pol II dilution is 1:100, H3K27me3 dilution is 1:200, pY397-FAK dilution is 1:100, and integrin β1 dilution is 1:100. Antibodies used for IHC: cleaved caspase 3 dilution is 1:1000, Ki67 dilution is to 1 μg/ml; PTHLH dilution is to 10 μg/ml, pY397-FAK dilution is 1:100, pS465/467-Smad2 dilution is 1:100, H3K27me3 is 1:200, and TGFβ1 dilution is to 10 μg/ml.

GSK126 (#15415), VS-6063 (#17737), and VS-4718 (#17668) were purchased from Cayman Chemical. GSK126 (#HY-13470) and VS-6063 (#HY-12289) were purchased from MedChemExpress. EPZ-6438 (#1463254-99-8) were purchased from TargetMol. TGFβ (#4342-5) was purchased from BioVision. A leukocyte acid phosphatase kit (#387 A) for tartrate-resistant acidic phosphatase (TRAP) staining was purchased from Sigma-Aldrich. LipoD293 DNA in vitro transfection reagent (#SL100468) and pepMute small interfering RNA (siRNA) transfection reagent (#SL100566) were purchased from SignaGen.

A pLenti-HA-EZH2 lentiviral vector was provided by Dr. Mien-Chie Hung^34^. Lentiviral-based pLKO.1 short hairpin RNAs (shRNAs) targeting EZH2 (shEZH2#3 [TRCN0000040076] and shEZH2#4 [TRCN0000010475]) and FAK (shFAK#2 [TRCN0000121211], shFAK#3 [TRCN0000121318], and shFAK#21 [TRCN0000121321]) were purchased from Sigma-Aldrich. pRK5-TGFβRI-FLAG plasmid (#14833), pCMV5B- TGFβRII plasmid (#11766), and EFIa-ITGB1 (#115799) were purchased from Addgene.

### Cell lines and cell culture

The human breast cancer cell lines MDA-MB-231 and MCF7 and mouse mammary tumor cell line 4T1 were purchased from the ATCC. The MDA-MB-231 subline 231–1566 was provided by Dr. Hung’s lab^7,62^. HEK 293FT cells were purchased from Thermo Fisher Scientific. These cell lines were further characterized by The University of Texas MD Anderson Cancer Center Cytogenetics and Cell Authentication Core and were tested for mycoplasma contamination. Cancer cells were cultured in Dulbecco’s modified Eagle’s medium/F12 medium supplemented with 10% fetal bovine serum (FBS; Thermo Fisher Scientific; #SH3007103). The murine osteoblast cell line MC3T3 was obtained from Dr. Sue-Hwa Lin’s lab^7^ and maintained in α-minimum essential medium with 10% FBS. The murine preosteoclast cell line RAW 264.7, obtained from the ATCC, was maintained in Dulbecco’s modified Eagle’s medium/high-glucose medium with 10% FBS for regular culture.

### RNA interference, transient transfection, CRISPR/CAS9 knockout cell line generation

Transient transfection of siRNAs into cancer cells was performed using pepMute siRNA Transfection Reagent (SignaGen; #SL100566). SiRNAs targeting FAK (siFAK#1 [SASI_Hs01_00035697] and siFAK#2 [SASI_Hs01_00035698]), proline-rich tyrosine kinase 2 (PYK2; siPYK2#44 [SASI_Hs01_00207544], siPYK#49 [2SASI_Hs01_00032249], and siPYK2#50 [SASI_Hs01_00032250]), and ITGB1 (siITGB1#1 [SASI_Hs01_00159474] and siITGB1#2 [SASI_Hs02_00333437]) were purchased from Sigma-Aldrich. In transient transfection of HEK 293FT cells with pRK5-TGFβRI-FLAG plasmid (#14833) or pCMV5B- TGFβRII plasmid (#11766) using Lipofectamine 3000 transfection kit (Invitrogen, #L3000-008).

For lentivirus production, shRNA gene knockdown or gene overexpression lentiviral vectors were transfected into HEK 293FT cells together with a packaging plasmid (psPAX2; Addgene; #12260) and envelope plasmid (pMD2G; Addgene; #12259) using LipoD293 reagent (SignaGen; SL100668) according to the manufacturer’s instructions. The lentiviruses were collected, filtered, and used to infect target cells in the presence of 8–10 μg/mL hexadimethrine bromide (Polybrene) for 24 h. The infected cells were selected using 300 μg/mL hygromycin (InvivoGen; #ant-hg-1) for 10 days or 2 μg/mL puromycin (InvivoGen; #ant-pr-1) for 4 days to generate stable cell lines.

Gene-knockout cell lines were established as described previously^34^. Briefly, to generate EZH2-knockout (KO) MDA-MB-231, 4T1, 231.1566 cells, pSpCAS9 (BB)-2A-Puro (PX459) V2 plasmids (Addgene; #62988) were used following the protocol described by Ran et al.^63^. A single guide RNA-targeting EZH2 was designed using the online CRISPR Design Tool (http://tools.genome-engineering.org) with the following primers: F5'-CACCGTGGTGGATGCAACCCGCAA-3' and R5'-AAACTTGCGGGTTGCA TCCACCAC-3'. Single guide RNA-targeting EZH2 oligos underwent annealing and were inserted into a pSpCAs9 (BB)-2A-Puro (PX459) V2 plasmid followed by transformation of the plasmid into a Stbl3-competent *Escherichia coli* strain. The plasmids extracted from *E. coli* colonies were sequence-verified and transfected into cancer cells using Lipofectamine 2000 (Life Technologies; #11668030). After puromycin selection, single cells were expanded into subclones. EZH2 protein expression in the cells was detected using western blotting, and EZH2 DNA modifications were validated via DNA sequencing.

### Site-specific mutation

Site-specific mutation was performed using a Q5 Site-Directed Mutagenesis Kit (New England BioLabs; #E0554S). EZH2 H689A mutation was using the primers F5'- CAAATGCTTCGGTAAATCCAAACTGC-3' and R5'- CCGAAGCATTTGCAAAACGAATTTTG-3'. TGFBRI-Y182F mutation was using the primers F5'- AGATTTAATTTTTGATATGACAACATCAGGG-3' and R5'- TTTAAGGTGGTGCCCTCT-3'. TGFBRI-Y182D mutation was using the primers AGATTTAATTGATGATATGACAACATCAGG-3' and R5'- TTTAAGGTGGTGCCCTCT-3'.

### Western blotting and immunoprecipitation

Western blotting and immunoprecipitation (IP) were performed as described previously^64^. Briefly, for western blotting, cells were lysed in lysis buffer (5 M urea, 10% sodium dodecyl sulfate [SDS], DNase-free water: 1:1:1) and then sonicated. The lysates were collected for western blotting analysis. Proteins were separated using SDS-polyacrylamide gel electrophoresis and transferred onto a polyvinylidene difluoride membrane. After each membrane was blocked with 5% milk for 1 h, it was probed with various primary antibodies overnight at 4 °C followed by incubation with secondary antibodies for 1 h at room temperature before being visualized with enhanced chemiluminescence reagent. For IP, cells were washed twice with phosphate-buffered saline and scraped into IP lysis buffer (1% Triton X-100, 150 mM NaCl, 10 mM Tris, pH 7.4, 1 mM EGTA, 1 mM EDTA, 0.5 mM sodium orthovanadate, 0.4 mM phenylmethanesulfonyl fluoride, 0.5% NP-40). The total cell lysates were precleared via incubation with protein G-linked agarose beads (Sigma; #1124323300) for 2 h at 4 °C. After preclearing, lysates were incubated with the primary antibody overnight at 4 °C and then with protein G-linked agarose beads for 1 h at 4 °C. The beads were washed with IP buffer three times, and the protein immunocomplex was extracted from agarose and detected using SDS-polyacrylamide gel electrophoresis and western blotting.

### Cell proliferation assays

Cell proliferation was measured using a 3-(4,5-dimethylthiazol-2-yl)-2,5-diphenyltetrazolium bromide (MTT) assay. Ten thousand MDA-MB-231 cells or 1000 4T1 cells per well (four wells per sample) were seeded in a 24-well plate, and the cell growth was examined via staining with MTT (Thermo Fisher Scientific; #M6496). The resulting intracellular purple formazan was solubilized using dimethyl sulfoxide and measured its absorbance on a plate reader at 570, measure also at 650 nm as reference wavelength, using a Gen5 microplate reader (BioTek); calculate the signal sample as OD570 minus OD650.

### Quantitative reverse transcription-polymerase chain reaction

Total RNA in cells was isolated using TRIzol reagent (Thermo Fisher Scientific; #15596026) and then reverse-transcribed using an iScript cDNA Synthesis Kit (Bio-Rad; #1708891). Quantitative reverse transcription (qRT)-polymerase chain reaction (PCR) analysis of cDNA expressions was conducted using SYBR FAST Universal qPCR Master Mix (Kapa Biosystems; #KK4602) with a StepOnePlus real-time PCR system (Applied Biosystems). Relative mRNA expression was quantified using the 2^−ΔΔCt^ method with logarithmic transformation. SYBR primers were obtained from Sigma or Integrated DNA Technologies (IDT). The following primers were used: human *PTHLH* (encoding PTHrP): F5'-TTTACGGCGACGATTCTTCC-3', R5'-TTCTTCCCAGGTGTCTTGAG-3'; mouse *Pthlh* (encoding PTHLH): F5'-CATCAGCTACTGCATGACAAGG-3', R5'-GGTGGTTTTTGGTGTTGGGAG-3'; human *interleukin-8* (*IL-8*; *CXCL8*): F5'-GAGTGATTGAGAGTGGACCACACT-3', R5'-AGACAGAGCTCTCTTCCATCAGAAA-3'; mouse *Il-8* (*Cxcl15*): F5'-TCCTGCTGGCTGTCCTTAAC-3', R5'-ACTGCTATCACTTCCTTTCTGTTG-3'; human *ACTB*: F5'-CATGTACGTTGCTATCCAGGC-3', R5'-CTCCTAATGTCACGCACGAT-3'; mouse *Actb*: F5'-TCCTCCTGAGCGCAAGTACTCT-3', R5'-CGGACTCATCGTACTCCTGCTT-3'. Human *ITGB1* (encoding integrin β1) primer #1 (H_ITGB1_2) and *ITGB3* (encoding integrin β3) primer #1 (H_ITGB3_1) were purchased from Sigma-Aldrich.

### Chromatin IP-quantitative PCR

Chromatin IP (ChIP)-quantitative PCR (qPCR) was performed as described previously^34^. Briefly, cells were fixed with 37% formaldehyde (final, 0.5%), treated with glycine (final, 125 mM), washed, resuspended in ChIP lysis buffer (50 mM Tris, pH 8.1, 10 mM EDTA, 1% SDS, 1% protease inhibitor cocktail, 10 mM phenylmethanesulfonyl fluoride), and sonicated. Lysates containing soluble chromatin were incubated with antibodies against EZH2, RNA Pol II, H3K27me3, or IgG overnight at 4 °C and then incubated for an additional 2 h at 4 °C with added protein G-linked agarose beads. The agarose bead-bound complexes were then washed, and the protein-chromatin complexes were extracted from the agarose beads with elution buffer. Reversal of the cross-linking of protein and DNA was performed by incubating the elution buffer with 10 mg/mL RNase A and 5 M NaCl overnight at 65 °C followed by incubation with 0.5 M EDTA, 1 M Tris, pH 6.5, and 10 mg/mL proteinase K for 1 h at 45 °C. Co-precipitated DNA was purified using a QIAquick spin column (QIAGEN), and 2 μL of DNA was analyzed via qPCR using specific primers for the *ITGB1* promoter, which is identified by DBTSS database (https://dbtss.hgc.jp/). The ChIP assay primers used were as follows: ChIP-*ITGB1*_1F: 5'- GCAAGCTCAGGCATAACAGC-3'; ChIP-*ITGB1*_1R: 5'-CCCTGGCTCAGAGAGAATGC-3'; ChIP-*ITGB1*_2F: 5'-AGCCCTTGAAGATGGAGGTCT-3'; ChIP-*ITGB1*_2R: 5'-AGACAATGAGGGCCATTTGTTTTT-3'; ChIP-*ITGB1*_3F: 5'- TTCTCGCAGCCATCTGCTAT-3'; ChIP-*ITGB1*_3R: 5'-GCCACTGGTTGCTGACTTGA-3'; ChIP-*ITGB1_*4F: 5'-CTGGATATGCTGGTCTGGGC-3'; ChIP-*IGB1*_4R: 5'-CCCAGAATCCATTCGTGCCT-3'; ChIP-*ITGB1*_5F: 5'-TGCGCTTTGACCAGTTAGGT-3'; ChIP-*ITGB1*_5R: 5'-GGAGCCTGACCATGAAGGAA-3'; *HOXA9B_*F: 5'-TCGCCAACCAAACACAACAGTC-3'; and *HOXA9B_*R: 5'-AAAGGGATCGTGCCGCTCTAC-3'. Negative control ChIP-*Neg*._F: 5'- CCTGGGAAGCTGCGGTTAAT-3'; ChIP-*Neg*._R: 5'- TGGACAAGTCGATCAGCTTCC-3'. All fold-enrichment values were normalized according to those of IgG. *HOXA9B* was used as a positive control for EZH2 and H3K27me3 binding.

### Triple co-culture assay and TRAP staining

Triple co-culture assay and TRAP staining were performed as described previously^7^. Murine RAW 264.7 preosteoclasts (3 × 10^4^ cells/well) were seeded directly into the wells of six-well co-culture plates, and MC3T3 cells (3 × 10^4^ cells/well) were seeded into Millicell Hanging Cell Culture Inserts (Millipore) in the six-well co-culture plates. The next day, MC3T3 cells attached to the membrane**s** of the inserts, and luciferase/green fluorescent protein (GFP)-labeled (GFP^+^) MDA-MB-231, 231.sgCtrl, 231.KO#1, or 231.KO#2 cells (3 × 10^4^ cells/well each) or 4T1, 4T1.KO#1, or 4T1.KO#2 cells (500 cells/well each) were added on top of the MC3T3 cell layer in triplicate and treated with 5 ng/mL TGFβ, 2 μM GSK126, or a vehicle. Co-culture assays were performed in Dulbecco’s modified Eagle’s medium/high-glucose medium supplemented with 10% FBS and that was changed every 2 days. TRAP staining of osteoclasts was performed on day 6 using a leukocyte acid phosphatase kit (Sigma-Aldrich; #387 A). TRAP^+^ multinucleated cells were scored as mature osteoclasts and quantified. MC3T3 cells and GFP^+^ tumor cells were trypsinized from the inserts and calculated GFP^+^ cell numbers using flow cytometry.

### Luciferase reporter assay

Luciferase reporter assay was performed as described previously^34^. pGL4.10 (Luc.2; E665A) was purchased from Promega. The pGL4.10-*ITGB1* reporter and a control Renilla luciferase vector were co-transfected into breast cancer cell lines using Lipofectamine 3000 transfection kit (Invitrogen; #L3000-008). After 48 h, luciferase activity was measured using a Dual-Luciferase Reporter Assay kit (Promega; E1910) with a 20/20 Luminometer (Turner Biosystems). The *ITGB1* promoter was generated via amplification of a genomic DNA sequence with PCR using the designed primers and then inserted upstream of the luciferase reporter gene in the pGL4.10 vector. The primer sequences for the *ITGB1*_reporter_1 were F5'-CGGGGTACCCTGGCTAATTTTTAGTAGAG-3' and R5'-CCGGATATC ACCTAACTGGTCAAAGCGCA-3'.

### Flow cytometry

For detecting TGFβRI on cell surfaces, breast cancer cells were seeded at the same density and collected when they reached 80–90% confluence. One million cells in each sample were washed twice in fluorescence-activated cell sorter (FACS) buffer (1% bovine serum albumin in phosphate-buffered saline), resuspended in 100 μL of FACS buffer, and stained with 1 μg of an anti-TGFβRI antibody or mouse anti-IgG antibody for 1 h, washed twice in FACS buffer, and stained with 1 μg of an APC anti-mouse secondary antibody for 1 h. Afterward, cells were washed with cold phosphate-buffered saline twice and analyzed using a FACSCanto II flow cytometer (BD Biosciences). For a triple co-culture assay, MC3T3 cells and GFP^+^ tumor cells were trypsinized from the inserts in tubes and washed twice in cold FACS buffer, resuspended in 400 μL of FACS buffer, and analyzed using a FACSCanto II flow cytometer.

### Migration and invasion assays

For a migration assay, breast cancer cells (30,000 cells/well) in FBS-free medium were placed on the top side of the uncoated membrane of transwell inserts and allowed to migrate through the pores, to the bottom side of the membrane. For an invasion assay, the top side of the membranes of transwell inserts were coated with 14.3% Matrigel for 1 h, and then breast cancer cells (30,000 cells/well) were loaded the same way as for the migration assay. A medium containing 10% FBS was added to the lower compartment of transwell as a chemical attractant. After culturing for 18 h in a migration assay or 24–30 h in an invasion assay, the migrated and invaded cells at the bottom side of the membrane of transwell inserts were stained with crystal violet and counted under a light microscope.

### Reverse-phase protein array

RPPA analysis of MDA-MB-231, 231.sgCtrl, 231.KO#1, and 231.KO#2 cells after treatment with a vehicle or 5 ng/mL TGFβ for 2 h was performed at the MD Anderson Functional Proteomics Reverse Phase Protein Array (RPPA) Core. Briefly, cellular proteins were denatured using 1% SDS, serially diluted, and spotted on nitrocellulose-coated slides. Each slide was probed with a validated primary antibody plus a biotin-conjugated secondary antibody. The signal obtained was amplified using a Dako Cytomation-catalyzed system and visualized in a DAB colorimetric reaction. Slides were scanned on a flatbed scanner to produce 16-bit tiff images. Spots from tiff images were identified and the density was quantified by Array-Pro Analyzer software. Each dilution curve was fitted using a logistic model (“SuperCurve Fitting” developed at MD Anderson)^65^ and normalized according to median polish. The heatmap included was generated in Cluster 3.0 (http://bonsai.hgc.jp/~mdehoon/software/cluster/software.htm) as a hierarchical cluster using Pearson Correlation and a center metric. The resulting heatmap was visualized in Treeview (http://www.eisenlab.org/eisen/) and presented as a high resolution.bmp format.

### Mass spectrometry

Liquid chromatography/tandem-mass spectrometry was used to identify phosphorylation sites of TGFβRI. HEK 293FT cells were transfected with pRK5-TGFβRI-FLAG plasmid (Addgene, #14833) using Lipofectamine 3000 transfection kit; HEK 293FT cell lysates were immunoprecipitated with an anti-FLAG antibody. After protein gel electrophoresis, TGFβRI band was excised from the gels and subjected to tryptic digestion. An aliquot of the tryptic digest (in 2% acetonitrile/0.1% formic acid in water) was analyzed by LC/MS/MS on an Orbitrap Fusion^TM^ Tribrid^TM^ mass spectrometer (Thermo Scientific^TM^) interfaced with a Dionex UltiMate 3000 Binary RSLCnano System. Peptides were separated onto an Acclaim^TM^ PepMap ^TM^ C_18_ column (75 µm ID × 15 cm, 2 µm) at flow rate of 300 nl/min. Gradient conditions were: 3–22% B for 40 min; 22–35% B for 10 min; 35–90% B for 10 min; 90% B held for 10 min,(solvent A, 0.1 % formic acid in water; solvent B, 0.1% formic acid in acetonitrile). The peptides were analyzed using data-dependent acquisition method; Orbitrap Fusion was operated with measurement of FTMS1 at resolutions 120,000 FWHM, scan range 350–1500 m/z, AGC target 2E5, and maximum injection time of 50 ms; During a maximum 3 s cycle time, the ITMS2 spectra were collected at rapid scan rate mode, with HCD NCE 34, 1.6 m/z isolation window, AGC target 1E4, maximum injection time of 35 ms, and dynamic exclusion was employed for 20 s. The raw data files were processed using Thermo Scientific^TM^ Proteome Discoverer^TM^ software version 1.4, spectra were searched against the Uniprot-Homo sapiens database using the Mascot search engine v2.3.02 (Matrix Science) run on an in-house server. Search results were trimmed to a 1% FDR for strict and 5% for relaxed condition using Percolator. For the trypsin, up to two missed cleavages were allowed. MS tolerance was set 10 ppm; MS/MS tolerance 0.8 Da. Carbamidomethylation on cysteine residues was used as fixed modification; oxidation of methionine as well as phosphorylation of serine, threonine, and tyrosine was set as variable modifications.

### TGFβRI-TGFβRII complex and Integrinβ1-TGFβRI complex modeling by molecular docking

High-resolution crystal structures of the cytoplasmic domain of TGFβRI (PDB ID: 1ias) and the kinase domain of TGFβRII (AMPPNP, an ATP analog, bound state, PDB ID: 5e92) were obtained from Protein Data Bank. A phosphoryl group was added to TGFβRI Y182 using PyTMs^66^. The complex docking models between TGFβRI/pY182-TGFβRI and TGFβRII were built with ClusPro web-based server (http://cluspro.bu.edu/)^67^. The residues that were used as attraction restraint included T185, T186, and S187 of TGFβRI, and AMPPNP601 of TGFβRII was retained. The top ten low-energy docked models from each restraint were downloaded from the server and then visualized and analyzed with PyMOL. Two final models were selected on the basis of substrate recognition and phosphotransfer mechanism from ATP hydrolysis to TGFβRI.

Atomic-resolution structures of the extracellular domains of the TGFβRI/TGF-β1/TGFβRII complex (PDB ID: 3KFD) and the extracellular domains of integrin αVβ1 (PDB ID: 3VI3) were downloaded from Protein Data Bank. Structures were uploaded, and molecular docking between the two complexes was then performed with the ClusPro web server (http://cluspro.bu.edu/)^67^. All the cartoon structural presentations in this manuscript were generated and displayed with PyMOL.

### Animal experiments

All animal experiments were carried out in accordance with protocols (00001397-RN02) approved by the MD Anderson Institutional Animal Care and Use Committee. The study is compliant with all relevant ethical regulations regarding animal research. Athymic NCr nu/nu mice (strain: #002019) were obtained from Jackson Lab. The mice were exposed to a 12-h light/12-h dark cycle at 22–24 °C with 50–60% humidity, bred as specific pathogen-free mice, and given free access to food and water. The number of mice used in each experimental group was determined via power analysis or based on prior experience with metastatic animal models, and mice were grouped randomly for each experiment. All sample sizes were listed in the corresponding figure legend or figures. All mice used were the same age (8 weeks) and had similar body weights. Two different injection models were used for bone metastasis studies. (1) Intracardiac injection model A: 1 × 10^5^ cells of the 231–1566 sublines or EZH2-knockout MDA-MB-231 sublines were injected into the left ventricle in anesthetized female athymic NCr nu/nu mice. GSK126 was dissolved in 20% cyclodextrin (Captisol; CyDex Pharmaceuticals) and adjusted to a pH level of 4.0 to 4.5 with 1 N acetic acid following the instructions described by McCabe et al.^56^. GSK126 was administrated to the mice via intraperitoneal (i.p.) injection three times a week at a dose of 150 mg/kg after 5 days of injections. (2) Intracardiac injection model B: 1 × 10^5^ cells of the 231–1566 control (1566.ctrl) or 231–1566 EZH2 knockout subclone mixture (1566.KO) were injected into the left ventricle in anesthetized female athymic NCr nu/nu mice. The first mouse group injected with 1566.ctrl were treated with EPZ-6438. EPZ-6438 was dissolved in 0.5% NaCMC + 0.1% Tween 80 in water and administrated to the mice via oral gavage, two times per day at a dose of 250 mg/kg after 6 days of injections. The second mouse group injected with 1566.ctrl and the third mouse group injected with 1566.KO were treated with vehicle as the same schedule as the first group. (3) Intratibial injection model A: 2 × 10^5^ MDA-MB-231 cells or 231.KO.mixed cells were injected into a tibia in anesthetized female athymic NCr nu/nu mice. (4) Intratibial injection model B: 2 × 10^5^ MDA-MB-231 cells were injected into a tibia in anesthetized female athymic NCr nu/nu mice. VS-6063 was prepared in a vehicle (0.5% hydroxypropyl methylcellulose with 0.1% Tween 80) and administered to the mice via oral gavage (50 mg/kg) twice a day after 18 days of injection, and GSK126 was administered to them via i.p. injection every day at a dose of 100 mg/kg after 18 days of injections. Development of bone metastases and tumor burdens were monitored using bioluminescence imaging (BLI), and endpoints includes weight loss, loss of mobility, and other signs of distress. After anesthetized mice were intraperitoneally injected with 75 mg/kg D-luciferin, BLI was performed using a Xenogen IVIS 200 imaging system (PerkinElmer). Analysis of bone metastasis was performed using living image software by measuring the photon flux in the hindlimbs of mice. The photon flux curves were normalized according to the signal on the day when mice were given the drug GSK126 or vehicle. Bone metastasis-free survival curves showed the time point at which each mouse experienced bone metastasis development according to threshold BLI signals in the hindlimbs. X-ray images of hindlimbs of mice were obtained using an IVIS Lumina XR system (PerkinElmer).

### Immunohistochemistry staining and scoring system

Standard immunohistochemistry (IHC) staining was performed as described previously^68^. The immunoreactive score (IRS) was used to quantify the IHC staining, ranging from 0 to 12 as a result of multiplication of positive cell proportion scores (0–4) and staining intensity scores (0–3). IHC staining and statistical analysis results were independently evaluated by two pathologists blinded to the experimental groups.

### Statistics and reproducibility

All quantitative experiments were performed using at least three independent biological repeats, and the results are presented as means ± standard deviation (S.D.) or means ± standard error of the mean (S.E.M.). One-way analysis of variance (multiple groups) or *t*-tests (two groups) were used to compare the means for two or more samples using the Prism 8 software program (GraphPad Software). Survival was analyzed using Kaplan–Meier curves and log-rank tests. *P*-values < 0.05 (two-sided) were considered statistically significant. For IHC score, ten visual fields from different areas of each tumor were evaluated by two pathologists independently (blinded to experiment groups). For migration, invasion, and TRAP + osteoclasts staining experiments, more than three visual fields were evaluated, and three independent biology repeats Fwere performed. Representative images of micrographs were shown to represent reproducible data from various experiments using micrographs.

### Reporting summary

Further information on research design is available in the Nature Research Reporting Summary linked to this article.

## Supplementary information

Supplementary Information

Description of Additional Supplementary Information

Supplementary Data1

Supplementary Data 2

Reporting Summary

## Data Availability

Authors can confirm that all relevant data are included in the paper and/or its Supplementary Information files. Source data are provided with this paper. The ChIP-seq data used in this study are available in database GSE188640. Patient dataset used in this study are available in database GSE14020, and GSE2603. PDB ID code 1ias was used for TBFβRI structure; PDB ID code 5e92 was used for TGFβRII structure; PDB ID 3KFD was used for TGFβRI/TGF-β1/TGFβRII complex structure; PDB ID 3VI3 was used for integrin αVβ1 structure. Source data are provided with this paper.

## Source data

Source Data
