# Supplementary material for: EZH2 engages TGFβ signaling to promote breast cancer bone metastasis via integrin β1-FAK activation
Source: Nat Commun. 2022 May 10;13:2543. doi: 10.1038/s41467-022-30105-0 (PMC9091212; doi:10.1038/s41467-022-30105-0)
Supplement: Supplementary file 1 — Supplementary Information [file 41467_2022_30105_MOESM1_ESM.pdf]

Supplementary Data

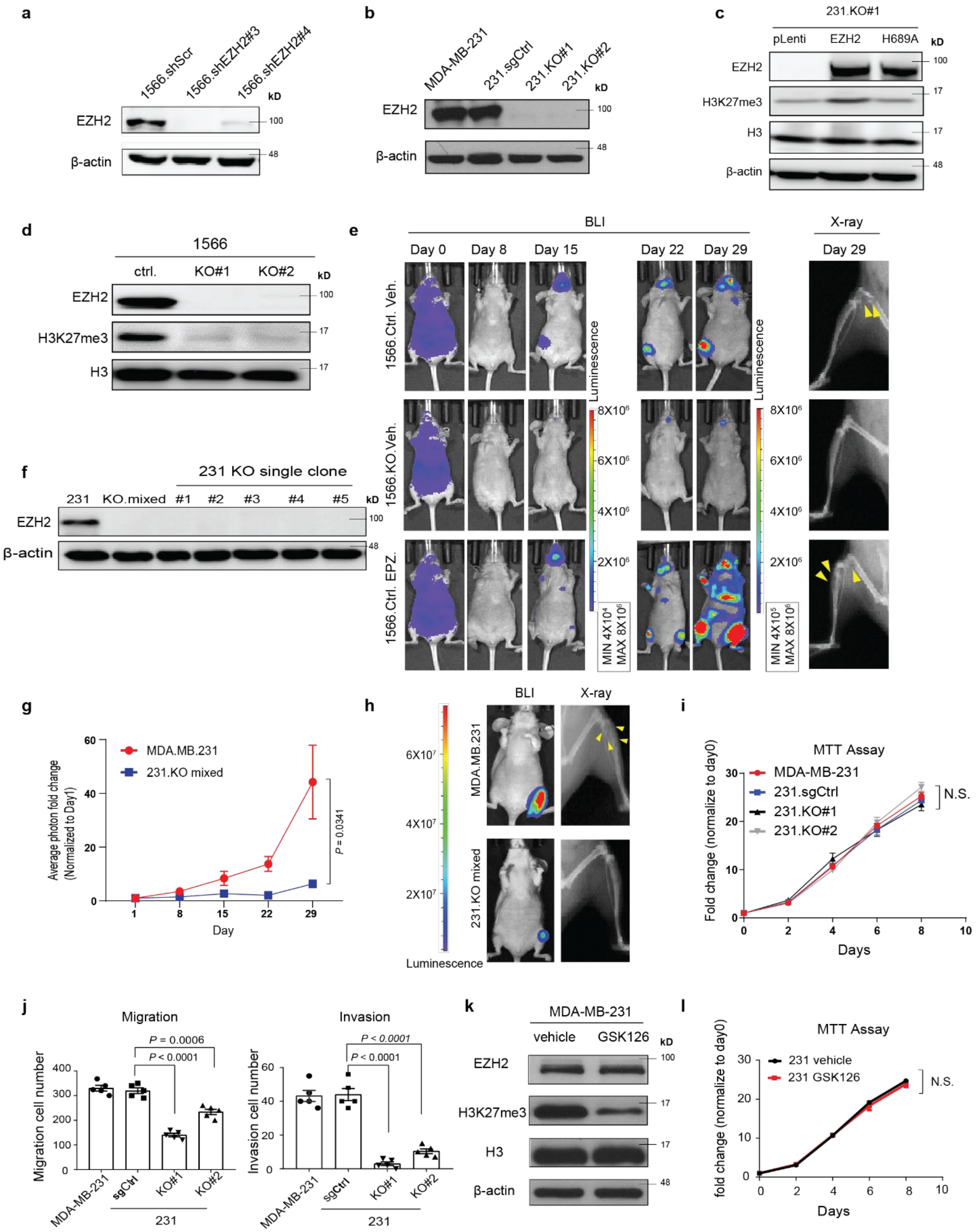

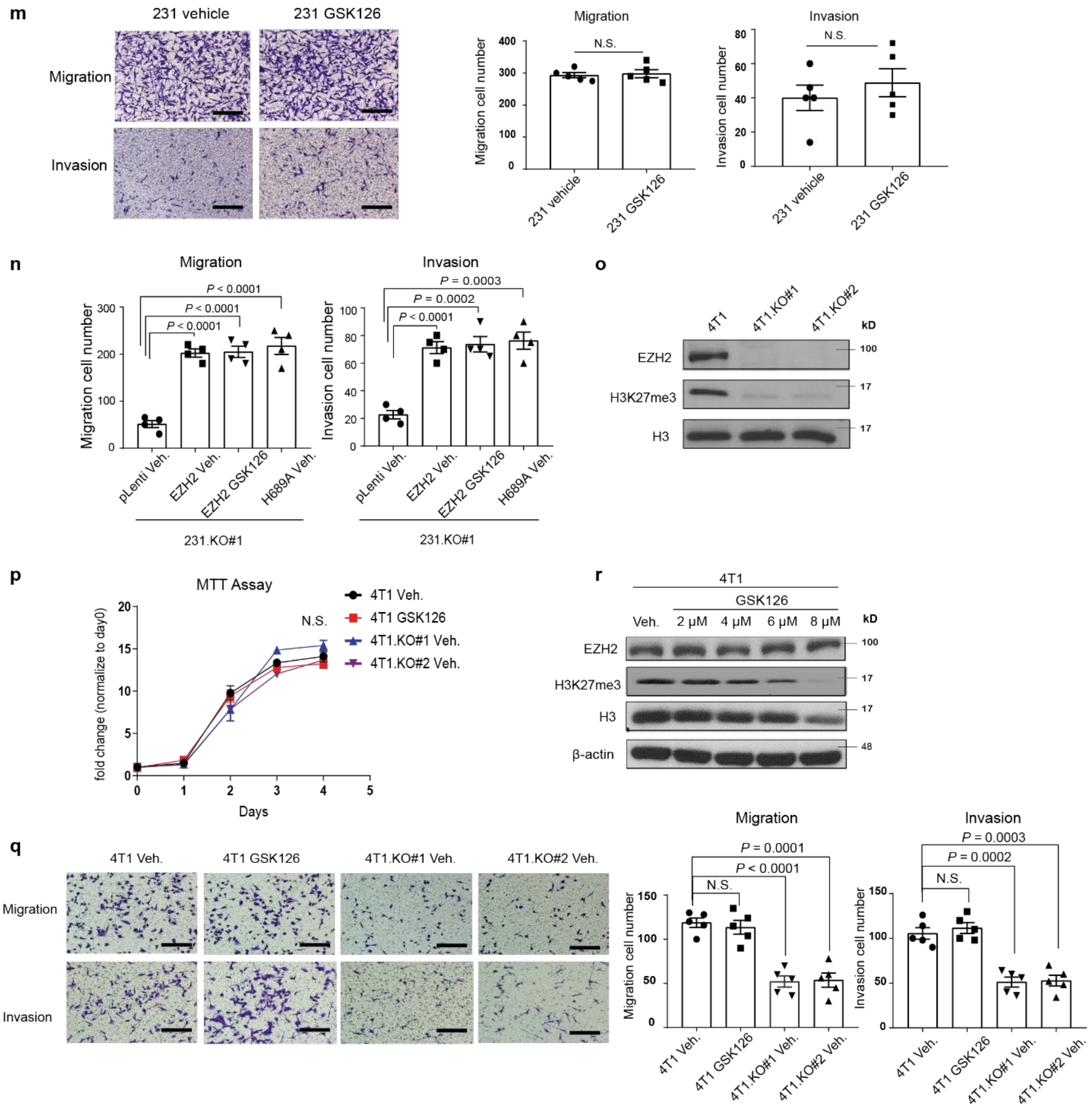

Supplementary Fig. 1. EZH2 promotes migration and invasion of breast cance cells. **a,** Western blotting of the expression of EZH2 and β-actin in 1566.shScr, 1566.shEZH2#3, and 1566.shEZH2#4 cells. **b,** Western blotting of the expression of EZH2 and β-actin in MDA-MB-231, 231.sgCtrl., 231.KO#1, and 231.KO#2 cells. **c,** Western blotting of the expression of EZH2, H3K27me3, H3, and β-actin in 231.KO#1 cell sublines with re-expression of a control pLenti vector (231.KO#1.pLenti), wild-type EZH2 (231.KO#1.EZH2), or EZH2 with the H689A

mutant (231.KO#1.H689A). **d,** Western blotting of the expression of EZH2, H3K27me3 and H3 in 1566.Ctrl and two 1566 EZH2 knockout sublines (KO#1, KO#2). 1566.KO cells are mixture of these two KO sublines. **e,** Representative bioluminescence (BLI), and X-ray of bone-metastatic lesions in the 3 subgroups described in **Fig**. **1e, f** obtained at the indicated time points. The arrows indicate osteolytic bone lesions in X-ray images. The arrows indicate osteolytic bone lesions in X-ray images. **f,** Western blotting of EZH2 and β-actin in indicated cells. **g**. Quantification of the fold change of BLI intensities in the right leg region of mice in two groups intratibially injected with MDA-MB-231 (n = 6) versus 231.KO mixed cells (n = 5). The BLI signal was normalized with the signal on the first day after injection. Data are presented as means ± S.E.M. *t*-test (two-sided). **h,** Representative BLI and X-ray images of bone-metastatic lesions in the two groups described in **g.** The arrows indicate osteolytic bone lesions in X-ray images. **i,** MTT assay results showing proliferation of MDA-MB-231, 231.sgCtrl., 231.KO#1, and 231.KO#2 cells. Four biologically independent experiments. Data are presented as means ± S.D. *t*-test (two-sided). N.S., not significant. **j,** Quantification of invading and migrating MDA-MB-231, 231.sgCtrl., 231.KO#1, and 231.KO#2 cells. Data are presented as means ± S.E.M. *t*-test. (two sided). Five biologically independent experiments. **k,** Western blotting of the expression of EZH2, H3K27me3, H3, and β-actin in MDA-MB-231 cells treated with vehicle (DMSO) or GSK126 (2 μM, 24 hours). **l,** MTT assay results showing proliferation of MDA-MB-231 cells treated with vehicle or GSK126 (2 μM, 24 hours). Four biologically independent experiments. Data are presented as means ± S.D. *t*-test. N.S., not significant (two-sided). **m,** Representative images and quantification of invading and migrating MDA-MB-231 cells treated with vehicle or GSK126 (2 μM, 24 hours). Scale bars, 100 μm. Four biologically independent experiments. Data are presented as means ± S.E.M. *t*-test (two-sided). **n,** Quantification of

invading and migrating 231.KO#1.pLenti. cells treated with vehicle, 231.KO#1.EZH2 cells treated with vehicle, 231.KO#1.EZH2 cells treated with 2 μM GSK126, and 231.KO#1.H689A cells treated with vehicle. Four biologically independent experiments. Data are presented as means ± S.E.M. *t*-test (two-sided). **o,** Western blotting of the expression of EZH2, H3K27me3, and H3 in 4T1, 4T1.KO#1, and 4T1.KO#2 cells. **p,** MTT assay results showing proliferation of 4T1 cells treated with vehicle or with GSK126 (6 μM), 4T1.KO#1 cells, and 4T1.KO2#2 cells treated with vehicle. Four biologically independent experiments. Data are presented as means ± S.D. *t*-test (two-sided). N.S., not significant. **q,** Representative images and quantification of invading and migrating 4T1 cells treated with vehicle or GSK126 (6 μM), 4T1.KO#1 cells, and 4T1.KO#2 cells treated with vehicle. Scale bars, 100 μm. Data are presented as means ± S.E.M. *t*-test (two-sided). Five biologically independent experiments. **r,** Western blotting of the expression of EZH2, H3K27me3, H3, and β-actin in 4T1 cells treated with vehicle or GSK126 at different concentrations (2-8 μM, 48 hours). All *P* values are indicated in the figure.

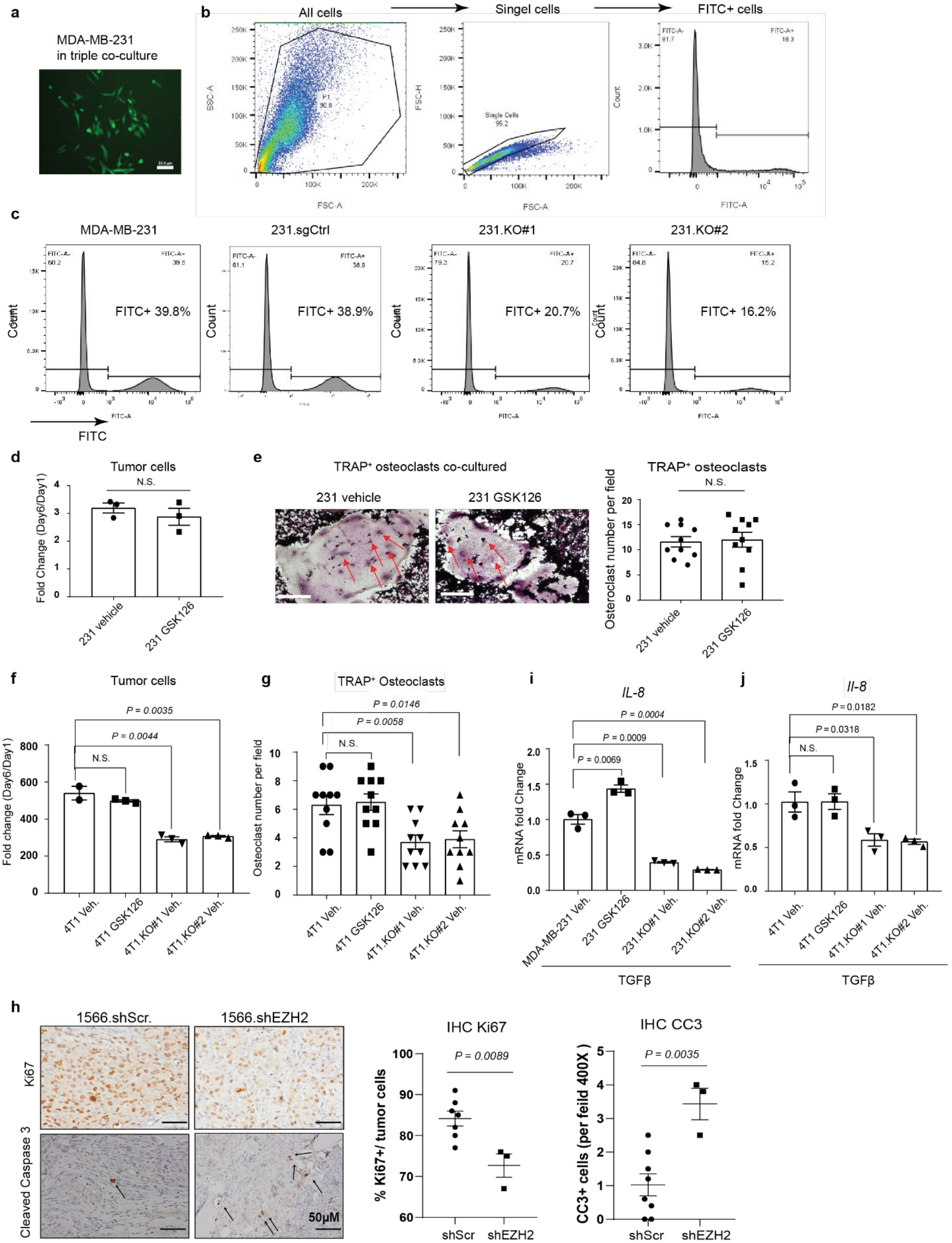
a
MDA-MB-231
in triple co-culture
b
All cells
Singel cells
FITC+ cells
c
MDA-MB-231
231.sgCtrl
231.KO#1
231.KO#2
FITC+ 39.8%
FITC+ 38.9%
FITC+ 20.7%
FITC+ 16.2%
Count
FITC
d
Tumor cells
N.S.
Fold Change (Day6/Day1)
231 vehicle
231 GSK126
e
TRAP+ osteoclasts co-cultured
231 vehicle
231 GSK126
TRAP+ osteoclasts
N.S.
Osteroclast number per field
f
Tumor cells
P = 0.0035
P = 0.0044
N.S.
Fold change (Day6/Day1)
4T1 Veh.
4T1 GSK126
4T1.KO#1 Veh.
4T1.KO#2 Veh.
g
TRAP+ Osteoclasts
P = 0.0146
P = 0.0058
N.S.
Osteoclast number per field
i
IL-8
P = 0.0004
P = 0.0009
P = 0.0069
mRNA fold Change
MDA-MB-231 Veh.
231 GSK126
231.KO#1 Veh.
231.KO#2 Veh.
TGFβ
j
Il-8
P = 0.0182
P = 0.0318
N.S.
mRNA fold Change
TGFβ
h
1566.shScr.
1566.shEZH2
Ki67
Cleaved Caspase 3
50μM
IHC Ki67
P = 0.0089
% Ki67+/ tumor cells
shScr
shEZH2
IHC CC3
P = 0.0035
CC3+ cells (per feild 400X)

Supplementary Fig. 2. EZH2 enhances tumor cell proliferation and osteoclast maturation in the triple co-culture system. **a,** Fluorescence image of MDA-MB-231 cells with expression of GFP in triple co-culture. Scale bars, 50 μm. **b,** Gating strategy for isolating GFP+ cells by flow cytometry. **c,** Flow cytometric analysis of GFP^+ breast cancer cells isolated from triple co-culture as measured in the fluorescein isothiocyanate (FITC) channel. **d,** Quantification of MDA-MB-231 cells treated with vehicle or GSK126 (2 μM) after co-culture with osteoclasts and MC3T3 osteoblasts and treated with TGFβ (5 ng/mL) for 6 days. Three biologically independent experiments. Data are presented as means ± S.E.M. *t*-test (two-sided). N.S., not significant. **e,** Representative staining images and quantification of mature $TRAP^+$ osteoclasts after co-culture with MC3T3 osteoblasts and MDA-MB-231 cells treated with vehicle or GSK126 (2 μM), and TGFβ (5 ng/mL) for 6 days. The arrows indicate multinuclear mature $TRAP^+$ osteoclasts. Ten random vision fields examined over three biologically independent experiments. Scale bars, 200 μm. Data are presented as means ± S.E.M. *t*-test (two-sided). **f,** Quantification of 4T1 cells treated with vehicle, or with GSK126 (6 μM), 4T1.KO#1 cells, and 4T1.KO#2 cells treated with vehicle, after co-culture with osteoclasts and MC3T3 osteoblasts and treated with TGFβ (5 ng/mL) for 6 days. Three biologically independent experiments. Data are presented as means ± S.E.M. *t*-test (two-sided). **g,** Quantification of mature $TRAP^+$ osteoclasts after co-culture with MC3T3 osteoblasts and the indicated cancer cells treated with TGFβ (5 ng/mL) for 6 days. Ten random vision fields examined over three biologically independent experiments. Data are presented as means ± S.E.M. *t*-test (two-sided). **h,** Representative pictures and quantification of IHC staining of Ki67 and Cleaved caspase 3 expression in the bone metastasis samples from the two subgroups of mice in **Fig. 1a, b.** The arrows indicate positive cleaved caspase 3 positive cells. $n = 7$ tissue slices examined in 1566.shScr subgroup; $n = 3$ tissue slices examined in 1566.shEZH2 subgroup. Data are presented as means ±

S.E.M. *t*-test (two-sided). **i,** qRT-PCR analysis of *IL-8* mRNA expression in the indicated cells treated with TGFβ (5 ng/mL, 2 hours). Three biologically independent experiments. Data are presented as means ± S.E.M. *t*-test (two-sided). **j,** qRT-PCR analysis of *Il-8* mRNA expression in the indicated cells treated with TGFβ (5 ng/mL, 2 hours). Three biologically independent experiments. Data are presented as means ± S.E.M. *t*-test (two-sided). All *P* values are indicated in the figure.

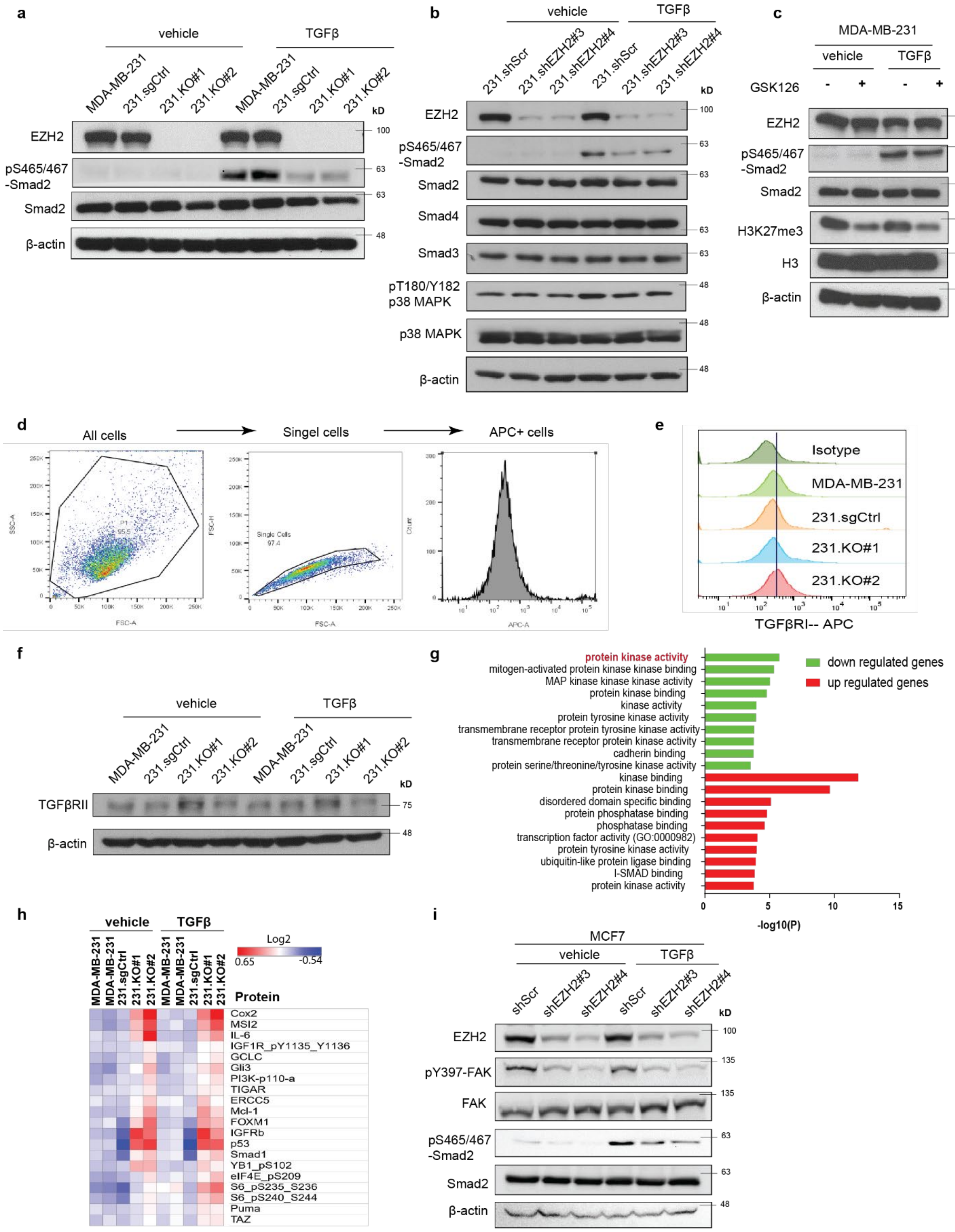
a
vehicle
TGFβ
MDA-MB-231
231.sgCtrl
231.KO#1
231.KO#2
MDA-MB-231
231.sgCtrl
231.KO#1
231.KO#2
kD
EZH2
100
pS465/467
-Smad2
63
Smad2
63
β-actin
48
b
vehicle
TGFβ
231.shScr
231.shEZH2#3
231.shEZH2#4
231.shScr
231.shEZH2#3
231.shEZH2#4
kD
EZH2
100
pS465/467
-Smad2
63
Smad2
63
Smad4
63
Smad3
63
pT180/Y182
p38 MAPK
48
p38 MAPK
48
β-actin
48
c
MDA-MB-231
vehicle
TGFβ
GSK126
-
+
-
+
kD
EZH2
100
pS465/467
-Smad2
63
Smad2
63
H3K27me3
17
H3
17
β-actin
48
d
All cells
Singel cells
APC+ cells
SSC-A
FSC-A
FSC-H
Single Cells
97.4
Count
APC-A
e
Isotype
MDA-MB-231
231.sgCtrl
231.KO#1
231.KO#2
TGFβRI-- APC
f
vehicle
TGFβ
MDA-MB-231
231.sgCtrl
231.KO#1
231.KO#2
MDA-MB-231
231.sgCtrl
231.KO#1
231.KO#2
kD
TGFβRII
75
β-actin
48
g
protein kinase activity
mitogen-activated protein kinase kinase binding
MAP kinase kinase kinase activity
protein kinase binding
kinase activity
protein tyrosine kinase activity
transmembrane receptor protein tyrosine kinase activity
transmembrane receptor protein kinase activity
cadherin binding
protein serine/threonine/tyrosine kinase activity
kinase binding
protein kinase binding
disordered domain specific binding
protein phosphatase binding
phosphatase binding
transcription factor activity (GO:0000982)
protein tyrosine kinase activity
ubiquitin-like protein ligase binding
I-SMAD binding
protein kinase activity
down regulated genes
up regulated genes
0
5
10
15
-log10(P)
h
vehicle
TGFβ
MDA-MB-231
MDA-MB-231
231.sgCtrl
231.KO#1
231.KO#2
MDA-MB-231
MDA-MB-231
231.sgCtrl
231.KO#1
231.KO#2
Log2
0.65
-0.54
Protein
Cox2
MSI2
IL-6
IGF1R_pY1135_Y1136
GCLC
Gli3
PI3K-p110-a
TIGAR
ERCC5
Mcl-1
FOXM1
IGFRb
p53
Smad1
YB1_pS102
eIF4E_pS209
S6_pS235_S236
S6_pS240_S244
Puma
TAZ
i
MCF7
vehicle
TGFβ
shScr
shEZH2#3
shEZH2#4
shScr
shEZH2#3
shEZH2#4
kD
EZH2
100
pY397-FAK
135
FAK
135
pS465/467
-Smad2
63
Smad2
63
β-actin
48

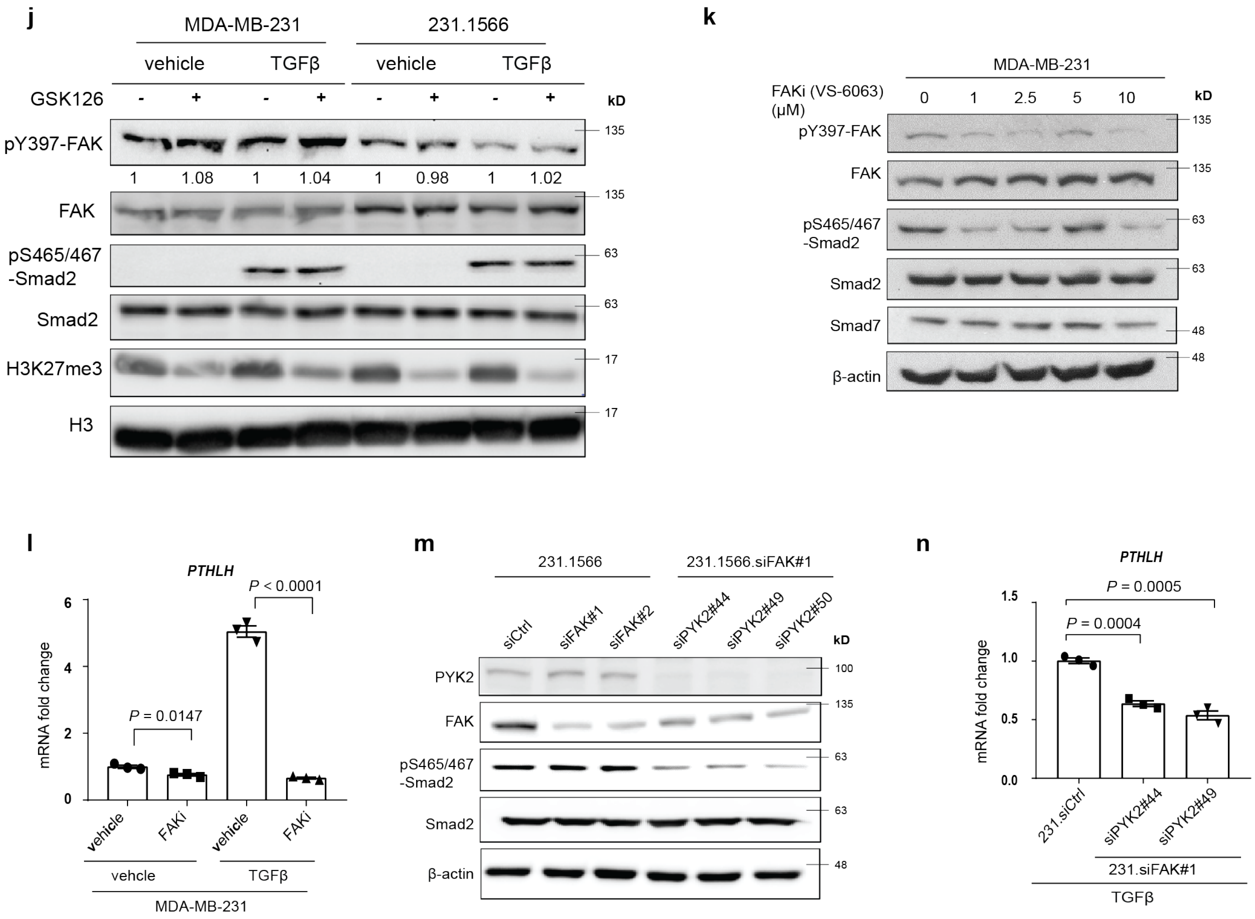

Supplementary Fig. 3. EZH2 increased pY397-FAK level, which enhances pS465/467-Smad2 expression of cancer cells in response to TGFβ stimulation. **a,** Western blotting of the expression of the indicated proteins in MDA-MB-231, 231.sgCtrl, 231.KO#1, and 231.KO#2 cells treated with vehicle or TGFβ (5 ng/mL) for 2 hours. **b,** Western blotting of the expression of the indicated proteins in 231.shScr, 231.shEZH2#3, and 231.shEZH2#4 cells treated with a vehicle or TGFβ (5 ng/mL) for 2 hours. **c,** Western blotting of the expression of the indicated proteins in MDA-MB-231 cells treated with vehicle or GSK126 (2 μM, 24 hours) and then vehicle or TGFβ (5 ng/mL, 2 hours). **d,** Gating strategy for measuring TGFβR1 by flow cytometry in the APC channel. **e,** Results of flow cytometric analysis of TGFβRI in the indicated cancer cells as measured in the APC channel. **f,** Western blotting of the expression of TGFβRII and β-actin in the indicated cells

treated with vehicle or TGFβ (5 ng/mL, 2 hours). **g,** Gene Ontology (GO) molecular functional analysis of RPPA data showing the function of upregulated and downregulated proteins in 231.KO#1 and 231.KO#2 cells compared with MDA-MB-231 and 231.sgCtrl cells. **h,** Results of RPPA analysis of MDA-MB-231, 231.sgCtrl, 231.KO#1, and 231.KO#2 cells treated with vehicle or TGFβ (5 ng/mL, 2 hours). The heat map shows the top upregulated proteins in 231.KO#1 and 231.KO#2 cells compared with MDA-MB-231 and 231.sgCtrl cells. **i,** Western blot of the expression of the indicated proteins in MCF7.shScr, MCF7.shEZH2#3, and MCF7.shEZH2#4 cells treated with vehicle or TGFβ (5 ng/mL, 2 hours). **j,** Western blotting of the expression of the indicated proteins in MDA-MB-231 or 231-1566 cells treated with a vehicle or GSK (2 μM, 24 hours) and then treated without or with TGFβ (5 ng/mL, 2 hours). **k,** Western blotting of the expression of the indicated proteins in MDA-MB-231 cells treated with the FAKi VS-6036 at different concentrations (0-10 μM) and with TGFβ (5 ng/mL, 2 hours). **l,** Results of qRT-PCR analysis of *PTHLH* mRNA expression in MDA-MB-231 cells treated with vehicle or VS-6036 (10 μM, 24 hours) and then vehicle or TGFβ (5 ng/mL, 2 hours). Three biologically independent experiments. Data are presented as means ± S.E.M. *t*-test (two-sided). **m,** Western blotting of the expression of PYK2, FAK, pS465/467-Smad2, Smad2, and β-actin in the indicated 231-1566 cell sublines treated with TGFβ (5 ng/mL, 2 hours). **n,** Results of qRT-PCR analysis of *PTHLH* mRNA expression in 231.siCtrl, 231.siFAK#1 siPYK2#44, and 231.siFAK#1 siPYK2#49 cells treated with TGFβ (5 ng/mL, 2 hours). Three biologically independent experiments. Data are presented as means ± S.E.M. *t*-test (two-sided).

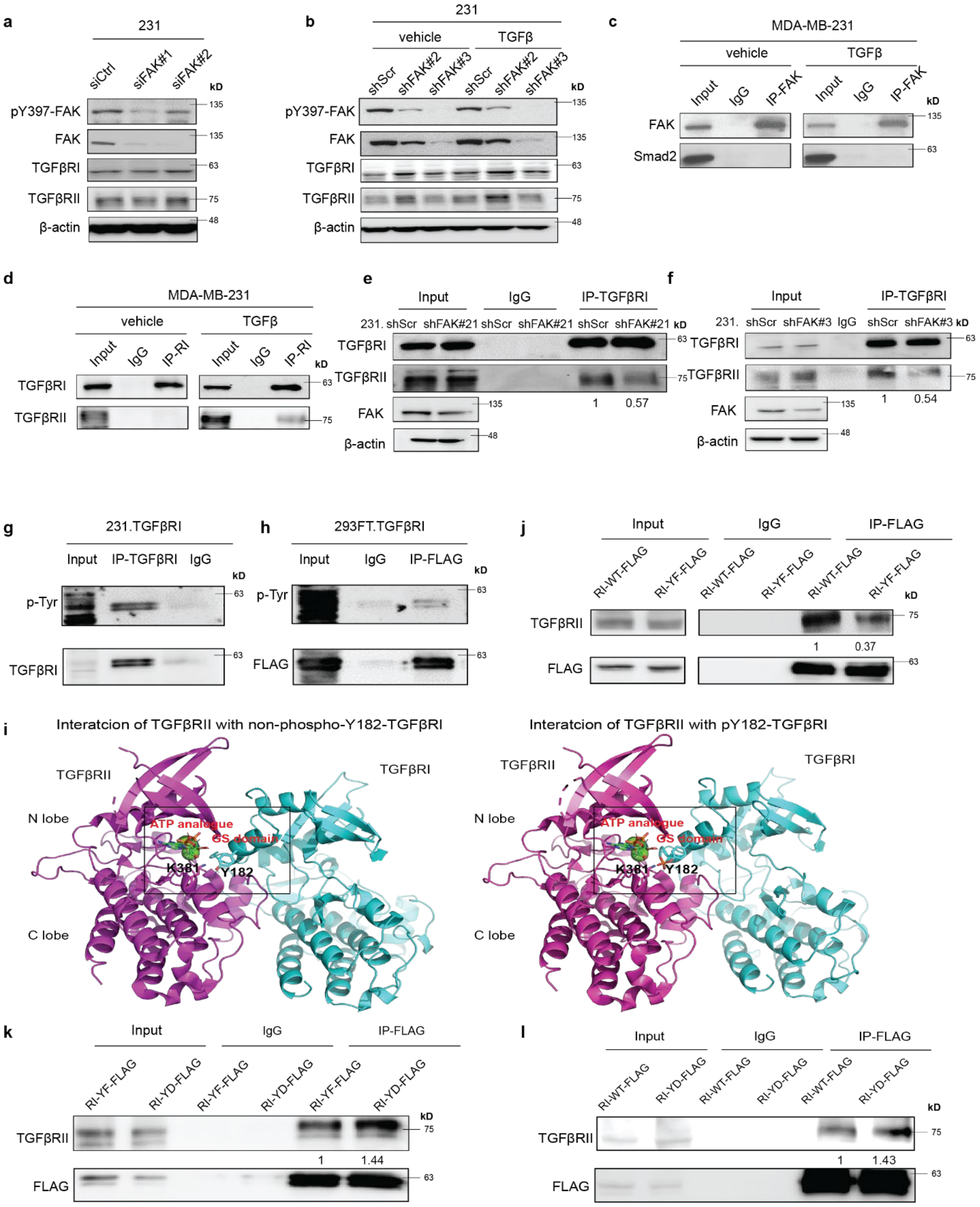

Supplementary Fig. 4. FAK inhibitor inhibits TGFβRI binding to TGFβRII. **a,** Western blotting of the expression of the indicated proteins in 231.siCtrl, 231.siFAK#1, and 231.siFAK#2 cells. **b,**

Western blotting of the expression of the indicated proteins in 231.shCtrl, 231.shFAK#2, and 231.siFAK#3 cells treated with vehicle or TGFβ (5 ng/mL, 2 hours). **c,** IP of FAK from the lysis of MDA-MB-231 cells treated with vehicle or TGFβ (5 ng/mL, 2 hours) followed by Western blotting for FAK and Smad2. **d,** IP of TGFβRI from the lysis of MDA-MB-231 cells treated with vehicle or TGFβ (5 ng/mL, 2 hours) followed by Western blotting for TGFβRI and TGFβRII. **e,** IP of TGFβRI from 231.shScr, and 231.shFAK#21 cell lysis followed by Western blotting for TGFβRI and TGFβRII. Western blotting detected FAK and β-actin in the inputs of 231.shScr and 231.shFAK#21 cells. **f,** IP of TGFβRI from 231.shScr and 231.shFAK#3 cell lysis followed by Western blotting for TGFβRI and TGFβRII. Western blotting detected FAK and β-actin in the inputs of 231.shScr and 231.shFAK#3 cells. **g**, MDA-MB-231 cells were transfected with FLAG tagged TGFβRI plasmid (231.TGFβRI). IP of FLAG from the lysis of 231.TGFβRI cells using anti-FLAG antibody, followed by Western blotting for phospho-Tyrosine. **h,** HEK293FT cells were transfected with FLAG tagged TGFβRI plasmid (293FT.TGFβRI). IP of FLAG from the lysis of 293FT.TGFβRI cells using anti-FLAG antibody, followed by Western blotting for phospho-Tyrosine. **i,** Docked complex models showing the interaction between GS domain of TGFβRI (without/with phosphorylation of Y182) and catalytic site of TGFβRII. **j,** HEK 293FT cells were transfected with TGFβRII, and FLAG tagged wild type TGFβRI (RI-WT-FLAG) or TGFβRI-Y182F mutant (RI-YF-FLAG). IP of FLAG from the lysis of cells using anti-FLAG antibody, followed by Western blotting for TGFβRII. **k,** HEK293FT cells were transfected with TGFβRII and FLAG tagged TGFβRI-Y182F mutant (RI-YF-FLAG) or TGFβRI-Y182D mutant (RI-YD-FLAG). IP of FLAG from the lysis of cells using anti-FLAG antibody, followed by Western blotting for TGFβRII. **l,** HEK 293FT cells were transfected with TGFβRII, and FLAG-

tagged wild type TGFβRI (RI-WT-FLAG) or TGFβRI-Y182D mutant (RI-YD-FLAG). IP of FLAG from cell lysates using anti-FLAG antibody was followed by Western blotting for TGFβRII.

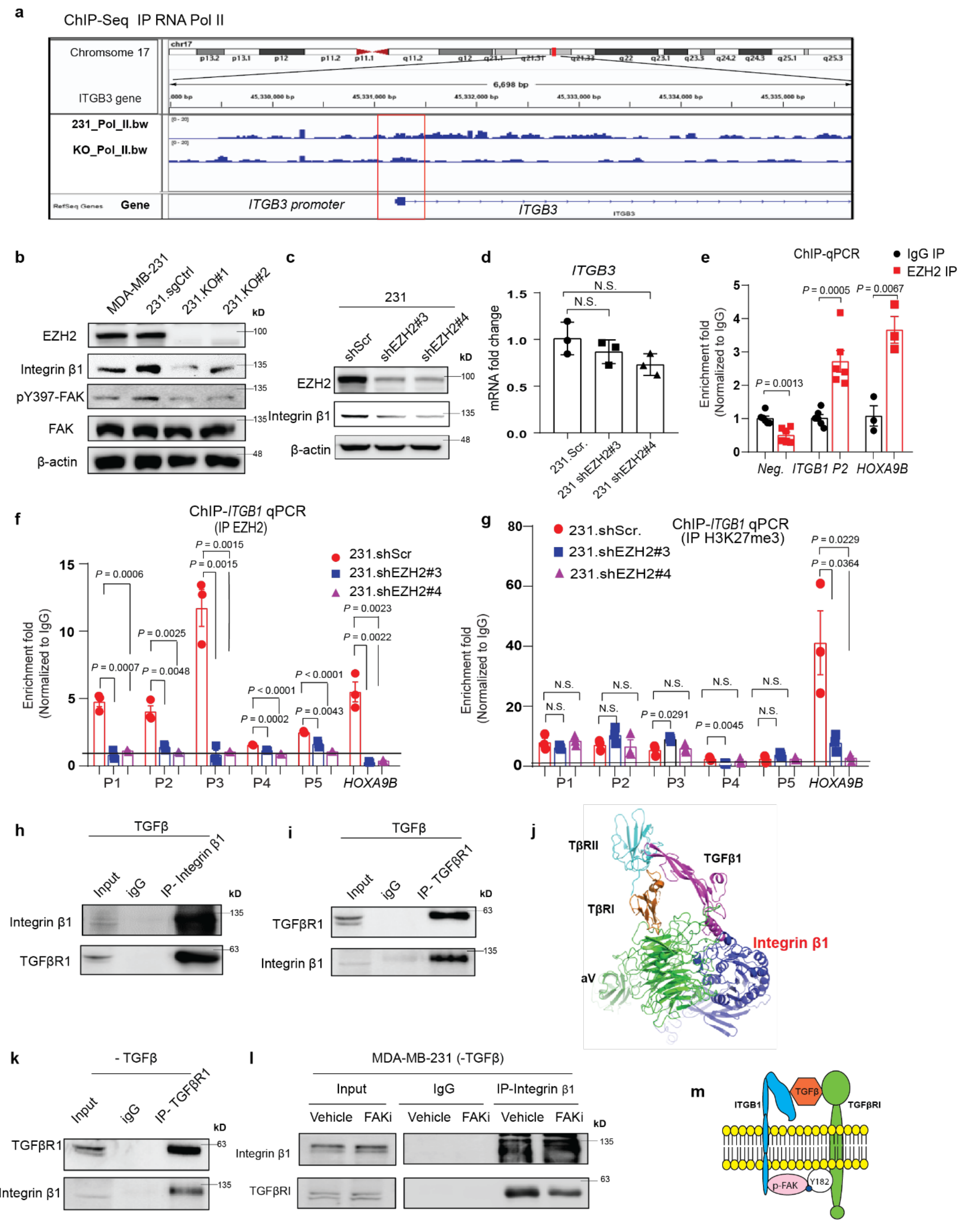
a
ChIP-Seq IP RNA Pol II
Chromsome 17
ITGB3 gene
231_Pol_II.bw
KO_Pol_II.bw
Gene
ITGB3 promoter
ITGB3
b
MDA-MB-231
231.sgCtrl
231.KO#1
231.KO#2
EZH2
Integrin β1
pY397-FAK
FAK
β-actin
c
231
shScr
shEZH2#3
shEZH2#4
EZH2
Integrin β1
β-actin
d
ITGB3
mRNA fold change
N.S.
231.Scr.
231 shEZH2#3
231 shEZH2#4
e
ChIP-qPCR
IgG IP
EZH2 IP
Enrichment fold (Normalized to IgG)
P = 0.0013
P = 0.0005
P = 0.0067
Neg.
ITGB1 P2
HOXA9B
f
ChIP-ITGB1 qPCR (IP EZH2)
231.shScr
231.shEZH2#3
231.shEZH2#4
Enrichment fold (Normalized to IgG)
P = 0.0006
P = 0.0007
P = 0.0025
P = 0.0048
P = 0.0015
P = 0.0015
P < 0.0001
P = 0.0002
P < 0.0001
P = 0.0043
P = 0.0023
P = 0.0022
P1
P2
P3
P4
P5
HOXA9B
g
231.shScr.
231.shEZH2#3
231.shEZH2#4
ChIP-ITGB1 qPCR (IP H3K27me3)
Enrichment fold (Normalized to IgG)
N.S.
P = 0.0291
P = 0.0045
P = 0.0229
P = 0.0364
P1
P2
P3
P4
P5
HOXA9B
h
TGFβ
Input
igG
IP- Integrin β1
Integrin β1
TGFβR1
i
TGFβ
Input
igG
IP- TGFβR1
TGFβR1
Integrin β1
j
TβRII
TGFβ1
TβRI
Integrin β1
aV
k
- TGFβ
Input
igG
IP- TGFβR1
TGFβR1
Integrin β1
l
MDA-MB-231 (-TGFβ)
Input
IgG
IP-Integrin β1
Vehicle
FAKi
Integrin β1
TGFβRI
m
ITGB1
TGFβ
TGFβRI
p-FAK
Y182

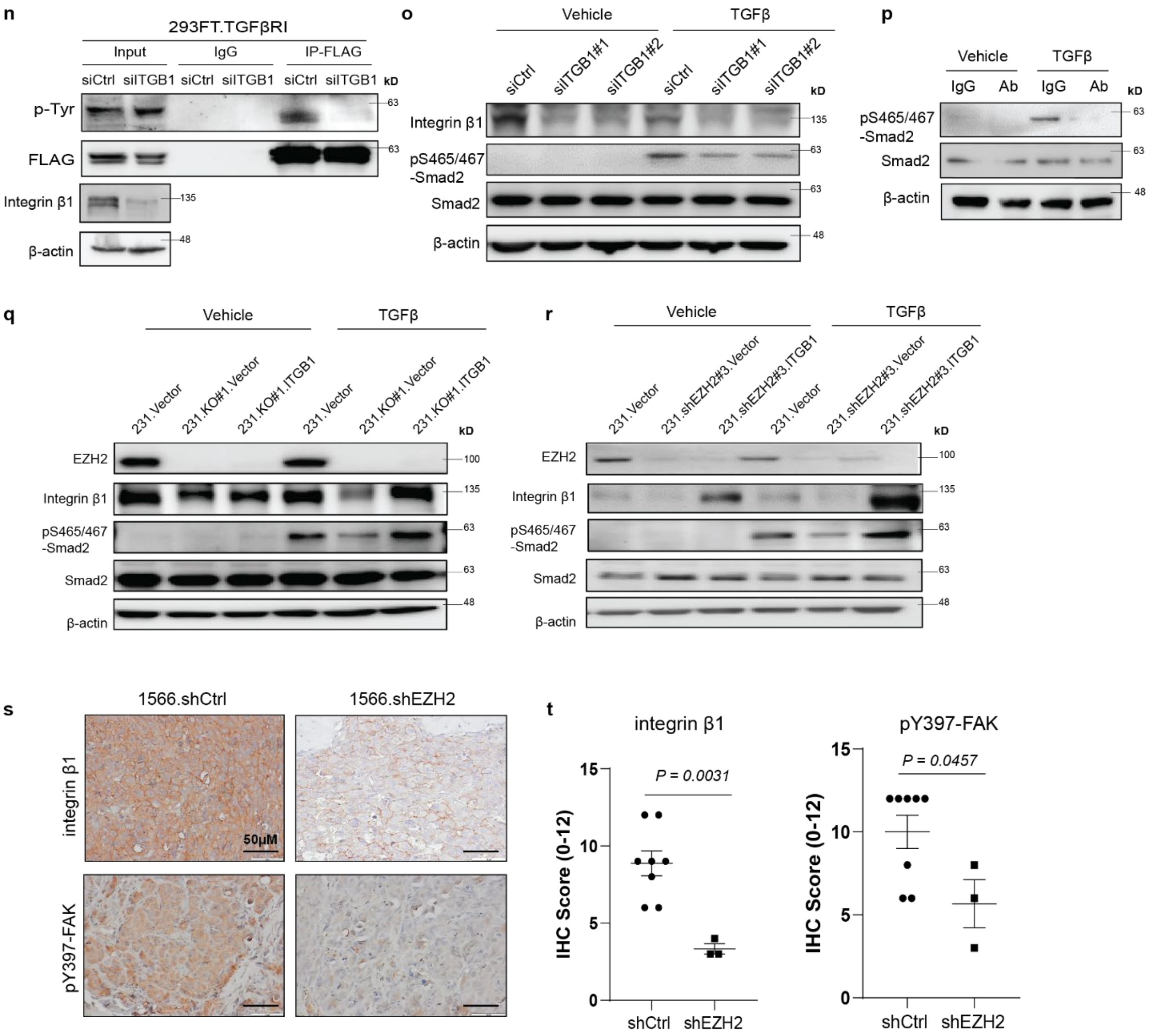

Supplementary Fig. 5. EZH2 increases *ITGB1* mRNA expression and binds at the promoter of *ITGB1* gene. **a,** Screenshot of the RNA Pol II ChIP sequencing (ChIP-seq) signal (GSE188640) at the *ITGB3 promoter* locus in MDA-MB-231 (231_Pol _II.bw) and 231.KO#1 (KO_Pol_II.bw) cells. **b,** Western blotting of the expression of the indicated proteins in MDA-MB-231, 231.sgCtrl, 231.KO#1, and 231.KO#2 cells. **c,** Western blotting of the expression of the indicated proteins in 231.shSrc, 231.shEZH2#3, and 231.shEZH2#4 cells. **d,** Results of qRT-PCR analysis of *ITGB3* mRNA expression in the indicated cells. Three biologically independent experiments. Data are presented as means ± S.E.M. *t*-test (two-sided). **e**, EZH2 was immunoprecipitated from MDA-

MB-231 cell lysates, and EZH2 binding to negative control (*Neg*., Src gene promoter), *ITGB1* promoter*, and HOXA9B* promoter were detected using qPCR with indicated primers. n = 6 experiments examined using *Neg*. and *ITGB1 P2* primers; n = 3 experiments examined using *HOXA9B* primer. Data are presented as means ± S.E.M. *t*-test (two-sided). **f,** EZH2 was immunoprecipitated from 231.shSrc, 231.shEZH2#3, and 231.shEZH2#4 cells, and EZH2 binding to *ITGB1* was detected using qPCR with the indicated primers. *HOXA9B* was used as a positive control, and all fold-enrichment values were normalized according to IgG values. Three biologically independent experiments. Data are presented as means ± S.E.M. *t*-test. **g,** H3K27me3 was immunoprecipitated from 231.shSrc, 231.shEZH2#3, and 231.shEZH2#4 cells, and H3K27me3 binding to *ITGB1* or *HOXA9B* was detected using qPCR with the indicated primers. *HOXA9B* was used as a positive control, and all fold-enrichment values were normalized according to IgG values. Three biologically independent experiments. Data are presented as means ± S.E.M. *t*-test (two-sided). **h,** Integrin β1 protein was immunoprecipitated from the lysis of MDA-MB-231 cell treated with TGFβ (5 ng/mL, 2 hours) by anti-integrin β1 antibodies, which was followed by Western blotting for TGFβRI. **i,** TGFβRI protein was immunoprecipitated from the lysis of MDA-MB-231 cell treated with TGFβ (5 ng/mL, 2 hours) by TGFβRI antibodies, which was followed by Western blotting for Integrin β1. **j,** Docking model depicting how TGFβRI could directly interact with integrin αVβ1 among their ectodomains through TGFβ1. **k,** TGFβRI protein was immunoprecipitated by TGFβRI antibodies from cell lysates of MDA-MB-231 cells without TGFβ treatment, then integrin β1 and TGFβRI were detected by Western blotting. **l,** After IP of Integrin β1 from MDA-MB-231 cell lysates treated with vehicle or the FAKi VS-4718 (1 μM) for 24 hours, integrin β1 and TGFβRI were detected by Western blotting. **m,** The cartoon showing the possible interactions between integrin β1 and TGFβRI. **n,** HEK 293FT cells expressing FLAG-tagged wild-

type TGFβRI, were transfected with siRNA targeting ITGB1 or control siRNA. IP were performed with IgG as a control or with anti-FLAG antibody to pull down FLAG-TGFβRI from cell lysates, followed by Western blotting of phospho-Tyrosine (p-Tyr) (left). Integrin β1 and β-actin expressions in HEK 293FT.TGFβRI cells transfected with siCtrl or siITGB1 were detected by Western blotting (right). **o,** Western blotting of the expression of the indicated proteins in 231.siCtrl, 231.siITGB1#1, and 231.siITGB1#2 cells treated with vehicle or TGFβ (5 ng/mL) for 2 hours. **p,** Western blotting of the indicated proteins in MDA-MB-231 treated with IgG or ITGB1 antibody (sc-9970, 5ug/ml, 8h) and followed with vehicle or TGFβ (5 ng/mL) treatment for 2 hours. **q,** Western blotting of the indicated proteins in 231.Vector, 231.KO#1.Vector, and 231.KO#1.ITGB1 cells treated with vehicle or TGFβ (5 ng/mL) for 2 hours. **r,** Western blotting of the indicated proteins in 231.Vector, 231.shEZH2#3.Vector, and 231.shEZH2#3.ITGB1 cells treated with vehicle or TGFβ (5 ng/mL) for 2 hours. **s-t,** Representative data of IHC staining of integrin β1 and pY397-FAK expression in the bone metastases from the two subgroups of mice in Fig. **1a-b**. n = 8 tissue slices examined in 1566.shScr subgroup; n = 3 tissue slices examined in 1566.shEZH2 subgroup. Data are presented as means ± S.E.M. t-test (two-sided). All P values are indicated in the figure.

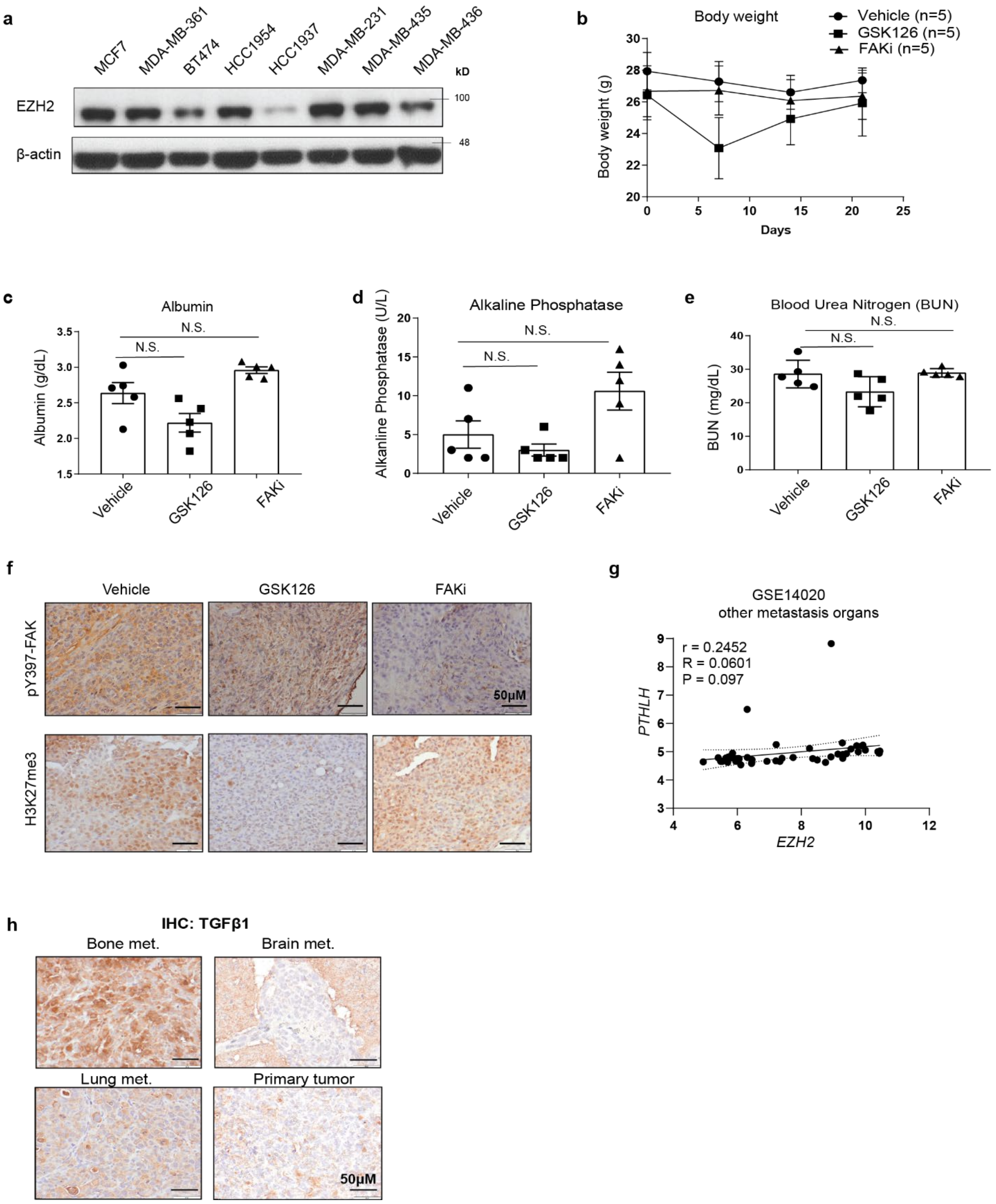

Supplementary Fig. 6. FAK inhibitor VS-6063 inhibits breast cancer bone metastasis without a discernable side effect. **a,** Western blotting of the expression of EZH2 and β-actin in the indicated

breast cancer cells. **b,** Body-weight curves for three subgroups of mice intratibially injected with MDA-MB-231 in cells and given treatment with vehicle, GSK126 (100 mg/kg/day, i.p. injection), or the FAKi VS-6063 (50 mg/kg, twice a day, oval gavage) beginning on day 18 after injection. n = 5 in each subgroup. Data are presented as means ± S.D. **c-e,** The albumin (**c**), alkaline phosphatase (**d**), and blood urea nitrogen (**e**) expression levels the blood of the three subgroups of mice in **c**. n = 5 in each subgroup. Data are presented as means ± S.E.M. *t*-test (two-sided). N.S., not significant. **f,** Representative pictures of IHC staining of pY397-FAK and H3K27me3 expression in the bone metastasis samples obtained from the three subgroups of mice in **b**. **g,** The Pearson *r* correlation for *EZH2* and *PTHLH* RNA mRNA expression in lung, liver, and brain metastases in breast cancer patients (GSE14020 data set). *P* value is indicated in the figure. **h,** IHC staining of TGFβ1 in MDA-MB-231 cell-induced bone metastasis, brain metastasis, lung metastasis and primary tumor growing in the mammary fat pad (MFP).
