# Supplementary material for: EZH2 engages TGFβ signaling to promote breast cancer bone metastasis via integrin β1-FAK activation
Source: Nat Commun. 2022 May 10;13:2543. doi: 10.1038/s41467-022-30105-0 (PMC9091212; doi:10.1038/s41467-022-30105-0)
Supplement: Supplementary file 2 — Description of Additional Supplementary Information [file 41467_2022_30105_MOESM2_ESM.pdf]

Descriptions of additional Supplementary Data Files

Supplementary Data 1: RPPA data. Legend: The up and down regulated proteins in the two EZH2-knockout cell lines compared to that in MDA-MB-231 and 231.sgCtrl cells.

Supplementary Data 2: Gene data from ChIP-seq. analysis. Legend: ChIP-seq analysis has identified 470 genes with decreased RNA Pol II binding in gene promoters in EZH2 KO and EZH2-Y696F mutant samples compared to EZH2 wild type samples.

Supplementary Data 3: FCAS gating strategy. Legend: FCAS gating strategy corresponds to Supplementary Figure 2b and 3d.
