# Supplementary material for: EZH2 engages TGFβ signaling to promote breast cancer bone metastasis via integrin β1-FAK activation
Source: Nat Commun. 2022 May 10;13:2543. doi: 10.1038/s41467-022-30105-0 (PMC9091212; doi:10.1038/s41467-022-30105-0)
Supplement: Supplementary file 5 — Reporting Summary [file 41467_2022_30105_MOESM5_ESM.pdf]

nature portfolio

Corresponding author(s): Dihua Yu

Last updated by author(s): 03/23/2022

Reporting Summary

Nature Portfolio wishes to improve the reproducibility of the work that we publish. This form provides structure for consistency and transparency in reporting. For further information on Nature Portfolio policies, see our Editorial Policies and the Editorial Policy Checklist.

Statistics

For all statistical analyses, confirm that the following items are present in the figure legend, table legend, main text, or Methods section.

n/a	Confirmed	
☐	☒	The exact sample size (*n*) for each experimental group/condition, given as a discrete number and unit of measurement
☐	☒	A statement on whether measurements were taken from distinct samples or whether the same sample was measured repeatedly
☐	☒	The statistical test(s) used AND whether they are one- or two-sided<br>*Only common tests should be described solely by name; describe more complex techniques in the Methods section.*
☐	☒	A description of all covariates tested
☐	☒	A description of any assumptions or corrections, such as tests of normality and adjustment for multiple comparisons
☐	☒	A full description of the statistical parameters including central tendency (e.g. means) or other basic estimates (e.g. regression coefficient) AND variation (e.g. standard deviation) or associated estimates of uncertainty (e.g. confidence intervals)
☐	☒	For null hypothesis testing, the test statistic (e.g. *F*, *t*, *r*) with confidence intervals, effect sizes, degrees of freedom and *P* value noted<br>*Give P values as exact values whenever suitable.*
☒	☐	For Bayesian analysis, information on the choice of priors and Markov chain Monte Carlo settings
☒	☐	For hierarchical and complex designs, identification of the appropriate level for tests and full reporting of outcomes
☐	☒	Estimates of effect sizes (e.g. Cohen's *d*, Pearson's *r*), indicating how they were calculated

Our web collection on statistics for biologists contains articles on many of the points above.

Software and code

Policy information about availability of computer code

Data collection	Data was collected using standard software programs: Flow cytometry samples were analyzed using a BD FACSCanto II cytometer (BD Biosciences). BLI data was acquired using an IVIS Lumina XR system (Perkin Elmer).
Data analysis	RPPA slides were scanned on a flatbed scanner to produce 16-bit tiff images. Spots from tiff images were identified and the density was quantified by Array-Pro Analyzer software. Each dilution curve was fitted using a logistic model (“SuperCurve Fitting” developed at MD Anderson) and normalized according to median polish. The heatmap included was generated in Cluster 3.0 (http://bonsai.hgc.jp/~mdehoon/software/cluster/software.htm) as a hierarchical cluster using Pearson Correlation and a center metric. The resulting heatmap was visualized in Treeview (http://www.eisenlab.org/eisen/) and presented as a high resolution .bmp format. The raw data of mass spectrometry were processed using Thermo ScientificTM Proteome DiscovererTM software version 1.4, spectra were searched against the Uniprot-Homo sapiens database using the Mascot search engine v2.3.02 (Matrix Science) run on an in-house server. Search results were trimmed to a 1% FDR for strict and 5% for relaxed condition using Percolator. For the trypsin, up to two missed cleavages were allowed. MS tolerance was set 10 ppm; MS/MS tolerance 0.8 Da. Carbamidomethylation on cysteine residues was used as fixed modification; oxidation of methione as well as phosphorylation of serine, threonine and tyrosine was set as variable modifications. BLI data was analyzed using Living Image software v.2.50. The quantification of western blot signal was analyzed by ImageJ software. The flow cytometry data was analyzed by flowJ software. The statistic analysis was using GraphPad Prism 6. Structures were uploaded, and molecular docking between the two complexes was then performed with the ClusPro web server (http://cluspro.bu.edu/)68. All the catoon structural presentations in this manuscript were generated and displayed with PyMOL.

For manuscripts utilizing custom algorithms or software that are central to the research but not yet described in published literature, software must be made available to editors and reviewers. We strongly encourage code deposition in a community repository (e.g. GitHub). See the Nature Portfolio guidelines for submitting code & software for further information.

Data

Policy information about availability of data

All manuscripts must include a data availability statement. This statement should provide the following information, where applicable:

- Accession codes, unique identifiers, or web links for publicly available datasets
- A description of any restrictions on data availability
- For clinical datasets or third party data, please ensure that the statement adheres to our policy

Authors can confirm that all relevant data are included in the paper and/or its Supplementary Information files. Source data are provided with this paper. The ChIP-seq data used in this study are available in database GSE188640. PDB ID code 1ias was used for TBFβRI structure; PDB ID code 5e92 was used for TGFβRII structure; PDB ID: 3KFD was used for TGFβRI/TGF-β1/TGFβRII complex structure; PDB ID: 3VI3 was used for integrin αVβ1 structure.

Field-specific reporting

Please select the one below that is the best fit for your research. If you are not sure, read the appropriate sections before making your selection.

[x] Life sciences [ ] Behavioural & social sciences [ ] Ecological, evolutionary & environmental sciences

For a reference copy of the document with all sections, see nature.com/documents/nr-reporting-summary-flat.pdf

Life sciences study design

All studies must disclose on these points even when the disclosure is negative.

Sample size	The number of mice used in each experimental group was determined by power analysis (achieving 80% power at 0.05 significance level in a logistic regression model) and based on prior experience with metastatic animal models (Cancer Cell . 2015 Feb 9;27(2):177-92. doi: 10.1016/j.ccell.2014.11.025.) For in vivo experiments, sample size of n=7-10 was typical of experiments and was used in the study. For in vitro experiments, we used sample sizes containing 3 or more biological replicates which can provide adequate statistical power in biochemical analysis. All sample sizes were listed in the corresponding figure legend or figures.
Data exclusions	No data was excluded from the study.
Replication	Biological replicates of each experiment in each panel were indicated in the figure legends, and all attempts at replication were successful.
Randomization	Animals were randomly assigned to the treatments. For in vitro experiments, cells were prepared, treated, processed in a randomly order. The micrographs, FACS analysis are in a random order.
Blinding	Pathologists were blinded to the group allocation during the staining and when assessing the outcomes. No other blinding tests were used in this study since the same investigator designed and performed experiments.

Reporting for specific materials, systems and methods

We require information from authors about some types of materials, experimental systems and methods used in many studies. Here, indicate whether each material, system or method listed is relevant to your study. If you are not sure if a list item applies to your research, read the appropriate section before selecting a response.

Materials & experimental systems

n/a	Involved in the study	
[ ]	[x]	Antibodies
[ ]	[x]	Eukaryotic cell lines
[x]	[ ]	Palaeontology and archaeology
[ ]	[x]	Animals and other organisms
[x]	[ ]	Human research participants
[x]	[ ]	Clinical data
[x]	[ ]	Dual use research of concern

Methods

n/a	Involved in the study	
[x]	[ ]	ChIP-seq
[ ]	[x]	Flow cytometry
[x]	[ ]	MRI-based neuroimaging

Antibodies

Antibodies used	Antibodies used for WB: EZH2(Cell Signaling Technology #5246 dilution 1:1000), H3 (Cell Signaling Technology #4499, dilution 1:1000), pS465/467-Smad2 (Cell Signaling Technology #3108 dilution 1:1000), Smad2 (Cell Signaling Technology #5339 dilution 1:1000), Smad3 (Cell Signaling Technology #9513, dilution 1:1000), Smad4 (Cell Signaling Technology #46535 dilution 1:1000), pT180/Y182-p38 (Cell Signaling Technology #4511 dilution 1:1000), p38 (Cell Signaling Technology #8690 dilution 1:1000), pY397-FAK (Cell Signaling Technology #3283 dilution 1:1000), FAK (Cell #3285 dilution 1:1000), FLAG (Cell Signaling Technology #14793 dilution

1:1000), b-actin (Sigma-Aldrich A5441 dilution 1:5000); TGFbRI (Abcam, ab31013, dilution1:1000), TGFbRI (Millipore #ABF17-I, dilution 1:1000), TGFbRII (Abcam, ab184948, dilution 1:1000), integrin b1 (Santa cruz, sc-8978, dilution 1:1000), phospho-tyrosine (BD Biosciences #610000, dilution 1:1000).
Antibodies used for IP or ChIP: FLAG (Cell Signaling Technology #14793 dilution 1:100); IgG (Santa cruz, sc-2025, sc-2027, dilution is as same as the target antibody); FAK (Cell Signaling Technology #3285 dilution 1:100), TGFbRI (Abcam, ab31013, dilution1:100), EZH2 (Cell Signaling Technology #5246 dilution 1:100), RNA Pol II (Novus Biologicals, NB200-598, 1:100); H3K27me3 (Millipore #07-449, dilution 1:200), pY397-FAK (Cell Signaling Technology #3283 dilution 1:100), integrin b1 (Santa cruz, sc-9970, dilution 1:100).
Antibodies used for IHC cleaved caspase 3 (Cell Signaling Technology, #9664, dilution 1:1000), Ki67 (Abcam, ab15580, dilution to 1μg/ml); PTHLH (R&D Systems, #MAB6734, dilution to 10 μg/ml), pY397-FAK (Cell Signaling Technology #3283 dilution 1:100), pS465/467-Smad2 (Cell Signaling Technology #3108 dilution 1:100), H3K27me3 (Millipore #07-449, dilution 1:200), TGFβ1 (LSBio Company, LS-B14345, dilution to 10 μg/ml).

Validation

All antibodies are commercially available and are stated to be tested by the manufacturer for species reactivity to human or mouse. The statements and validation data for each primary antibody for the species and application are also available on the manufacturers website. In addition antibodies were monitored in house to detect protein of the reported size by western blot in over expression and/or knock down experiments.
All antibodies are commercially available and are stated to be tested by the manufacturer for species reactivity to human or mouse. The statements and validation data for each primary antibody for the species and application are also available on the manufacturers website. In addition antibodies were monitored in house to detect protein of the reported size by western blot in over expression and/or knock down experiments.
EZH2 (Cell Signaling Technology #5246) for human and mouse. Application: WB, ChIP https://www.cellsignal.com/products/primary-antibodies/ezh2-d2c9-xp-rabbit-mab/5246
H3 (Cell Signaling Technology #4499) for human and mouse. Application: WB. https://www.cellsignal.com/products/primary-antibodies/histone-h3-d1h2-xp-rabbit-mab/4499
pS465/467-Smad2 (Cell Signaling Technology #3108) for human and mouse. Application: WB. https://www.cellsignal.com/products/primary-antibodies/phospho-smad2-ser465-467-138d4-rabbit-mab/3108
Smad2 (Cell Signaling Technology #5339) for human and mouse. Application: WB. https://www.cellsignal.com/products/primary-antibodies/smad2-d43b4-xp-rabbit-mab/5339
Smad3 (Cell Signaling Technology #9513) for human and mouse. Application: WB. https://www.cellsignal.com/products/primary-antibodies/smad3-antibody/9513
Smad4 (Cell Signaling Technology #46535) for human and mouse. Application: WB https://www.cellsignal.com/products/primary-antibodies/smad4-d3r4n-xp-rabbit-mab/46535
pT180/Y182-p38 (Cell Signaling Technology #4511) for human and mouse. Application: WB. https://www.cellsignal.com/products/primary-antibodies/phospho-p38-mapk-thr180-tyr182-d3f9-xp-rabbit-mab/4511
p38 (Cell Signaling Technology #8690) for human and mouse. Application: WB. https://www.cellsignal.com/products/primary-antibodies/p38-mapk-d13e1-xp-rabbit-mab/8690
FLAG (Cell Signaling Technology #14793) for all, for human and mouse. Application: WB, IP. https://www.cellsignal.com/products/primary-antibodies/dykddddk-tag-d6w5b-rabbit-mab-binds-to-same-epitope-as-sigma-s-anti-flag-m2-antibody/14793
pY397-FAK (Cell Signaling Technology #3283) for human and mouse. Application: WB. https://www.cellsignal.com/products/primary-antibodies/phospho-fak-tyr397-antibody/3283
FAK (Cell Signaling Technology #3285) for human and mouse. Application: WB. https://www.cellsignal.com/products/primary-antibodies/fak-antibody/3285
b-actin (Sigma-Aldrich A5441) for human and mouse. Application: WB. https://www.sigmaaldrich.com/US/en/product/sigma/a5441
TGFbRI (Abcam, ab31013) for human and mouse. Application: WB, IP. https://www.abcam.com/tgf-beta-receptor-i-antibody-ab31013.html
TGFbRI (Millipore #ABF17-I) for human and mouse. Application: WB, FACS. https://www.emdmillipore.com/US/en/product/Anti-TGF-beta-Receptor-Antibody-type-I,MM_NF-ABF17-I?ReferrerURL=https%3A%2F%2Fwww.google.com%2F
TGFbRII (Abcam, ab184948) for human. Application: WB. https://www.abcam.com/tgf-beta-receptor-ii-antibody-epr14673-ab184948.html
phospho-tyrosine (BD Biosciences #610000) for human and mouse. Application: WB https://www.bdbiosciences.com/en-us/products/reagents/microscopy-imaging-reagents/immunofluorescence-reagents/purified-mouse-anti-phosphotyrosine.610000
integrin b1 (Santa cruz, sc-9970) for human and mouse. Application: WB, IHC https://www.scbt.com/p/integrin-beta1-antibody-4b7r
cleaved caspase 3 (Cell Signaling Technology, #9664) for human and mouse. Application: WB, IHC https://www.cellsignal.com/products/primary-antibodies/cleaved-caspase-3-asp175-5a1e-rabbit-mab/9664
RNA Pol II (Novus Biologicals, NB200-598) for human and mouse. Application: WB, ChIP. https://www.novusbio.com/products/rna-polymerase-ii-polr2a-antibody-4h8_nb200-598
H3K27me3 (Millipore #07-449) for human and mouse. Application: WB, IP. https://www.emdmillipore.com/US/en/product/Anti-trimethyl-Histone-H3-Lys27-Antibody,MM_NF-07-449
Ki67 (Abcam, ab15580) for human and mouse. Application: WB, IHC. https://www.abcam.com/ki67-antibody-ab15580.html
PTHLH (R&D Systems, #MAB6734) for human. Application: IHC https://www.rndsystems.com/products/human-pthlh-pthrp-antibody-677939_mab6734
TGFβ1 (LSBio Company, LS-B14345) for human and mouse. Application: IHC https://www.lsbio.com/antibodies/ihc-plus-tgfb1-antibody-tgf-beta-1-antibody-310-390-aa-c-terminal-elisa-if-immunofluorescence-ihc-wb-western-ls-b14345/503959

Eukaryotic cell lines

Policy information about cell lines

Cell line source(s)

The human breast cancer cell lines MDA-MB-231 and MCF7 and mouse mammary tumor cell line 4T1 were purchased from

	the ATCC. The MDA-MB-231 subline 231-1566 was provided by Dr. Hung's lab. HEK 293FT cells were purchased from Thermo Fisher Scientific. The murine osteoblast cell line MC3T3 was obtained from Dr. Sue-Hwa Lin's lab7 and the murine preosteoclast cell line RAW 264.7 was obtained from the ATCC,
Authentication	These cell lines are authenticated by the MD Anderson Cancer Center Characterized Cell Line Core Facility by STR profiling.
Mycoplasma contamination	All cell lines were tested for and found to be free of mycoplasma contamination.
Commonly misidentified lines (See ICLAC register)	NO commonly misidentified cell lines.

Animals and other organisms

Policy information about studies involving animals; ARRIVE guidelines recommended for reporting animal research

Laboratory animals	Athymic NCR nu/nu mice were purchased from The Jackson Laboratory. The mice were exposed to a 12h light/ dark cycle at 22–24°C with 50%-60% humidity, bred as specified-pathogen-free and given free access to food and water. The number of mice used in each experimental group was determined by power analysis, and mice were grouped randomly for each experiment. Female mice were used for breast cancer and mammary tumor experiments. Bone metastases were induced by intracardiac or intratibia injections. 6-8-week-old athymic NCR nu/nu mice were used in these experiments.
Wild animals	No wild animals was used in this study.
Field-collected samples	No field-collected samples was used in this study.
Ethics oversight	All animal experiments were carried out in accordance with protocols approved by the Institutional Animal Care and Use Committee of The University of Texas MD Anderson Cancer Center. All relevant procedures were compliant with ethical regulation.

Note that full information on the approval of the study protocol must also be provided in the manuscript.

Flow Cytometry

Plots

Confirm that:

- [x] The axis labels state the marker and fluorochrome used (e.g. CD4-FITC).
- [x] The axis scales are clearly visible. Include numbers along axes only for bottom left plot of group (a 'group' is an analysis of identical markers).
- [x] All plots are contour plots with outliers or pseudocolor plots.
- [x] A numerical value for number of cells or percentage (with statistics) is provided.

Methodology

Sample preparation	Cells were obtained from cell culture plates by trypsinization, blocking and staining according to the manufacturer's protocol. Cell surface staining was performed at 4°C for 30 min.
Instrument	BD FACSCanto II cytometer (BD Biosciences)
Software	FlowJo analysis
Cell population abundance	No cell sorting was performed for the experiments of flow cytometry.
Gating strategy	The first gating step was to get cells on the basis of the cells' forward scatter (FSC-A) and side scatter (SSC-A) properties. Second, single cells were identified by plotting their FSC-height (FSC-H) by FSC-area (FSC-A). third, FITC+ or APC+ cells were identified by FITC channel or APC channel respectively.

- [x] Tick this box to confirm that a figure exemplifying the gating strategy is provided in the Supplementary Information.
