# Supplementary material for: EZH2 engages TGFβ signaling to promote breast cancer bone metastasis via integrin β1-FAK activation
Source: Nat Commun. 2022 May 10;13:2543. doi: 10.1038/s41467-022-30105-0 (PMC9091212; doi:10.1038/s41467-022-30105-0)

Original Blot

Figure 3a

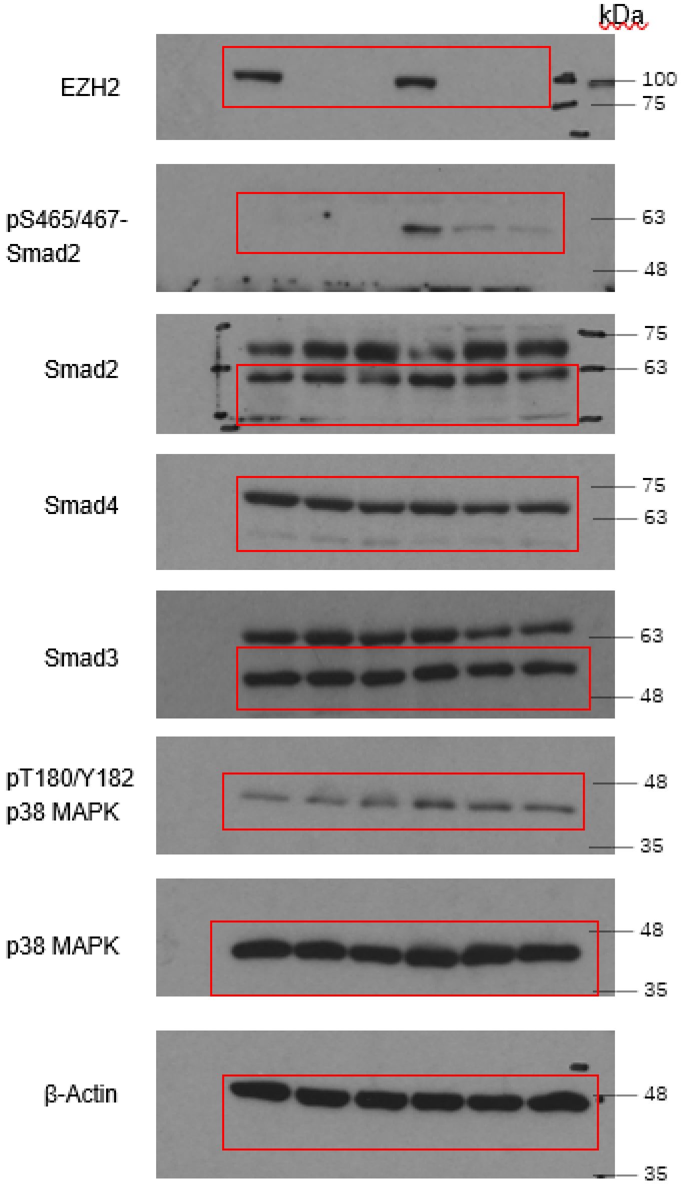

Supplementary Figure 3b

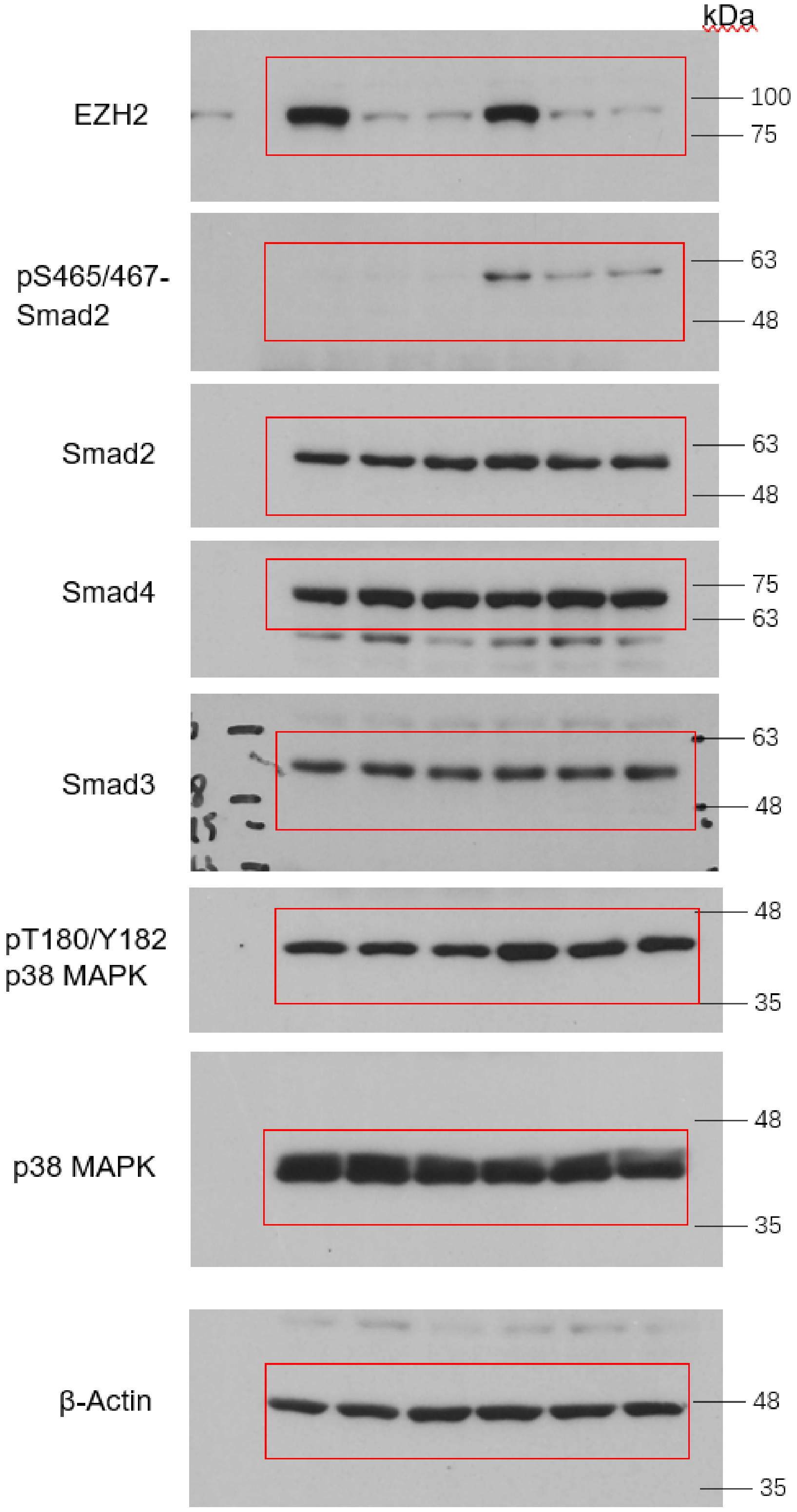

Figure 3b

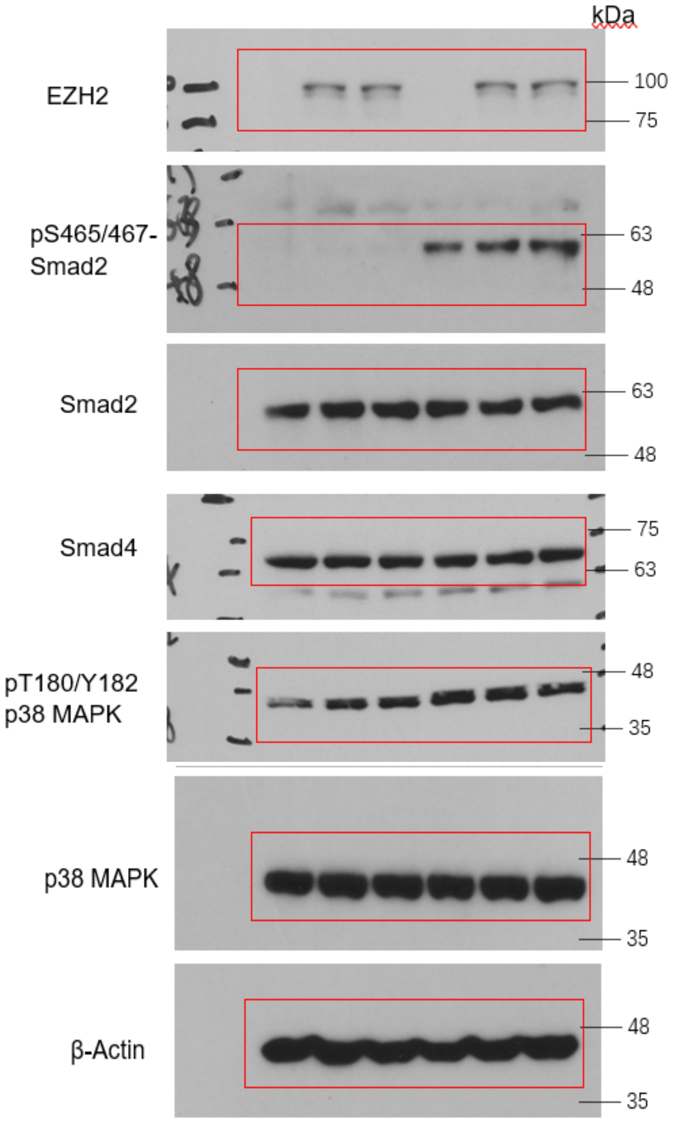
kDa
EZH2
100
75
pS465/467-Smad2
63
48
Smad2
63
48
Smad4
75
63
pT180/Y182 p38 MAPK
48
35
p38 MAPK
48
35
β-Actin
48
35

Figure 3d

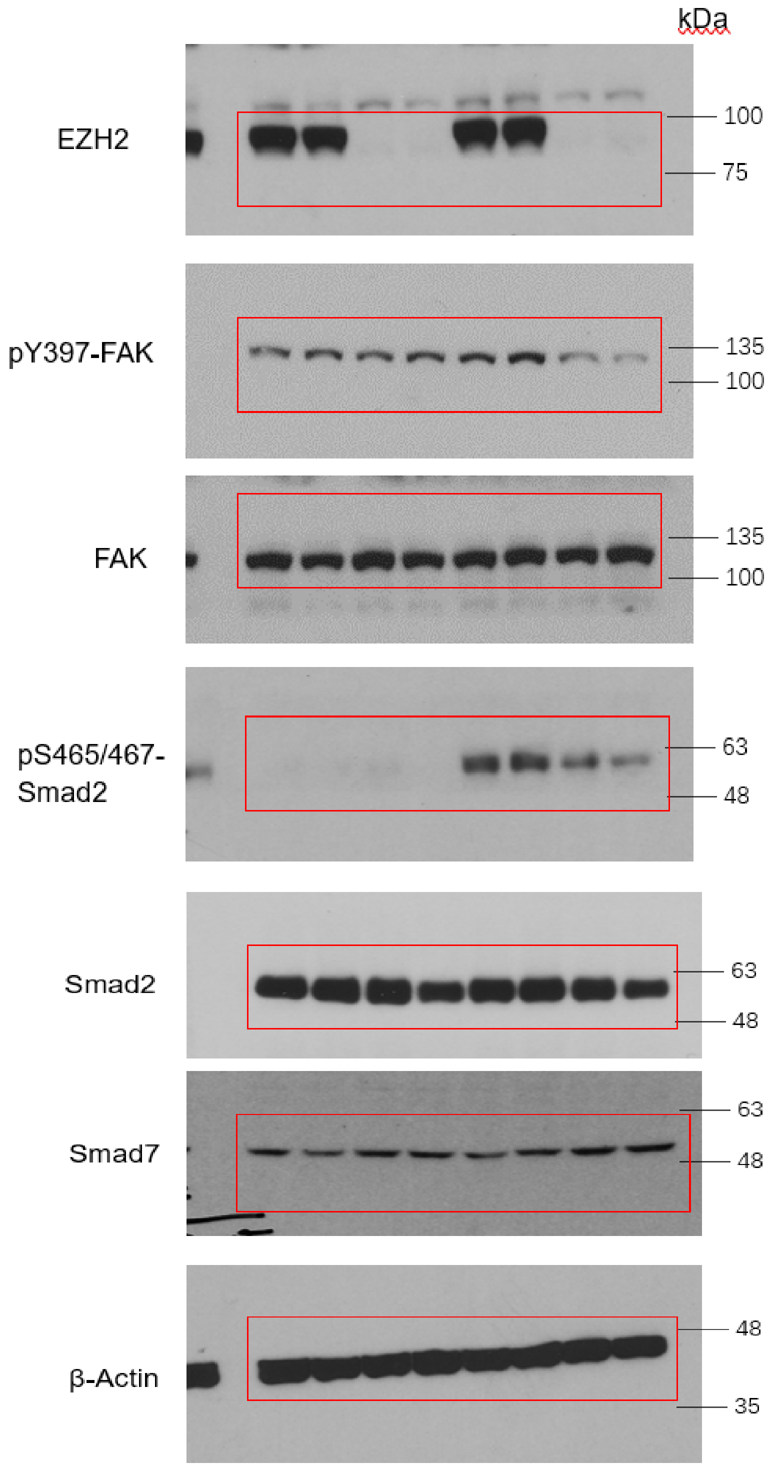

kDa
EZH2
100
75
pY397-FAK
135
100
FAK
135
100
pS465/467-Smad2
63
48
Smad2
63
48
Smad7
63
48
β-Actin
48
35

Supplementary Figure 3i

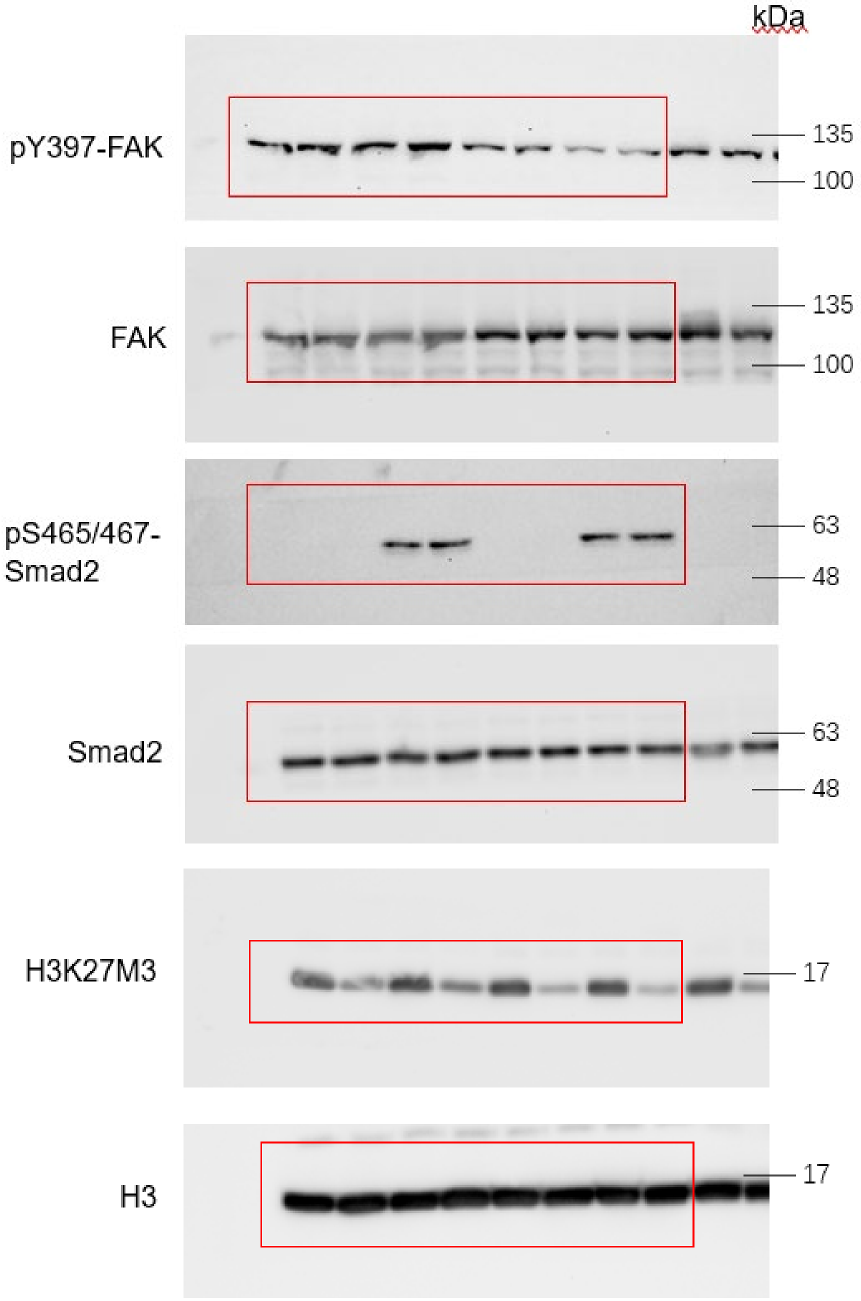

Figure 3e

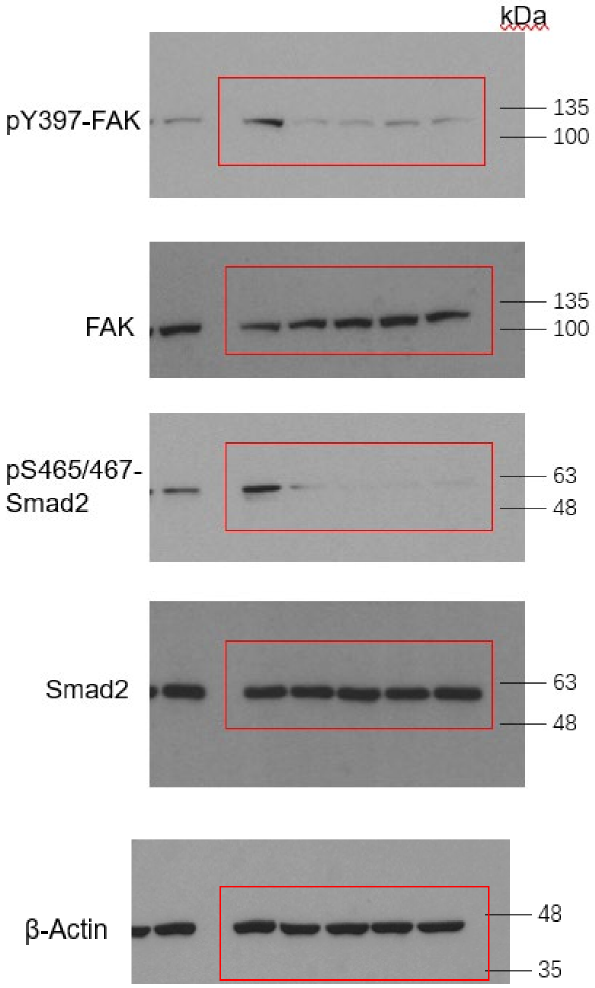

Figure 3f

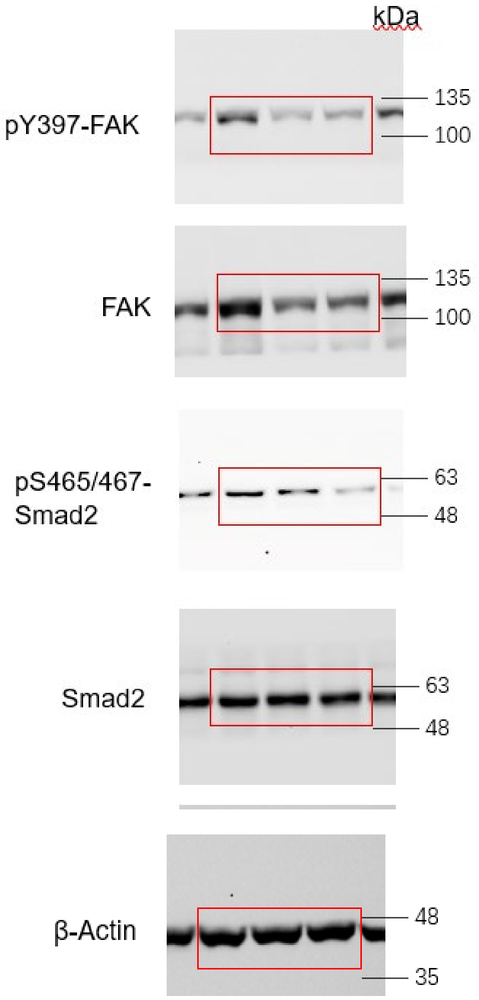

Fig 4a Left

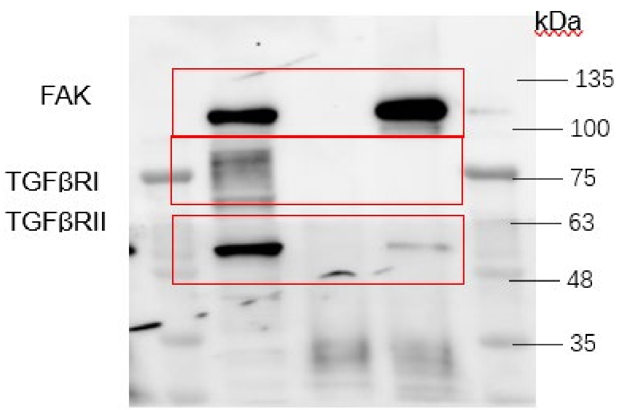

Fig 4a Right

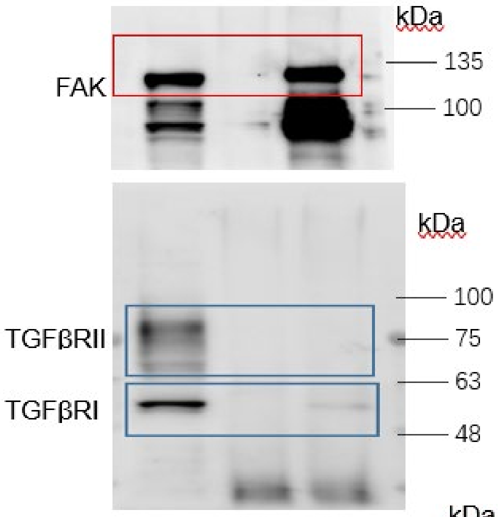

Fig 4b

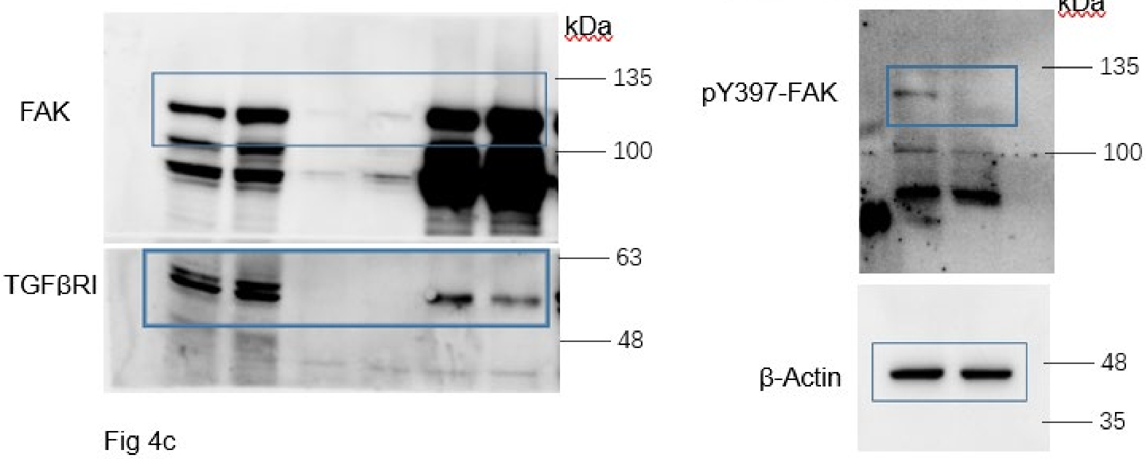

Fig 4c

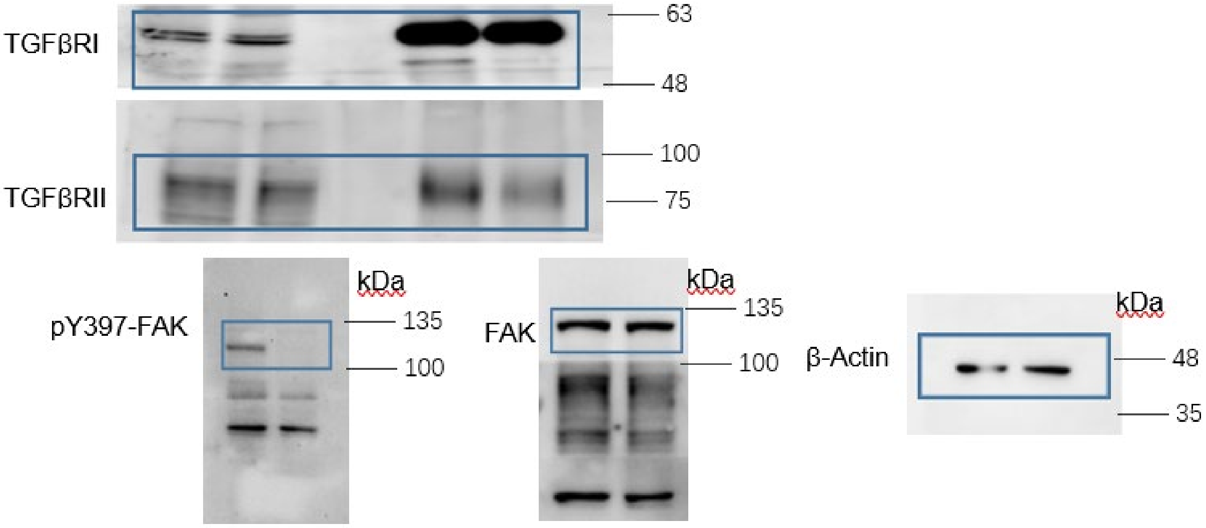

Fig. 4d

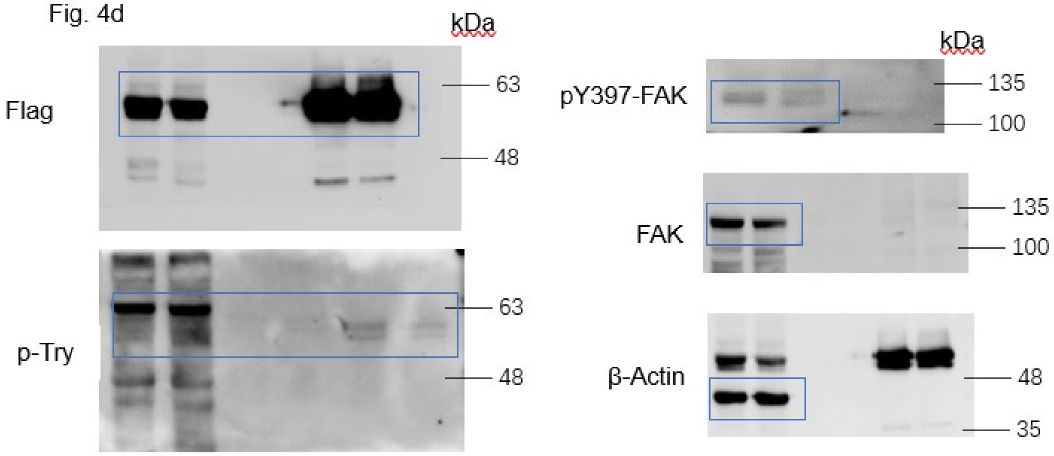

Supplementary Fig. 1a

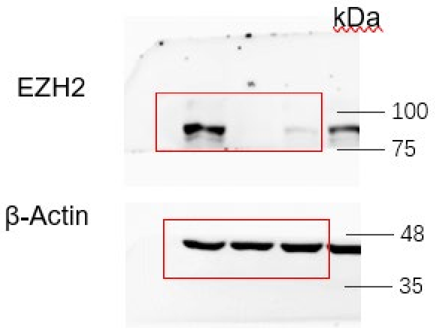

Supplementary Fig. 1b

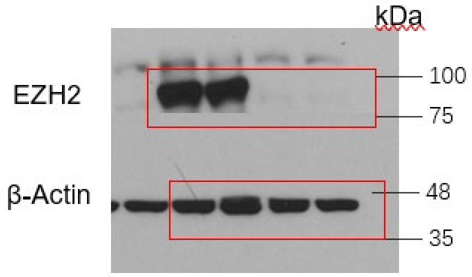

Supplementary Fig. 1c

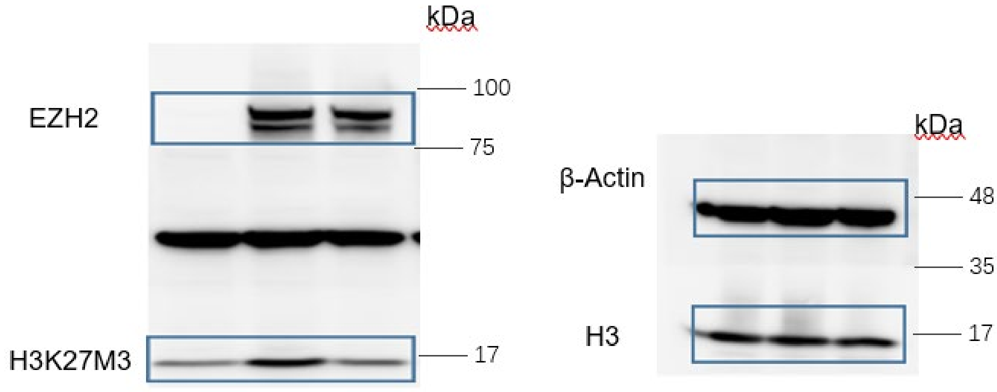

Supplementary Fig. 1d

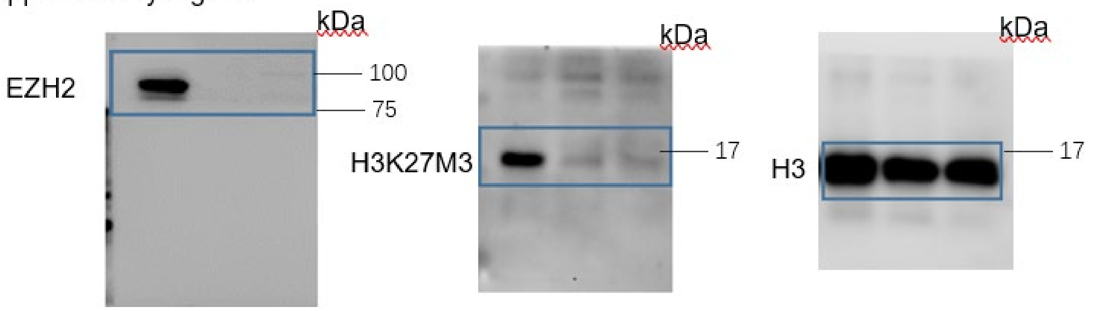

Supplementary Fig. 1k

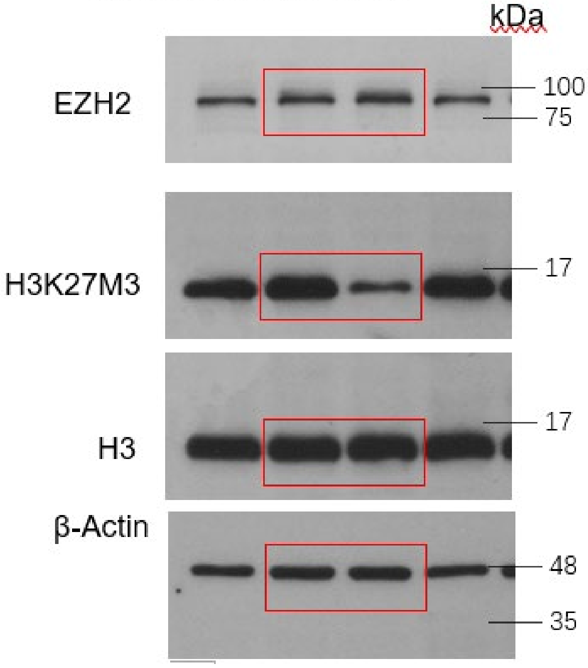

Supplementary Fig. 1o

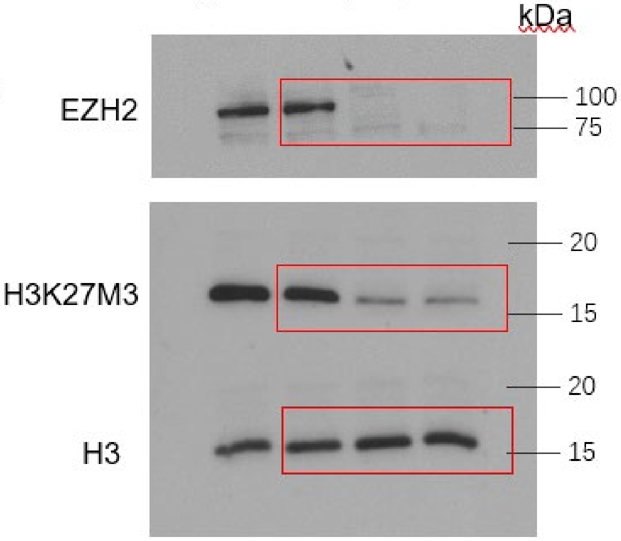

Supplementary Fig 1f

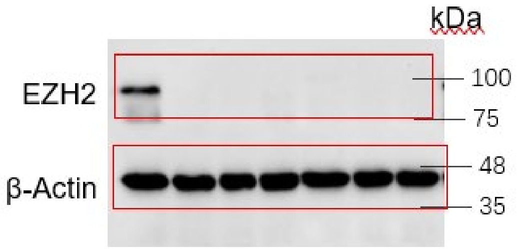

Supplementary Fig. 1r

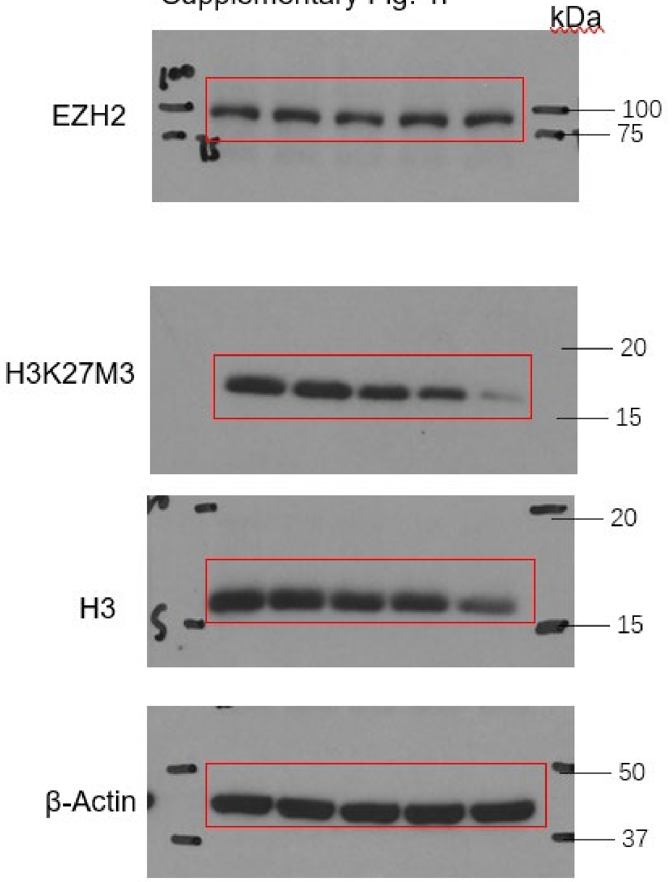

Supplementary Fig. 3a

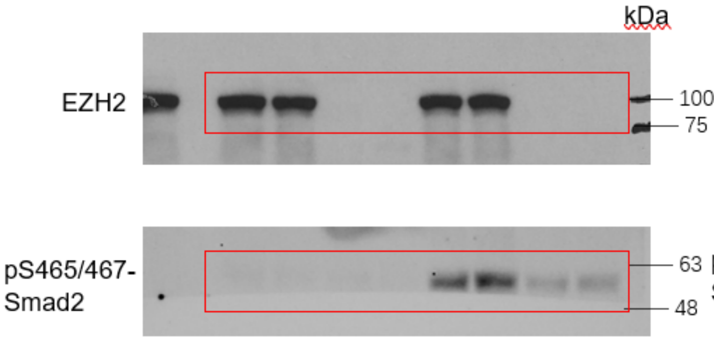

Supplementary Fig. 3c

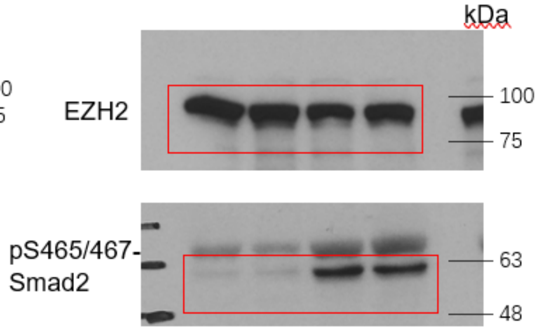

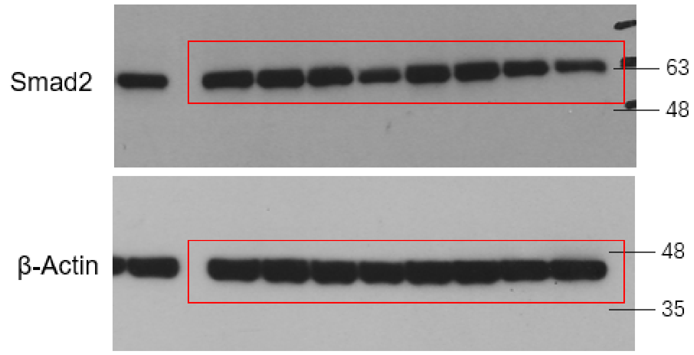

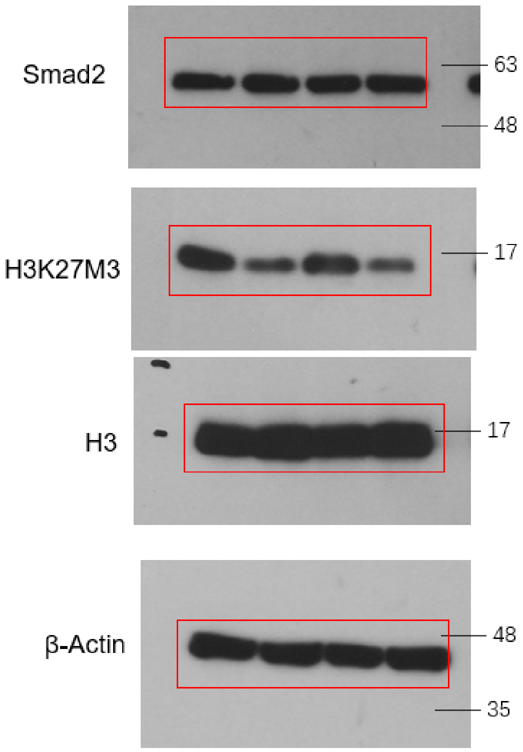

Supplementary Fig. 3e

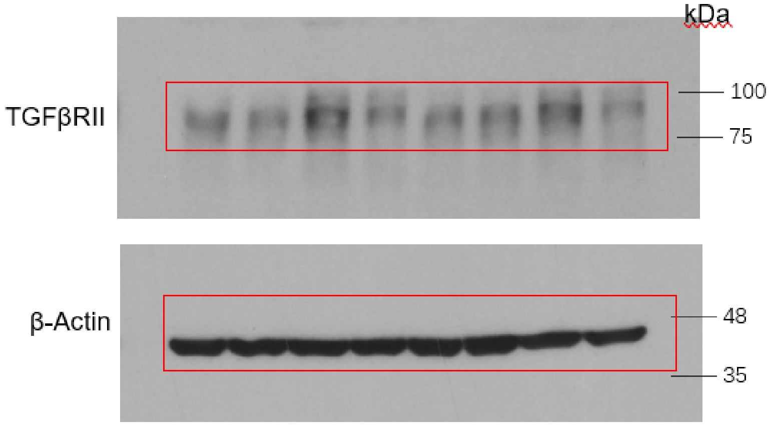

Supplementary Fig. 3h

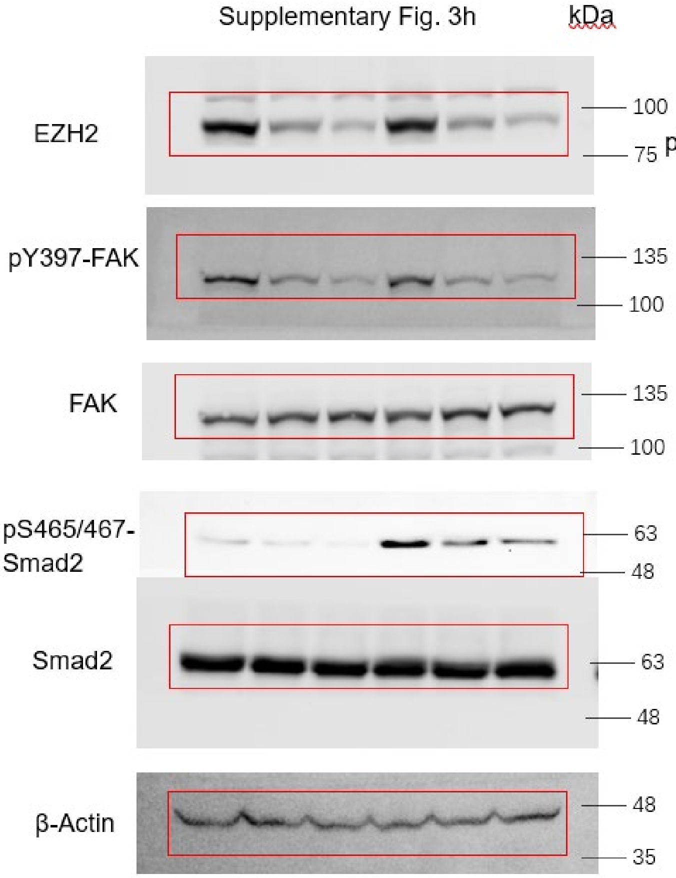

Supplementary Fig. 3j

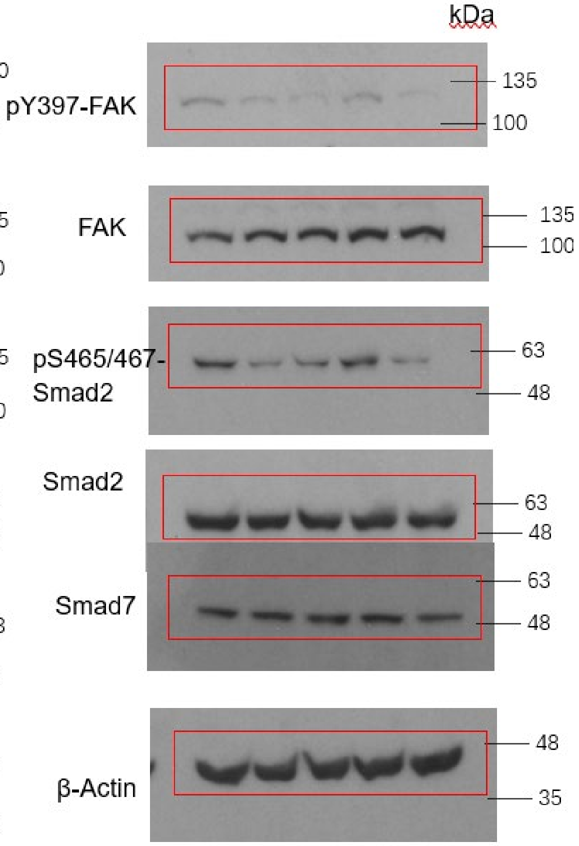

Supplementary Fig. 3l

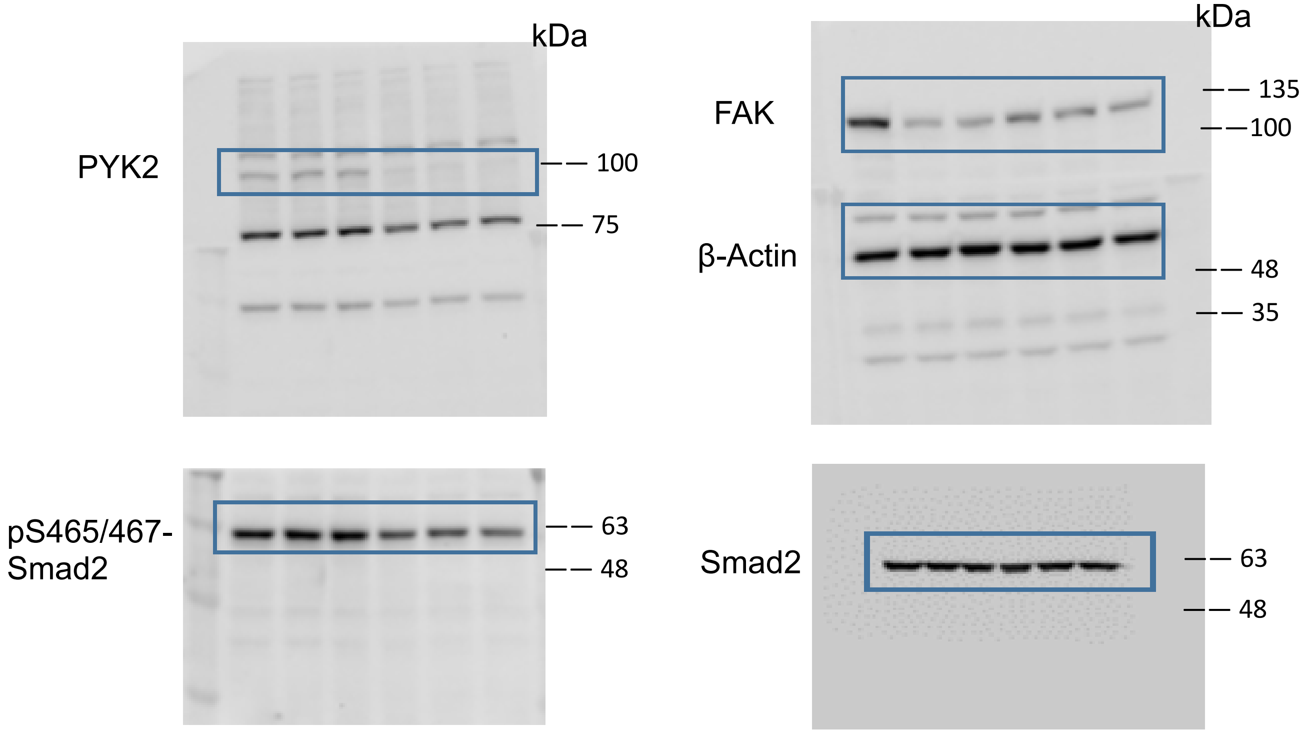

Supplementary Fig. 4a

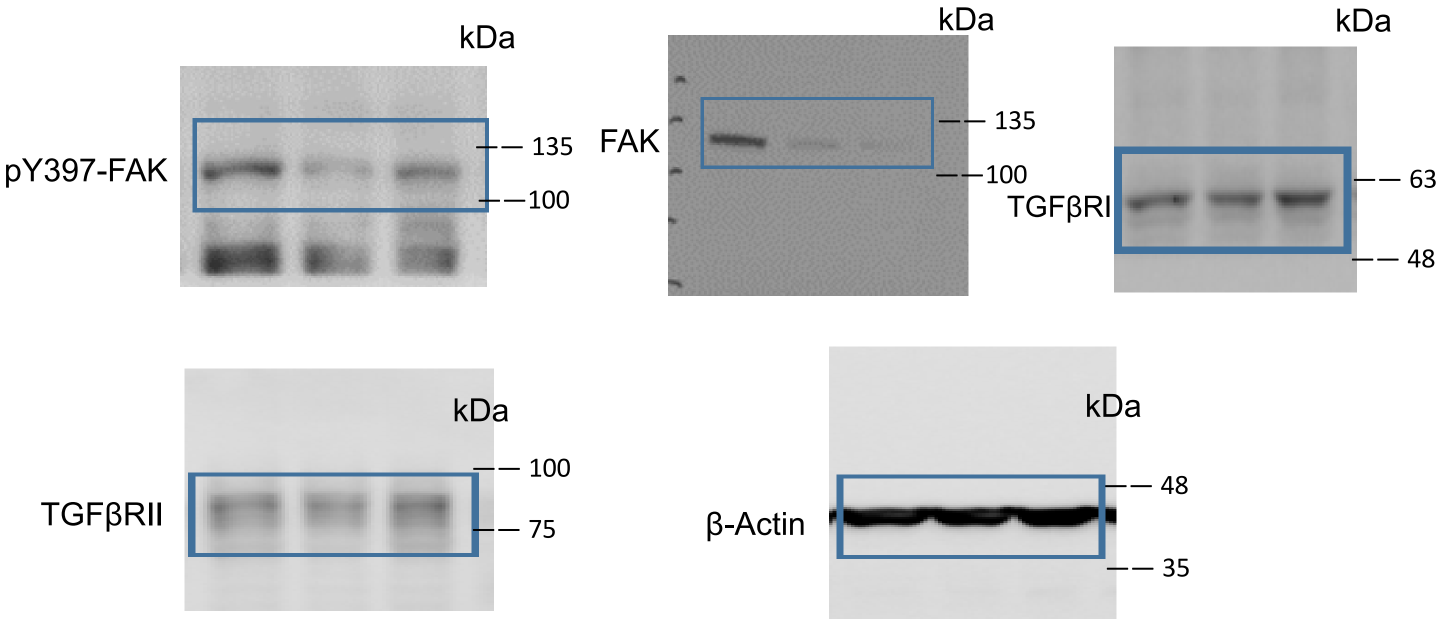

Supplementary Fig. 4b

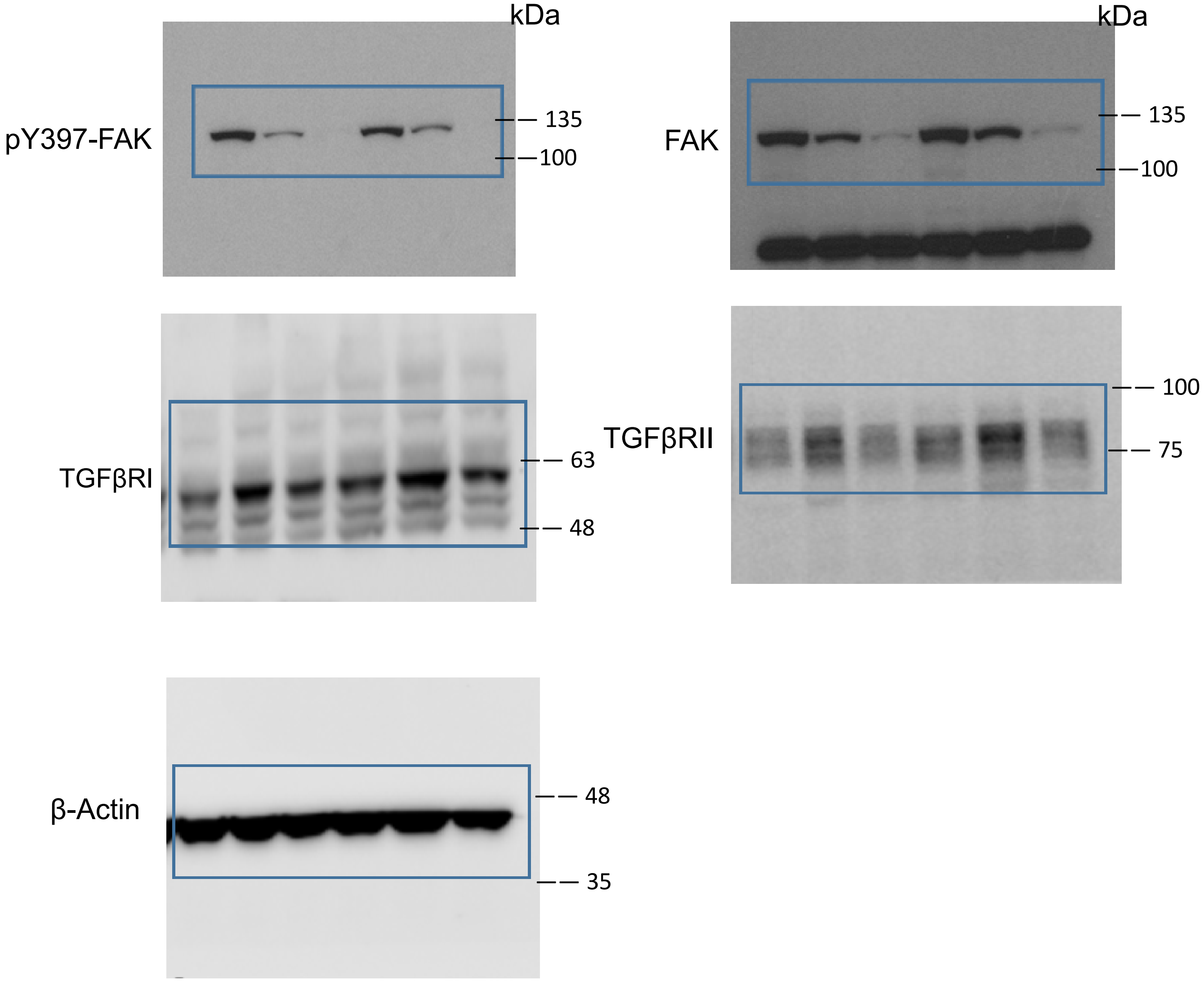

Supplementary Fig. 4c

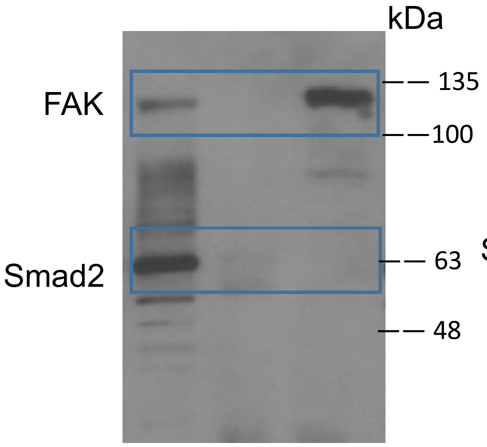

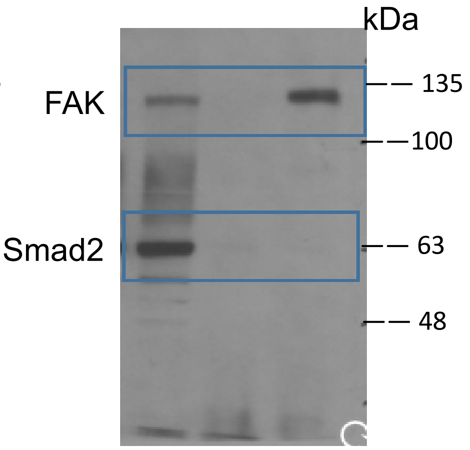

Supplementary Fig. 4d

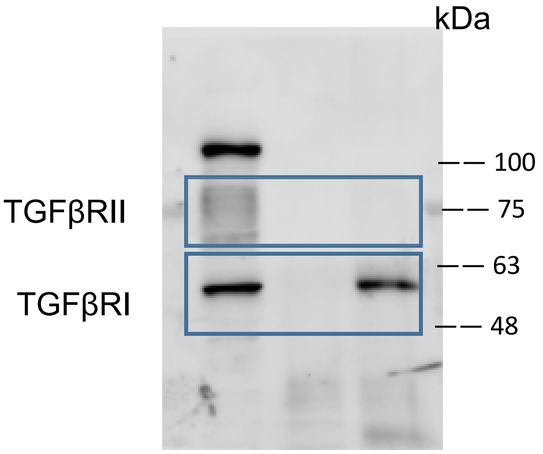

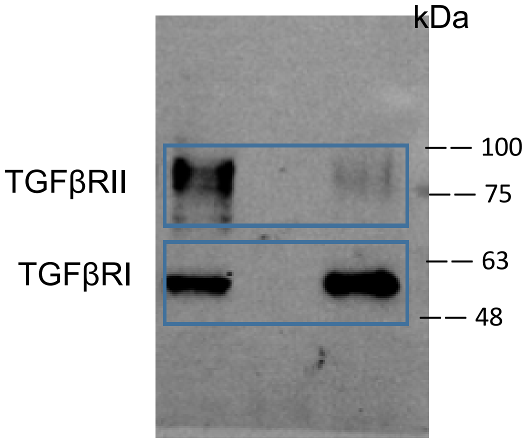

Supplementary Fig. 4e

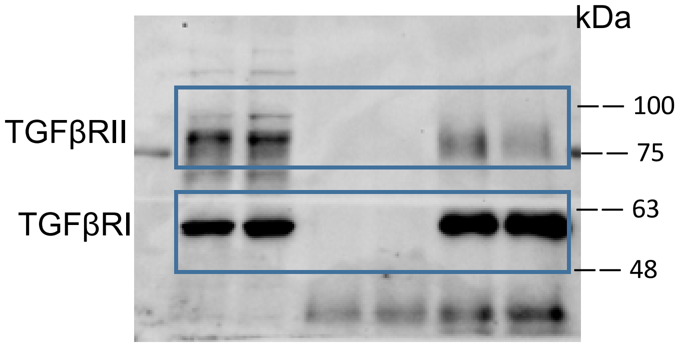

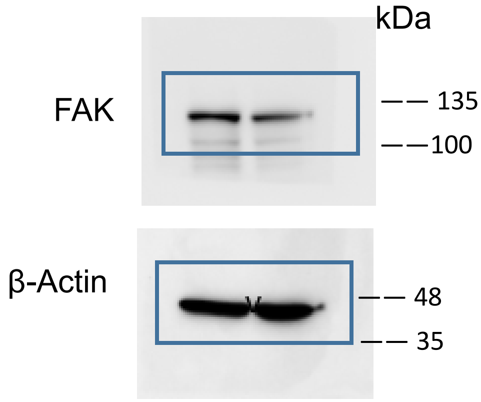

Supplementary Fig. 4f

kDa

kDa

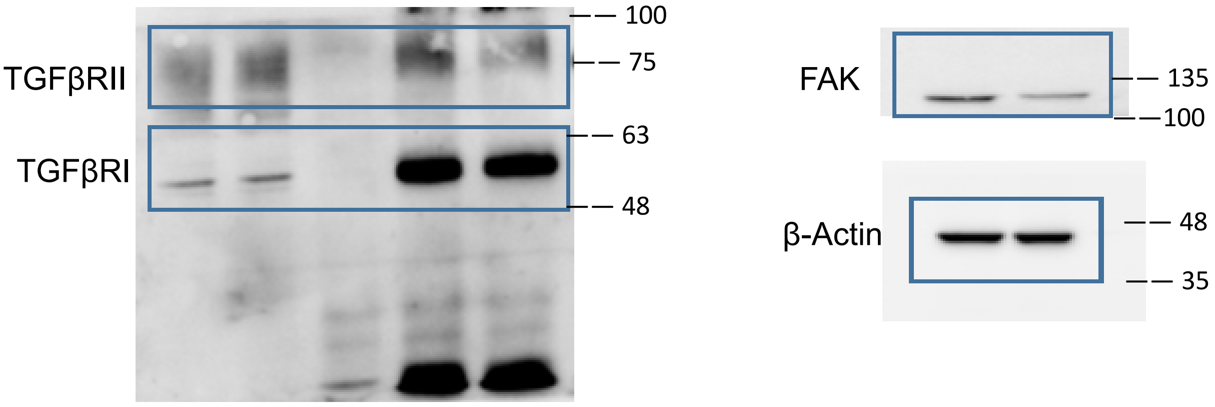

Supplementary Fig. 4g

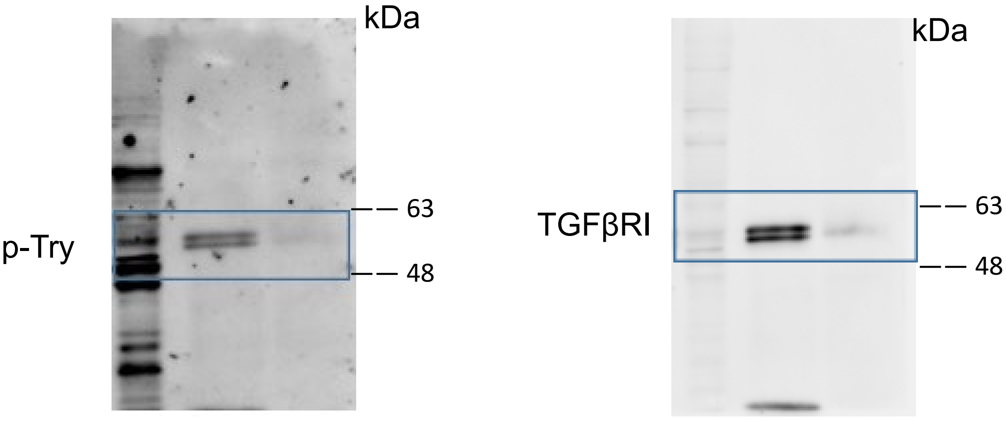

Supplementary Fig. 4h

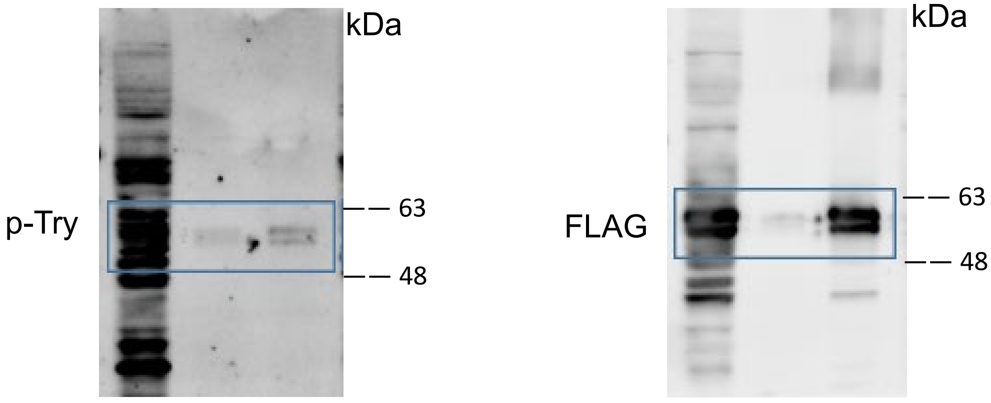

Supplementary Fig. 4j

kDa

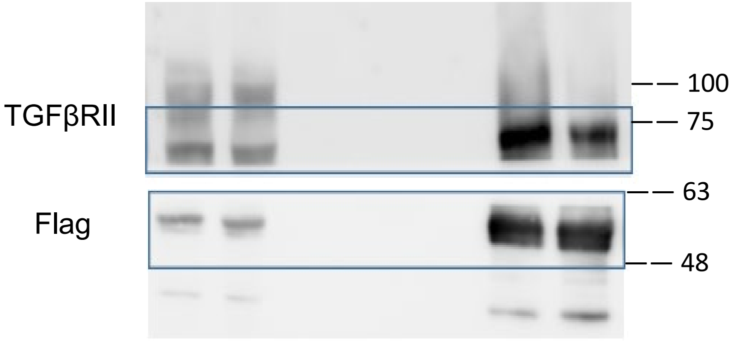

Supplementary Fig. 4k

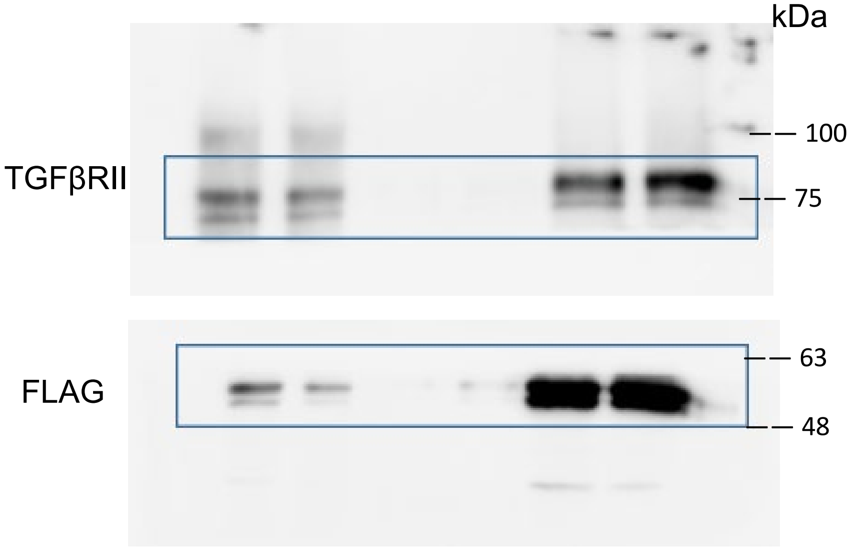

Supplementary Fig. 4l

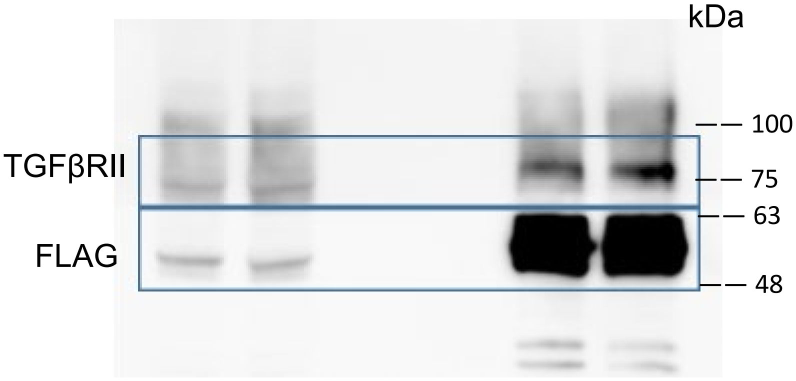

Supplementary Fig. 5b

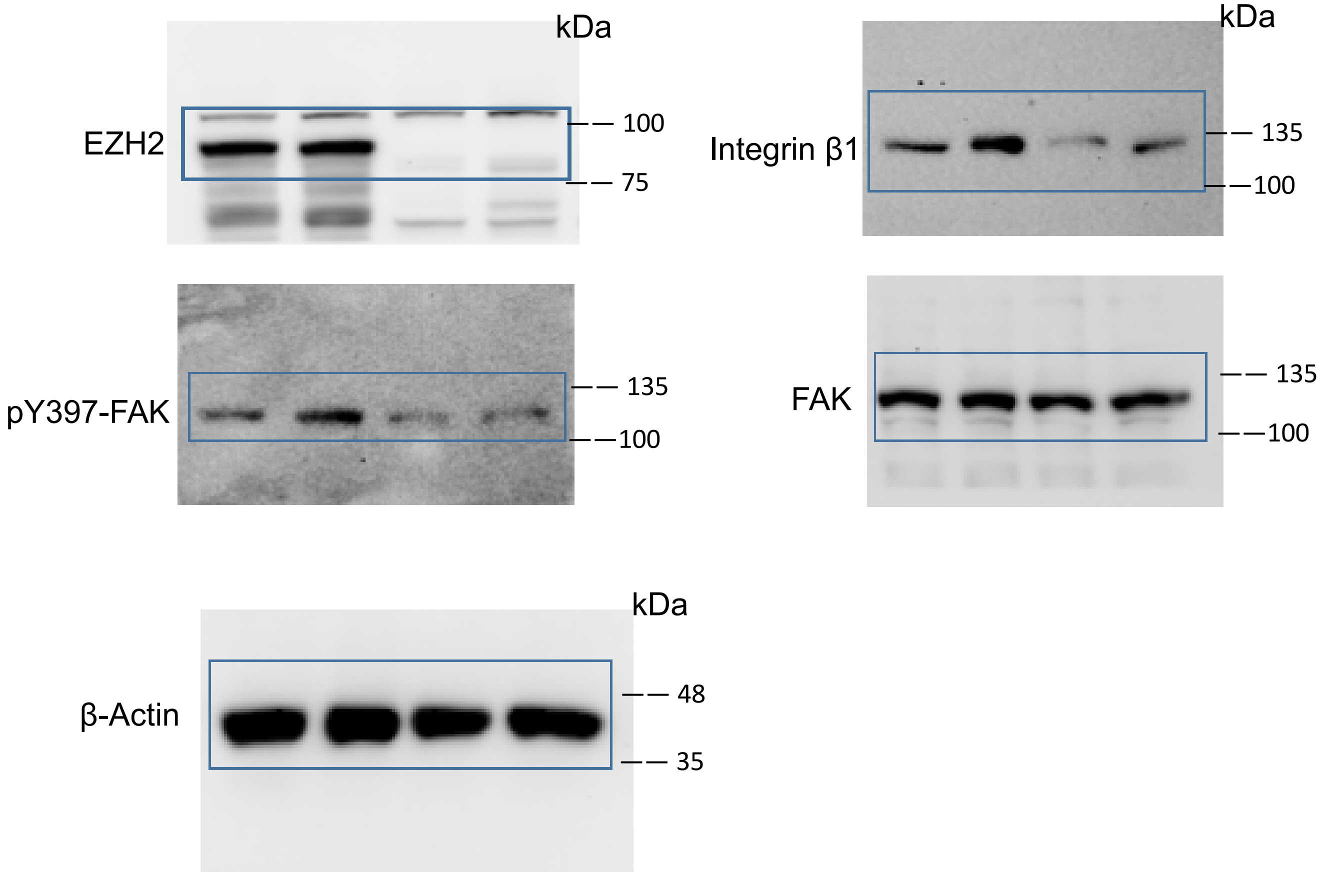

Supplementary Fig. 5c

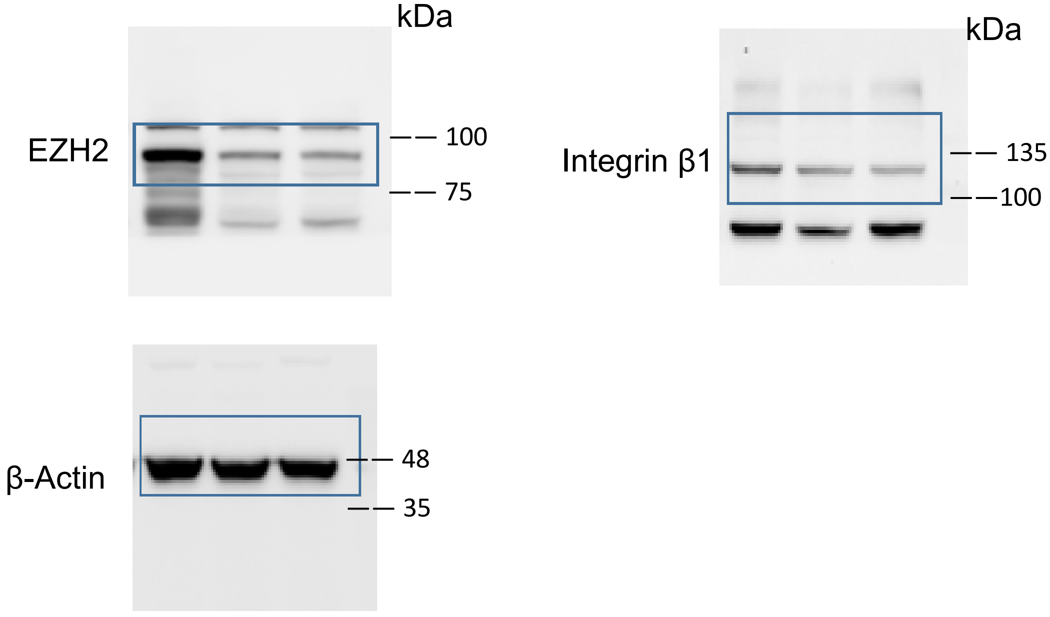

Supplementary Fig. 5h

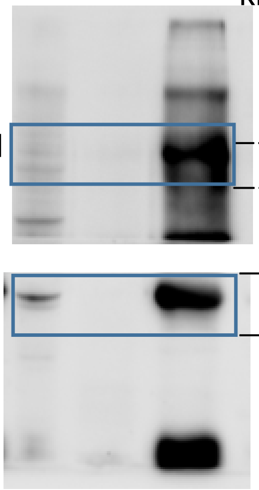

Supplementary Fig. 5i

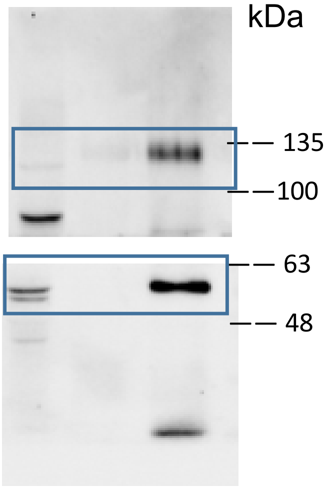

Supplementary Fig. 5k

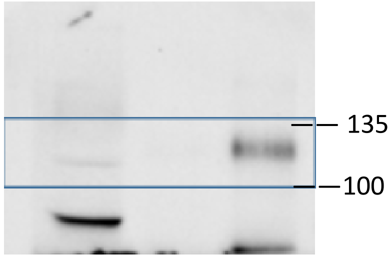

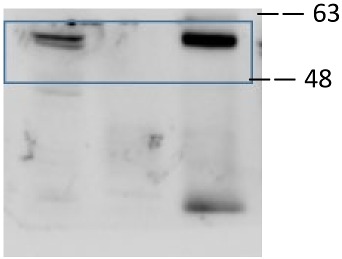

Supplementary Fig. 5l

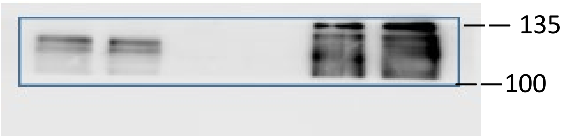

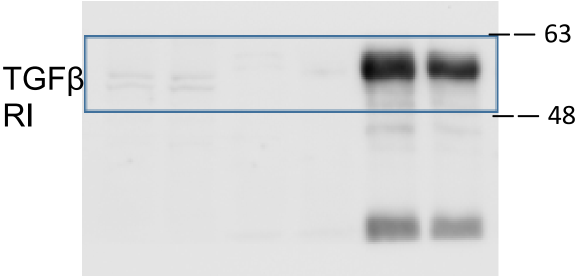

Supplementary Fig. 5n

kDa

kDa

p-Try

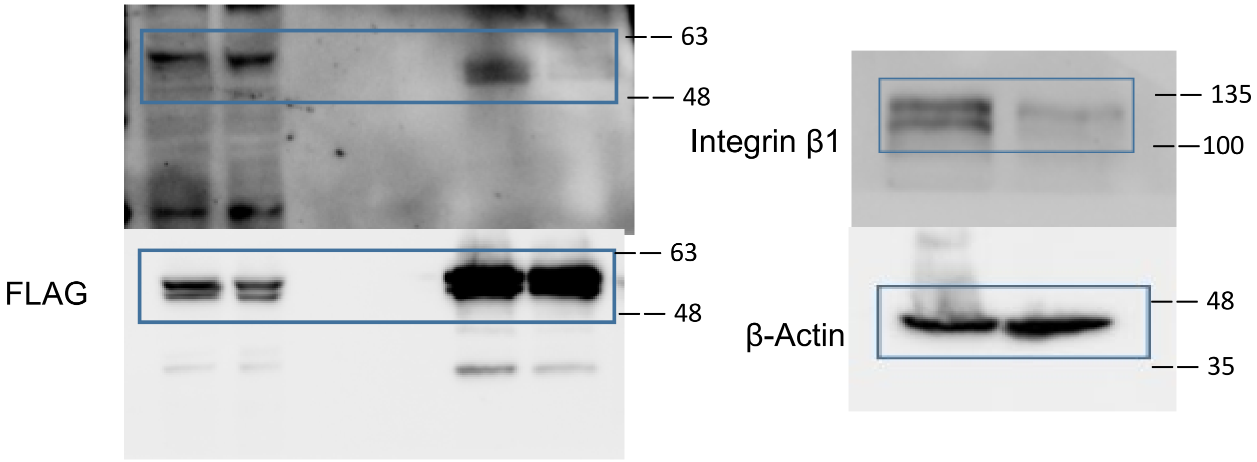

Supplementary Fig. 5o

kDa

kDa

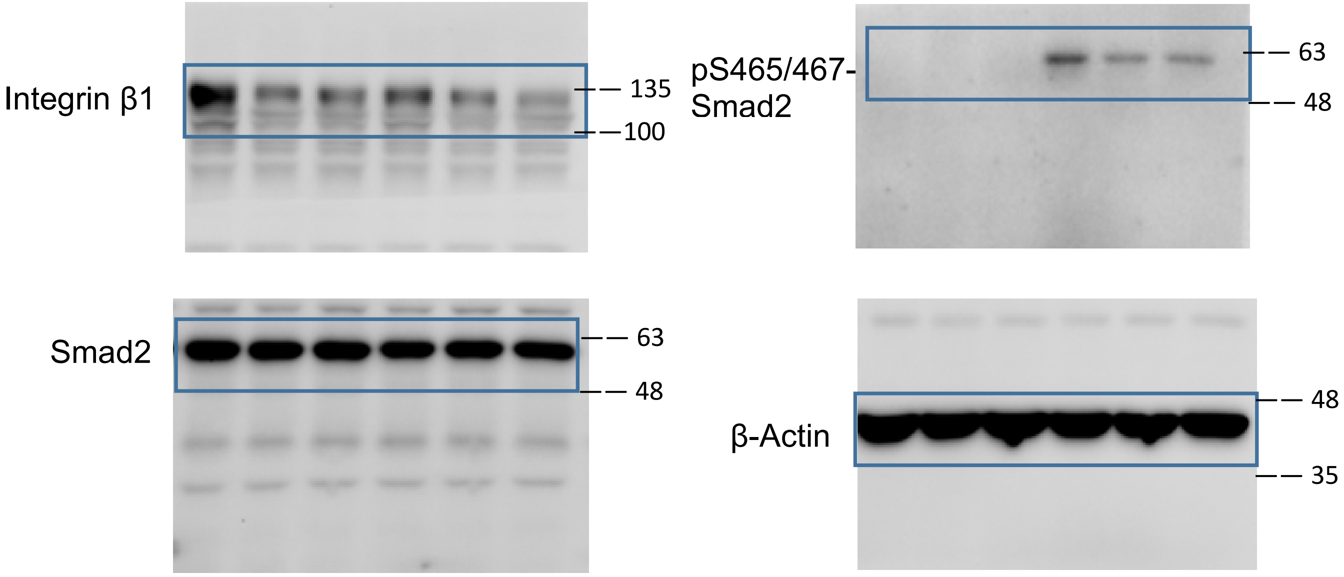

Supplementary Fig. 5p

kDa

kDa

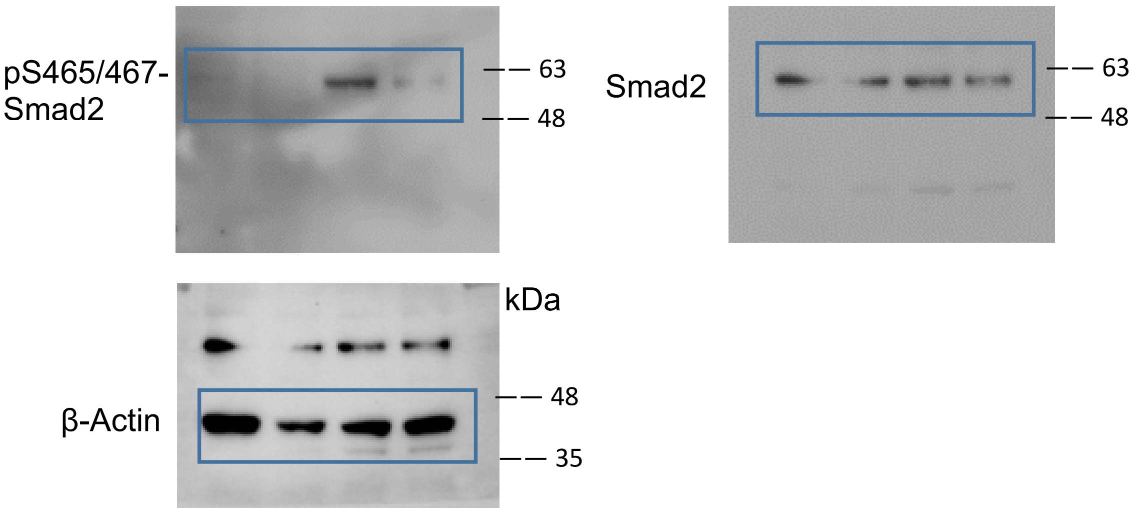

Supplementary Fig. 5q

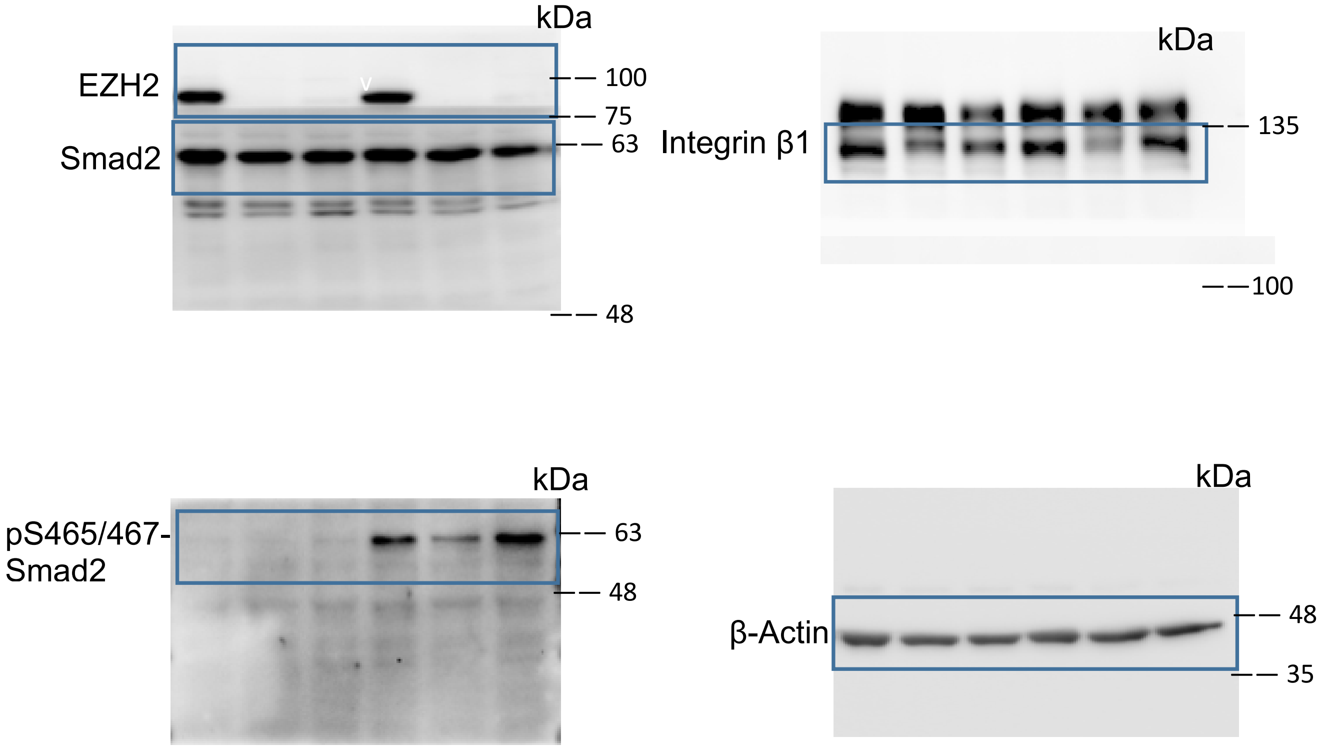

Supplementary Fig. 5r

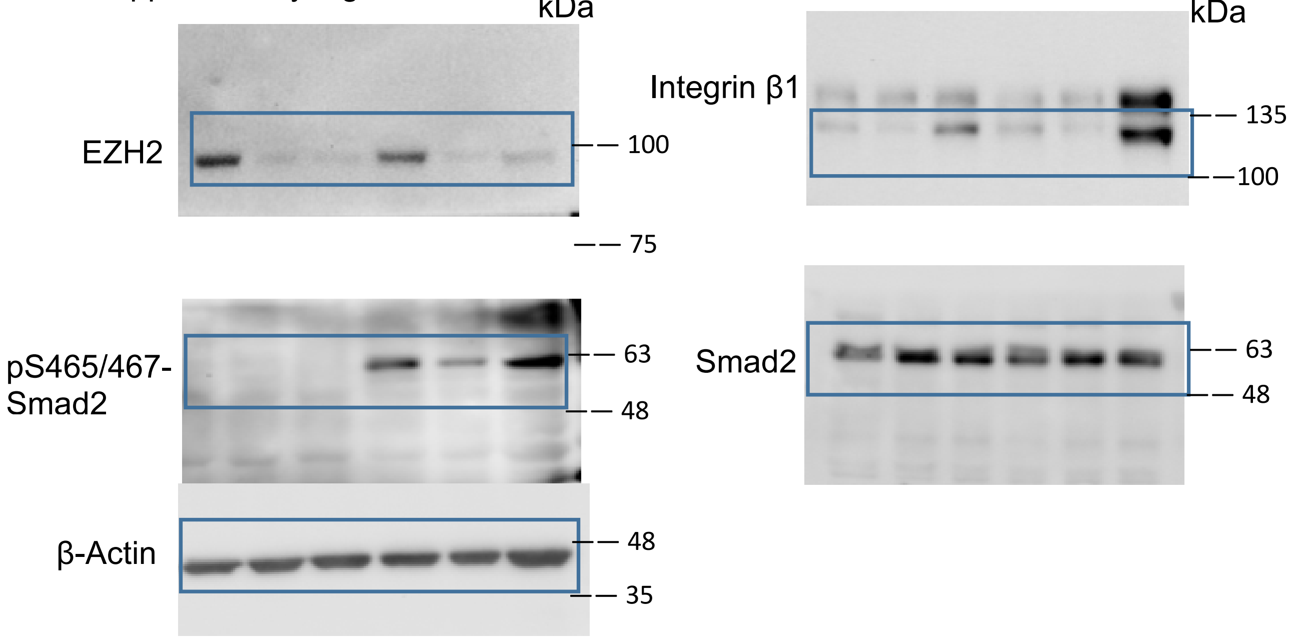

Supplementary Fig. 6a

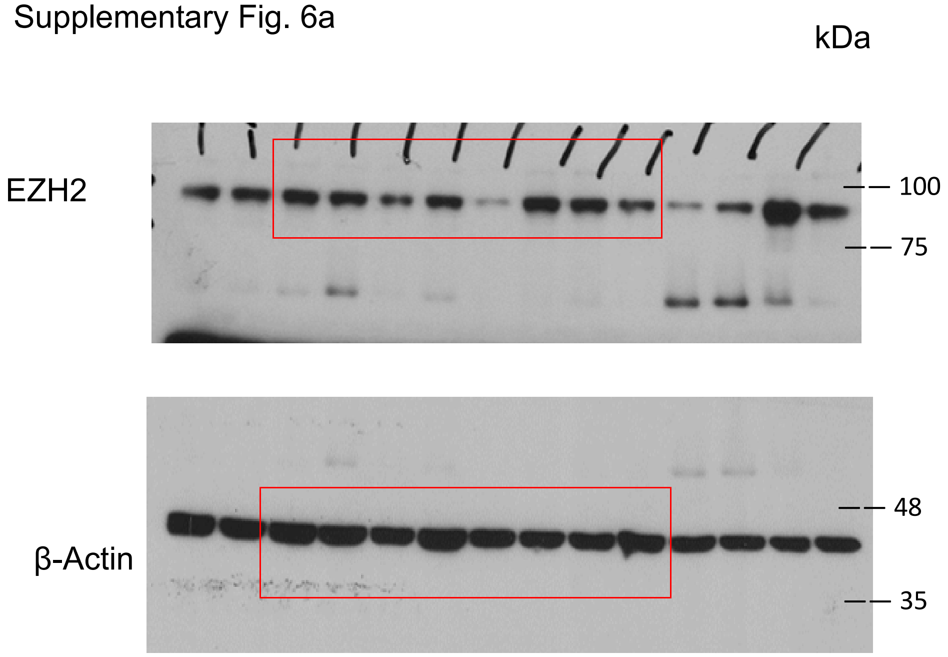

Supplement: Supplementary file 6 — Source Data [file 41467_2022_30105_MOESM6_ESM.zip › Source Data full blots.pdf]
